# BabyBreathe trial: protocol for a randomised controlled trial of a complex intervention to prevent postpartum return to smoking

**DOI:** 10.1136/bmjopen-2023-076458

**Published:** 2023-09-04

**Authors:** Caitlin Notley, Tracey J Brown, Linda Bauld, Allan B Clark, Sharon Duneclift, Vicky Gilroy, Tess Harris, Wendy Hardeman, Richard Holland, Gregory Howard, Mei-See Man, Felix Naughton, Dan Smith, David Turner, Michael Ussher

**Affiliations:** 1 Norwich Medical School, University of East Anglia, Norwich, UK; 2 The Usher Institute, College of Medicine and Veterinary Medicine, The University of Edinburgh, Edinbugh, UK; 3 Norwich Clinical Trials Unit, Norwich Medical School, University of East Anglia, Norwich, UK; 4 Norfolk Healthy Child Programme, Norwich, UK; 5 Institute of Health Visiting, London, UK; 6 Population Health Research Institute, St Georges, University of London, London, UK; 7 School of Health Sciences, University of East Anglia, Norwich, UK; 8 Exeter Medical School, University of Exeter, Exeter, UK; 9 School of Computing Sciences, University of East Anglia, Norwich, UK; 10 Institute for Social Marketing and Health, University of Stirling, Stirling, UK

**Keywords:** Postpartum Period, PREVENTIVE MEDICINE, Primary Health Care

## Abstract

**Introduction:**

Many people quit smoking during pregnancy, but postpartum smoking relapse is common. Maintaining smoking abstinence achieved during pregnancy is key to improving maternal and child health. There are no evidence-based interventions for preventing postpartum smoking relapse. This trial aims to determine whether an intervention to prevent postpartum relapse is effective and cost-effective.

**Methods and analysis:**

A randomised controlled trial of a complex intervention to prevent postpartum smoking relapse (BabyBreathe), with internal pilot, economic and process evaluations. Participants are adults who are pregnant and who report having quit smoking in the 12 months before, or during pregnancy. Participants are eligible if they read and understand English, and provide informed consent. Following consent and biochemical validation of smoking abstinence, participants are randomised to intervention or usual care/control (no specific relapse prevention support). The BabyBreathe intervention consists of manualised advice from a trained member of the health visiting service, health information leaflets for participants and partners, access to the BabyBreathe website and app. At the time of birth, participants are posted the BabyBreathe box and support is provided by text message for up to 12 months postpartum. Target sample size is 880, recruiting across midwifery services at four hubs in England and Scotland and through remote advertising in England, Scotland, Wales and Northern Ireland. Outcomes are collected at 6 and 12 months. The primary outcome is self-reported sustained smoking abstinence at 12 months, carbon monoxide verified. Secondary outcomes include self-reported abstinence, time to relapse, partner smoking status and quality of life.

**Ethics and dissemination:**

The trial was approved by the North West Preston Research Ethics committee (21/NW/0017). Dissemination will include publication in peer-reviewed journals, presentation at academic and public conferences including patient and public involvement and to policymakers and practitioners.

**Trial registration number:**

ISRCTN70307341

**Strengths and limitations of this study**

- This is the largest international trial of a postpartum smoking relapse prevention intervention, specifically developed to support sustained postpartum smoking abstinence.
- The intervention (BabyBreathe) is theory based, drawing on behaviour change techniques, systematic reviews of existing evidence and extensive patient and public involvement.
- An embedded mixed-methods process evaluation will assess implementation, mechanisms of impact and contextual influences, as well as acceptability and which elements of the intervention are perceived to be most effective, for which women, in which circumstances.
- The study is resource intensive and is limited by the capacity of clinical services. The trial protocol allows flexible options for recruitment and intervention delivery to support clinical teams in delivering the intervention.
- The trial is recruiting across the UK and includes a cost-effectiveness evaluation.

## Introduction

Around a quarter of UK women report smoking in the year before pregnancy.^1–3^ More women quit smoking during pregnancy than at any other time, with as many as 45% able to ‘spontaneously quit’.^4^ However, there are marked health inequalities, as younger mothers and women with lower income are both less likely to quit and more likely to relapse.^5 6^ There is a unique opportunity to help women who cease smoking in pregnancy to quit permanently. Most women who quit smoking wish to remain abstinent after the birth; however, up to three-quarters of spontaneous quitters return to smoking within 6 months.^7^ Postpartum relapse is a major public health problem; yet there are no evidence-based interventions, and no routine support is offered to prevent relapse.^8^ The National Health Service (NHS) Long Term Plan prioritises smoking cessation services in pregnancy,^9^ overlooking postpartum support. Supporting sustained abstinence may be critical to reaching the UK government ‘smokefree 2030’ target.^10^ This trial will build on the success of cessation interventions in pregnancy,^11^ by trialling a theory-based relapse prevention intervention developed by our team.^12^

Previous interventions to support sustained smoking abstinence post partum consist of brief and skills-based education, but when pooled, studies overall did not demonstrate effectiveness.^13^ A recent Cochrane review of relapse prevention interventions included postpartum relapse prevention trials as a subgroup. Fifteen studies included postpartum follow-up but there was no significant benefit of interventions.^8^ New approaches are urgently needed to address this global public health issue. The recent Cochrane review concludes that: ‘Future studies may be better advised to focus on alternative approaches not studied extensively or at all so far, such as opportunistic use of nicotine replacement, contingency management, social support, cue exposure (only imaginary exposure has been studied so far), interventions aimed at maintaining abstainers’ morale and awareness of the danger of slips, and so forth’.^8^ Sustained postpartum smoking abstinence has significant health benefits for the mother, as most new mothers will be young enough to minimise long-term harm, particularly from cancers and cardiovascular disease.^14^ Maternal smoking is the primary source of infant and child secondhand smoke exposure,^15 16^ a substantial cause of ill health and mortality.^17^ This has an intergenerational effect: children of smoking mothers are twice as likely to become smokers.^18^ The total NHS annual cost of smoking continuation, or returning to smoking following pregnancy, is estimated to range between £8.1 and £64 million annually for treating maternal health problems alone.^19^ While, in 2015/2016 the cost of admitted patient care in children attributable to passive smoking in England was an additional £5–12 million.^20^

Following our comprehensive intervention development work and patient and public involvement, it is clear that postpartum smoking relapse is a complex problem requiring a multifaceted solution. Our research team have developed a novel intervention combining behavioural, digital and relapse prevention support, ‘BabyBreathe’. The intervention is theory based and uses behaviour change techniques, each supported by available evidence.^21^ The development process involved working with pregnant and post-partum people, families and healthcare professionals to design an intervention that would fit in and work alongside usual care (universal health visiting service in the UK), be feasible to implement in practice and be acceptable.^12^

## Aims and objectives

### Aim

To assess the effectiveness and cost-effectiveness of the BabyBreathe intervention in comparison to usual care, for supporting long-term smoking abstinence for mothers who have recently given birth and have stopped smoking during pregnancy or during the 12 months prior to pregnancy.

### Objectives

1. To run an internal pilot study, with clear stop/go trial embedded criteria, primarily to test recruitment systems.
2. To definitively test the effectiveness of BabyBreathe in comparison with usual care, by comparing smoking abstinence rates at 12 months postpartum between trial groups.
3. To undertake a cost-effectiveness analysis of BabyBreathe in comparison with usual care based on healthcare resource use of mother and infant and maternal health-related quality of life (HRQoL).
4. To undertake an embedded mixed-methods process evaluation to assess delivery, implementation, fidelity and contamination and to identify mechanisms of action by exploring which intervention components may be particularly effective, for which women, in which contexts.

## Methods and analysis

This protocol is reported in accordance with the Standard Protocol Items: Recommendations for Interventional Trials recommendations^22^ and the Template for Intervention Description and Replication (TiDIER) guidelines^23^ (see online supplemental file).

10.1136/bmjopen-2023-076458.supp1 Supplementary data

### Trial design

BabyBreathe is a multicentre, two-arm, superiority, parallel group, individually randomised, controlled trial of a complex intervention to prevent return to tobacco smoking postpartum, with internal pilot, including economic evaluation and process evaluation.

### Study setting

The setting is ‘real world’ with the intervention integrated into, or offered as an adjunct to, standard antenatal and postnatal care. Trial recruitment hubs (Norfolk, London, North East of England, and Lothian, Scotland) have been selected to ensure a diverse sample, with an additional ‘remote’ recruitment hub to maximise recruitment rates (across the UK, including Wales and Northern Ireland).

### Patient and public involvement

Two abstinent postpartum women were involved in development of intervention materials, and are included as members of our trial steering group, to advise on study progress and dissemination.

### Population

We will seek pregnant people who have quit tobacco smoking in the 12 months before or during pregnancy, where smoking abstinence is defined as having stopped smoking for at least 4 weeks prior to recruitment.

#### Inclusion criteria

1. Pregnant people who have stopped smoking completely in the 12 months prior to pregnancy, or at any time during pregnancy.
2. At 26 weeks gestation or any time following this up until birth, participant confirms having not smoked a single puff of a cigarette for at least 4 weeks.
3. Able to read and understand English.
4. Willing and able to give informed consent for participation in the study.
5. Expired carbon monoxide (CO) reading less than four parts per million (ppm).^24^

#### Exclusion criteria

Under the age of 16.

### Recruitment and screening

Multiple recruitment strategies will be used to reach target sample size (n=880). Potential participants will be identified by hospital and community midwives, research midwives (Clinical Research Network,CRN) or sonographers, during routine antenatal appointments (eg, booking appointment, routine scan appointment for dating or fetal anomaly scan) or by screening medical records. Participants may also be identified by smoke-free services, health visitors or by self-referring (eg, via adverts in health or community settings, using targeted online recruitment or media adverts). Potential participants will be screened for eligibility by the midwife (or by other healthcare professionals, in other health settings), or by a study researcher for direct referrals. The screening process can take place at any time during pregnancy, though the target is to identify participants ahead of 26 weeks pregnancy.

Eligible participants will be provided a brief patient information leaflet, either directly or indirectly via an online link, explaining the study and permission will be requested to pass their contact details to the research team. A health professional or a research team member will enter their details into a study database (Research Electronic Data Capture (REDCap)^25^) that will automatically generate a short messaging service (SMS)/email to an electronic patient information sheet and e-consent form containing full reassurance of confidentiality. If participants are unable or unwilling to consent electronically, study researchers will contact potential participants by telephone to complete consent. Once consent is completed, participants will provide further details so they can be contacted from 26 weeks pregnancy with the link to the eligibility confirmation questionnaire.

Participants will be asked to confirm eligibility by replying via a link sent by text or email (according to preference), and will provide their address to enable postage of a CO monitor (iCO monitor, Bedfont) in order to confirm eligibility using an expired CO reading of less than 4 ppm (this is the standard cut-off used in pregnancy).^24^ Participants will be asked to download the study specific CO monitor application (iCOBabyBreathe) which will provide the REDCap database with two CO readings. The highest of the two readings will be recorded. Where CO readings ≥26 weeks gestation are able to take place in person as part of standard care, CO readings may be obtained by a member of the clinical team or a researcher to confirm participant eligibility.

Once the participant has given informed consent and eligibility is confirmed through a CO reading, a link will be automatically generated through text/email to the participant to complete the baseline questionnaire.

### Randomisation

**Figure 1:**
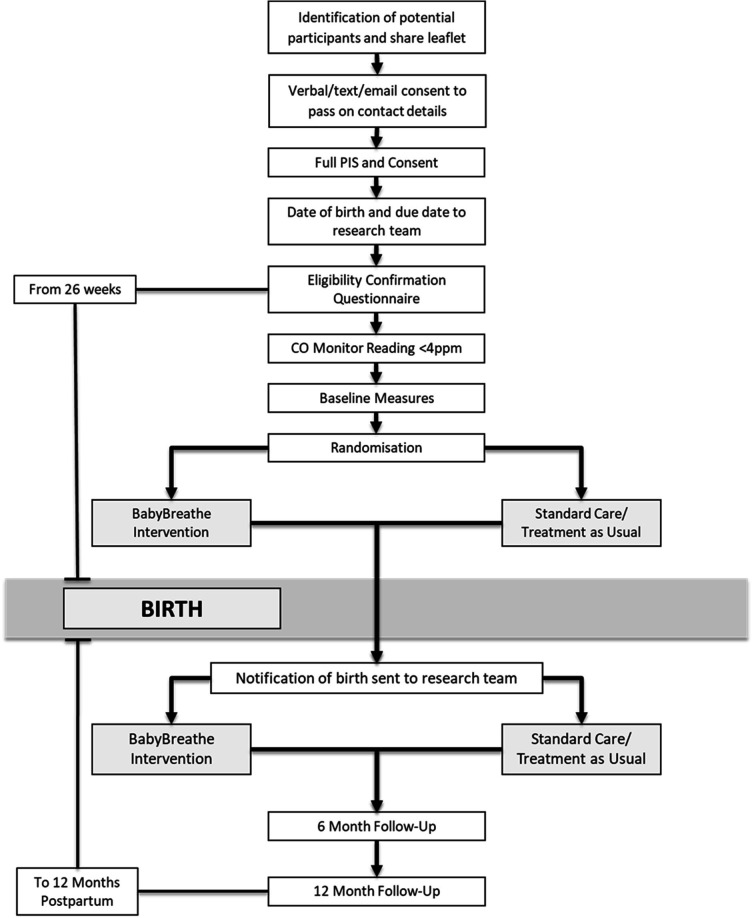
Trial flow diagram. CO, carbon monoxide; PIS, patient information sheet.

Completion and submission of the baseline measures will trigger randomisation (see trial flow diagram, figure 1). Participants will be individually randomised in a 1:1 ratio to the control or intervention groups using a computerised web-based randomisation system managed and accessed only by Norwich Clinical Trials Unit, to ensure adequate sequence generation and allocation sequence concealment. The randomisation system will stratify by recruitment hub, partner smoking status (partner smoking and partner non-smoking/no partner) and time of quit (before or during pregnancy), as these factors are likely to predict relapse.

### Blinding

Blinding is not possible due to the nature of the trial and intervention. The primary outcome is objectively assessed using biochemically validated CO verified smoking abstinence. Therefore, we consider that there is low risk of bias for the primary outcome.

### Internal pilot

The Independent Data Monitoring and Ethics Committee and Independent Trial Steering Committee (TSC) will scrutinise recruitment and protocol fidelity at 6 months into recruitment to establish continuation or stopping the trial at the pilot stage.

### Trial allocation groups

#### Control

Control participants will receive usual antenatal and postnatal care as per the NHS maternity care pathway (ie, no routine relapse prevention support). Usual care varies across the UK and sites have been purposively selected to reflect the variations in routine care. Commonalities in usual care across all the sites include pregnant women being routinely screened for smoking status by their midwife at their first antenatal booking appointment. If a participant reports to be currently smoking, or has a CO reading of 4 ppm or more, they are automatically referred for stop smoking support via NHS commissioned stop smoking services (opt-out referral). Midwifery support focuses on abstinence in pregnancy and only includes brief advice about the opportunity to maintain abstinence postpartum and in the long-term. The usual care group will receive standard ante and postnatal care, as per mandated midwifery and health visitor contacts. These visits may be face-to-face appointments, or they may be remote video or telephone appointments due to the COVID-19 pandemic and local service provision protocols.

#### Intervention

Intervention participants will receive usual care plus the BabyBreathe package of support. The BabyBreathe intervention is informed by the Capability Opportunity Motivation-Behaviour model (COM-B) and Behaviour Change Wheel,^26^ with full consideration of post partum context-specific concerns. The intervention is a package of support designed to be delivered at low cost alongside usual care and existing healthcare services. All intervention components have been developed and initially tested in our preliminary work with pregnant and postpartum people and partners (MRC MR/PO16944/1).^12^ The intervention comprises three main stages:

##### Antenatal support up to birth

- Usual care provision (as delivered in any given site area) will continue also to be provided to the intervention group.
- BabyBreathe relapse prevention leaflet.
- Partner/friend/relative relapse prevention leaflet—content has been designed to encourage partners/friends/relatives to support the participant to stay smoke free after delivery, and to promote active cessation for partners/friends/relatives themselves, where needed.
- Brief advice from a health visitor, health visiting team member or member of the research team trained to deliver the intervention (in-person, or remote). This advice is standardised and scripted following a protocol, with tailored options including positive praise for achieving smoking abstinence and brief advice about the importance of staying smoke-free. Active signposting to the BabyBreathe digital/remote elements are included in this discussion.
- Electronic CO testing—participants are given an iCO monitor (Bedfont) for individual use. Those in the intervention group will be encouraged to use the iCO monitor to self-monitor CO levels at any time during the study (control participants are only prompted to submit a research reading at baseline and study end).
- BabyBreathe website and app—these resources have been specifically developed and the app operates on both Android and iOS (iPhone) operating systems. The website and app can be accessed using a unique code. Users may input details such as the date they quit smoking, their estimated delivery date and may access self-help support, health information and advice, including innovative motivational tools, such as a health calendar and a closed online social support group, in preparation for entering the immediate postpartum period.
- At each subsequent health visitor contact (in-person, or remote), support for maintaining smoking abstinence will be briefly reiterated alongside usual care, where possible.

##### Immediate postnatal period

- BabyBreathe box—once the site team is alerted about the birth and input these data into the REDCap database, the central trial team will post out a BabyBreathe box. This is a physical box, designed to fit through a letter-box. The box includes self-incentive tools (eg, reward chart, journal, photograph frame), free preventative Nicotine Replacement Therapy (NRT, Nicorette Icy White 2 mg, 30 pieces), plus advice and support to use NRT or e-cigarettes for relapse prevention.
- SMS or app notification tailored support—This will be triggered by the birth notification, delivering a programme of tailored relapse prevention messages. Messages start daily, with a diminishing schedule over 12 months. At regular intervals participants are asked to confirm smoking status, and either then stay on the ‘smokefree’ or ‘lapse’ track of tailored messages. There is the option to opt out by texting ‘stop’ at any time.

##### Postnatal period and beyond

- At home/virtual postnatal visit with a health visitor, associated practitioner or BabyBreathe intervention trained researcher at around 10–14 days postpartum, when care is handed over from midwives to health visitors. At this visit, the health visitor will discuss smoking status, give positive praise, offer relapse prevention support, affirm that the BabyBreathe box has been received and discuss contents of the BabyBreathe box, and text/app message use.
- Reiteration of support from health visitors or associated practitioners, up to 12 months postpartum—all subsequent postpartum routine health visitor appointments for the duration of the study will be undertaken by the same health visitor or health visiting team member where possible, to assure continuity of care, which would be anticipated as part of usual practice. Positive praise will be offered for sustained smoking abstinence and the importance of relapse prevention will be emphasised. Participants will be encouraged to continue to engage, or to re-engage, with the full suite of BabyBreathe resources. Cessation support (referral) will be offered to partners where necessary and appropriate. For those who relapse, referral for cessation support will also be offered.

**Figure 2:**
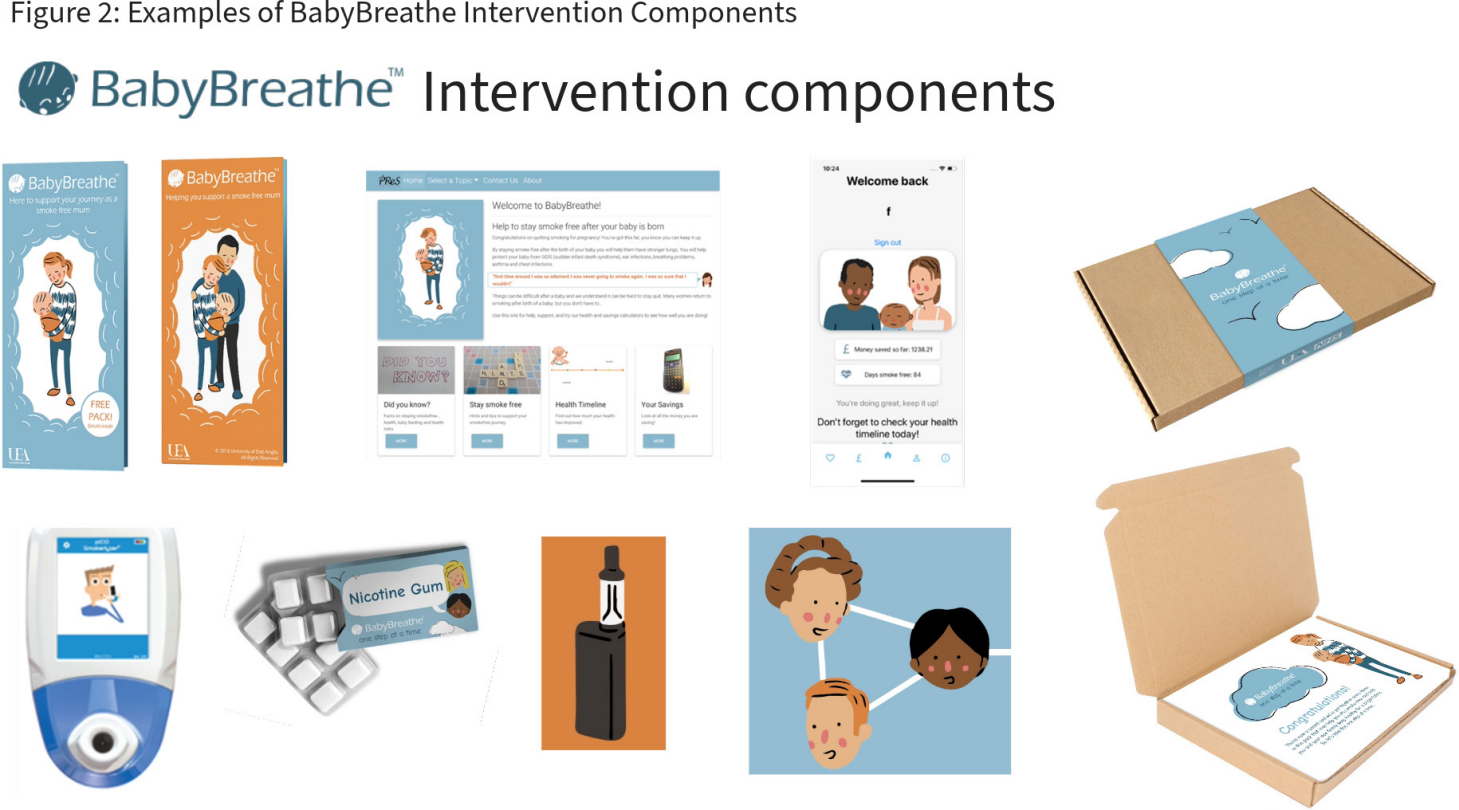
Examples of BabyBreathe intervention components.

See figure 2, for examples, of the components of the BabyBreathe intervention.

### Outcomes

See table 1 for participant timeline of interventions and assessments.

**Table 1:** BabyBreathe participant timeline schedule of enrolment, interventions and assessments

Antenatal				Postnatal				
**Screening** (from 8 weeks to birth)^*^	**Confirm eligibility (from 26 weeks**)	**Baseline (from confirmation of eligibility**)	**Health visit (from randomisation up to birth)**	**Postnatal within 7 days**	**Health visit (10–14 days post partum**)	**Health visits (all subsequent routine**)	**6-month follow-up**	**12-month follow-up**
X								
X								
X								
	X							X
		X						
		X					X	X
		X					X	X
		X					X	X
		X					X	X
		X					X	X
		X					X	X
		X					X	X
		X					X	X
		X					X	X
		X					X	X
		X						
			X	X	X	X	X	X
				X				
								X
								X

*Potential participants will be variously identified during pregnancy: identified at pregnancy booking visit, at dating scans at (8–12 weeks) and/or 20-week scan, by screening records, or at routine care pregnancy appointments occurring anytime between start of pregnancy and 26 weeks of pregnancy, and by self-referral through responding to adverts. These practices will vary and due to pandemic-related restrictions of appointments that may happen remotely. Those missed can be eligible for recruitment following 26 weeks of pregnancy, until the end of pregnancy.

#### Primary outcome

The primary effectiveness outcome is self-reported continuous smoking abstinence, from birth, biochemically validated by CO monitoring at 12 months postpartum, with cut-off of less than 8 ppm (ie, a reading of 7 ppm or less) for those who are not pregnant, or with a cut-off of less than 4 ppm if they are pregnant at this time point, according to the Russell standard.^27^ Adapting the Russell standard for the postpartum population, we will grant a period of ‘grace’, allowing up to five smoking lapses (a one off instance of smoking) between the birth of the baby and the 12-month follow-up, before the outcome is counted as relapse. Participants will provide CO readings electronically, using the iCO monitor (Bedfont) and study app (iCOBabyBreathe) provided at baseline. Participants will provide the REDCap database with two CO readings at entry and follow-up. The highest of the readings will be recorded. Where CO readings take place in person as part of standard care, or research visits, or when participants request help with taking a CO reading, these readings may be used.

#### Secondary outcomes

Secondary outcomes (table 1) measured at 6 and 12 months post partum by online self-report, or researcher follow-up, include self-reported point prevalence abstinence, self-reported time to relapse, participant-reported partner smoking status, self-efficacy (single item, self-report), Edinburgh Postnatal Depression Scale,^27^ behavioural support use (eg, support from a stop smoking service), nicotine product use, perceived stress,^28^ the Alcohol Use Disorders Test for Consumption (AUDIT-C),^29^ health related quality of life (HRQoL) using the EQ-5D-5L.^30^ Infant health outcomes (eg, minor infections requiring General Practitioner (GP) visits and more serious ill health requiring hospitalisation), participant and infant health resource use and cost-effectiveness will be measured at 12 months postpartum using a combination of GP patient records and participant self-report.

### Sample size

If the primary outcome (continued smoking abstinence) occurs in 25% of the control group (based on an estimated relapse rate of 75%^13^) compared with 35% of the intervention group, then we estimate that we will need 880 participants (440 per group) to have 90% power to detect this 10% between group difference at the 5% level of significance. We estimate this difference between control and intervention groups is realistic based on recent trials.^31^ Loss to follow-up or withdrawal is not considered within the sample size calculation, as all those lost to follow-up will be counted as returned to smoking, as is the usual convention in smoking cessation trials.^32^

### Retention

To maximise retention and minimise loss to follow-up, we will make the following efforts to retain contact with study participants. There will be one text/email reminder sent if links to questionnaires/forms are not followed by participants. If participants have not followed the initial links or reminders, then study researchers will contact up to five times to offer support and collect self-report data where possible. Outcome data collection at 6 and 12 months flexibly includes electronic, phone, post and face-to-face options. Participants will also be offered reimbursement for their time (£15 shopping voucher) on completion of 12-month follow-up.

### Data analysis

We will use descriptive statistics to present the baseline characteristics of the two study groups. We will use χ^2^ tests to compare follow-up rates between the study groups, to establish whether there is differential drop out. Analysis of smoking status will be based on the intention-to-treat principle by analysing individuals according to the treatment they were allocated to regardless of compliance. Individuals for whom we do not have the primary outcome data will be assumed to have returned to smoking. Analysis of the primary outcome will be based on a logistic regression model, adjusting for the stratification variables used in the randomisation algorithm. Secondary analysis will adjust for factors known to be predictive of relapse which will be agreed with the TSC and added to the statistical analysis plan (SAP) prior to analysis. Secondary outcomes will be analysed in a similar fashion using a general linear model. Missing data patterns will be examined, and if appropriate, multiple imputation will be undertaken. The SAP is preregistered (on the Open Science Framework (OSF))—see online supplemental appendix 1. The analysis plan may include analysis suggested by the qualitative analysis, such as subgroup analysis or mediation analysis. Any analysis will be prespecified before data lock and published in the SAP prior to any data analysis.

10.1136/bmjopen-2023-076458.supp2 Supplementary data

### Economic evaluation

An economic analysis will be conducted as an integral part of the randomised controlled trial. The primary perspective will be the NHS and social care: however, we will also look at broader relevant costs such as purchase of nicotine replacement therapies. All resources required to provide BabyBreathe will be recorded: these will include staff time; equipment; consumables; required staff training; and any other relevant costs. For staff time to carry out specific tasks to provide BabyBreathe a variety of methods to obtain these data will be explored: these would include trial records on relevant expenditure and expert opinion. Healthcare resource use will be obtained from two sources. First, we will include a modified Client Service Receipt Inventory (CSRI) to obtain data by participant self-report at the 12-month follow-up. This will cover the following: maternal antenatal hospital admissions; details of delivery, including mode of delivery and length of stay; and infant neonatal intensive care unit admissions. Contacts with GP and practice nurses, contact with other primary care practitioners and referral to secondary care will also be collected as well as smoking cessation-related expenditure. Additionally, where feasible we will obtain data from patient notes and GP records. All resources identified during the study will be valued using appropriate local and national unit cost data.

The main outcome measure used in the economic analysis will be the study’s primary outcome measure, continuous postpartum smoking abstinence. This will form a cost-effectiveness study looking at cost per additional sustained abstainer. Additionally, we will use EQ-5D-5L^30^ values obtained from participants to undertake a cost utility analysis (ie, cost per QALY) estimating quality-adjusted life years (QALYs), obtained at baseline, 6 and 12 months postpartum. EQ-5D-5L questionnaires will be valued using the most appropriate scoring algorithm at time of analysis. Currently, this would be the UK mapped scores.^33^ Cost and effectiveness data will be estimated using regression-based methods to allow for differences in baseline characteristics between groups. Non-parametric bootstrapping will be used to allow for uncertainty and this will also be used to construct a cost-effectiveness acceptability curve, which shows how likely the intervention is to be cost-effective at different monetary values of the effectiveness measures. A health economics analysis plan will be agreed and published on the OSF before any analysis of health economics data.

### Process evaluation

Both qualitative and quantitative data will be collected by the study research team to assess implementation of the intervention, mechanisms of impact and contextual influences, as per Medical Research Council guidance^34 35^ (table 2).

**Table 2:** Components of the BabyBreathe mixed-methods process evaluation

Aims	Process evaluation component (Moore *et al*, BMJ 2015)	Method of data collection
Assess fidelity of BabyBreathe training	Implementation. Training.	Questionnaires before and after training
Assess fidelity of intervention contacts	Implementation (intervention contacts). Dose, reach, engagement.	Log of visits by health visitor, health visiting practitioner or researcher (participant level). Audio-recordings of 10% of contacts (antenatal and postnatal). Qualitative interviews (health visitors, members of the health visiting team or researcher—fidelity of delivery). Qualitative interviews (participants and partners—engagement with visits and type of staff delivering the intervention).
Assess fidelity/engagement with the website and application	Implementation (website/application). Dose, reach, engagement.	Website and application data (number of logins, total time in use). Social support group threads. Number of texts received. Discontinuation of text/application notifications. Qualitative interviews (participants).
Assess contamination between trial arms	Implementation (intervention contacts). Contamination.	Recorded by trial research teams at each recruitment hub. Qualitative interviews (health visitors, members of the health visiting team or researchers). Health visitor feedback groups.
Assess protocol modifications	Implementation (intervention contacts, website/application). Fidelity, adaptations (intended and unintended/unforeseen; positive adaptations or drift).	Recorded by trial research teams at each recruitment hub. Qualitative interviews (health visitors, members of the health visiting team or researchers). Health visitor, member of the health visiting team and researcher feedback groups.
Assess how the intervention worked	Mechanisms of impact: hypothesised and unintended/unexpected pathways.	Engagement data across recruitment hubs (visits). Engagement with website and application. Engagement with text support. Use of BabyBreathe box components (self-report, qualitative interviews and health visitor interviews). Qualitative interviews (participants).
Assess contextual influences on implementation and mechanisms of impact	Context: contextual influences, eg, participant/health visitor characteristics and geographical region, on implementation and mechanisms of impact.	Qualitative interviews with health visitors, members of the health visiting team, or researchers and participants.
Assess the impact of the COVID-19 pandemic on intervention delivery and participant efforts to remain quit/stop smoking (partner)	Implementation processes (health visitor perspective). Fidelity. Adaptions (by health visitors, members of the health visiting team or researchers). Context. COVID-19 pandemic response, eg, restrictions, (partial) lockdowns. Mechanisms of impact. Mediators.	Qualitative interviews with health visitors, members of the health visiting team, or researchers and participants.

Fidelity of intervention delivery (*implementation*) and participant engagement with the health visitor visits and website/app will be assessed quantitatively through logs of visits, data analytics for website/app usage (the number of times that systems are logged on to, which resources are accessed, the time of engagement, the delivery of support messages via notifications and text messages, the time of any disengagement, discontinuation of SMS or app notifications and self-reported engagement (as per^36^). Qualitative analysis will be undertaken of social support group threads, for which consent will have been sought on recruitment to the study; and audio-recordings (health visitors, practitioners or BabyBreathe researchers will be asked to record approximately 10% of visits (≤10 min intervention only), antenatal as well as postnatal) and interviews with health visitors (n=12) and a qualitative interview subsample of participants and partners (n=40). Potential contamination between trial arms and protocol modifications will be assessed through qualitative interviews with health visitors and regular reporting by trial research teams. We will assess whether any identifiable modifications were planned adaptations to fit context, or unforeseen, and report our findings according to FRAME, an established framework.^37^ To illuminate possible mechanisms of action, a combined analysis of qualitative participant interview data, audio-recordings (eg, intervention duration, delivery of behaviour change techniques) and quantitative engagement data across recruitment hubs will assess which components of the intervention were perceived to be particularly effective, for which people, in which contexts.

### Data management

In view of the nature of the population (who are all expected to have one or more pregnancy-related hospitalisation and primary care attendances which will be recorded in medical records); the intervention (which is not a medicinal product with the exception of nicotine replacement therapy (gum) included in the BabyBreathe box; and the trial primary and secondary outcomes, we do not intend to collect any additional safety endpoints.

BabyBreathe trial team members review the trial database to generate reports and review data entry. The essential trial issues, events and outputs, including defined key data points, are discussed by the trial team on a weekly basis and with relevant committees when necessary. A data sharing statement is included in the trial registry entry.

### Ethics and dissemination

Full research ethics committee (REC) and Health Research Authority (HRA) approval has been granted (REC reference: 21/NW/0017, IRAS Project ID: 291746, protocol V.7 dated 04 May 2022). Participants provide electronic consent to take part, and rights of refusal to participate, or requests of withdrawal will be respected.

The results of the trial will be disseminated in open access journals, regardless of the direction of effect. The full protocol, statistical analysis plan, qualitative and health economics analysis plans and anonymised data sets will be published in an online open access repository.

### Current study status

Recruitment opened in April 2021 and the first participant was randomised in September 2021. Recruitment is expected to take 24 months, with results expected to be published following final follow-up in late 2024 or early 2025.

## Supplementary Material

Reviewer comments

Author's manuscript
