# Supplementary material for: BabyBreathe trial: protocol for a randomised controlled trial of a complex intervention to prevent postpartum return to smoking
Source: BMJ Open. 2023 Sep 4;13(9):e076458. doi: 10.1136/bmjopen-2023-076458 (PMC10481735; doi:10.1136/bmjopen-2023-076458)
Supplement: Supplementary data [file bmjopen-2023-076458supp002.pdf]

NORWICH CTU

Babybreathe

A RANDOMISED CONTROLLED TRIAL OF A COMPLEX INTERVENTION TO PREVENT RETURN TO SMOKING IN WOMEN POSTPARTUM

Statistical Analysis Plan (SAP)

Version 1.0

04.06.2023

Name	Title	Signature	Date
Caitlin Notley	Chief Investigator	[signature]	04.06.2023
Allan Clark	Statistician	[signature]	07.6.2023
Lucy Clark	Trial Manager	[signature]	07.06.2023

SAP REVISION HISTORY

Document Name	Version No.	Reason for Revision	Effective Date

Notley C, *et al. BMJ Open* 2023; 13:e076458. doi: 10.1136/bmjopen-2023-076458

1.0 Administrative Information

Sponsor : Norfolk and Norwich University Hospitals NHS Foundation Trust (NNUH)

Sponsor Reference : R207276

Funder : National Institute for Health Research – Health Technology Assessment

Funder Reference : NIHR129074

Trial Registration : ISRCTN70307341

IRAS: 291746

Chief Investigator : Caitlin Notley

Trial Statistician : Allan Clark

UKCRC Trials Unit : NCTU

Latest Protocol : Version 7.0

Notley C, *et al. BMJ Open* 2023; 13:e076458. doi: 10.1136/bmjopen-2023-076458

2.0 Introduction

2.1 Background and Rationale
This is provided in section 4.1 of the protocol.

2.2 Objectives
The overall trial objectives are provided in section 4.2 of the protocol, however this SAP covers the following

1. To run an internal pilot study, with clear stop/go trial embedded criteria, primarily to test recruitment systems.

2. To definitively test the real-world effectiveness of BabyBreathe in comparison with usual care, by comparing smoking abstinence rates at 12 months postpartum between trial groups.

3.0 Study Methods

3.1 Trial Design
A two-group, multi-centre, pragmatic, individually randomised, controlled trial with an internal pilot, and including economic evaluation and process evaluation.

Intervention: Intervention participants will receive usual care plus the BabyBreathe package of support. The BabyBreathe intervention is informed by the Capability Opportunity Motivation-Behaviour (COM-B) model and Behaviour Change Wheel (21), with full consideration of postpartum women's context-specific concerns. The intervention is a package of support designed to be delivered at low cost alongside usual care and existing healthcare services. All intervention components have been developed and initially tested in our preliminary work with women and partners (MRC MR/PO16944/1) (9). The intervention comprises three main stages:

1. Antenatal support up to birth:

A.) BabyBreathe™ relapse prevention leaflet.
B.) Partner/Friend/Relative relapse prevention leaflet – content has been designed to encourage partners/friends/relatives to support women to stay smoke free after delivery, and to promote active cessation for partners/friends/relatives themselves, where needed.
C.) Brief advice from a health visitor, heath visiting team member practitioner or member of the research team trained to deliver the intervention (in-person, or remote). This advice is standardised and scripted following a protocol, with tailored options including positive praise for achieving smoking abstinence, and brief advice about the importance of staying smoke-free. Active signposting to the BabyBreathe digital /remote elements are included in this discussion.
D.) Electronic carbon monoxide testing – women are given an iCO monitor (Bedfont) for individual use. Intervention women will be prompted to use the iCO monitor to self-monitor CO levels at any time during the study (control participants will be restricted to use at baseline and study end).

NORWICH CTU

E.) BabyBreathe website and app – these resources have been specifically developed and operate on both android and iOS (iPhone) systems. The website and app can be accessed using a unique code by women for free. Women may input details such as the date they quit smoking, their estimated delivery date (EDD), and may access self-help support, health information and advice, including innovative motivational tools, such as a health calendar, and a closed online social support group, in preparation for entering the immediate postpartum period.

F.) At each subsequent health visitor contact (in-person, or remote), support for maintaining smoking abstinence will be briefly reiterated alongside usual care, where possible.

G.) Usual care provision (as delivered in any given site area) will continue also to be provided to the intervention group.

2. Immediate postnatal period

H.) BabyBreathe box – once the site team are alerted about the birth and input these data into the REDCap database, the central trial team will post out a BabyBreathe box. This is a physical box, designed to fit through a letter-box. The box includes self-incentives (e.g. reward chart, journal, photograph frame), free preventative NRT (Nicorette Icy White 2mg), plus advice and support to use NRT or e-cigarettes for relapse prevention.

I.) SMS or app notification tailored support – This will be triggered by the birth notification, delivering a programme of tailored relapse prevention messages that draw on data initially inputted by the user.

3. Postnatal period and beyond

J.) At home/virtual postnatal visit with a health visitor, associated practitioner or BabyBreathe intervention trained researcher at around 10-14 days postpartum, when full? care of women and babies is handed over from midwives to health visitors. At this visit, the health visitor will discuss smoking status, give positive praise, offer relapse prevention support, affirm that the BabyBreathe box has been received, and discuss contents of the BabyBreathe box, and text/app message use.

K.) Reiteration of support from health visitors or associated practitioners, up to 12 months postpartum – all subsequent postpartum routine health visitor appointments for the duration of the study will be undertaken by the same health visitor, health visiting team member, to assure continuity of care, which would be anticipated as part of usual practice. Positive praise will be offered for sustained smoking abstinence and the importance of relapse prevention will be emphasised. Women will be encouraged to continue to engage, or to re-engage, with the full suite of BabyBreathe resources. Cessation support (referral) will be offered to partners where necessary and appropriate. For women who relapse, referral for cessation support will also be offered.

Control:

Control participants will receive usual antenatal and postnatal care as per the NHS maternity care pathway (i.e. no routine relapse prevention support). Usual care varies across the UK and sites have been purposively selected to reflect the variations in routine care. Commonalities in usual care across all the sites include pregnant women being routinely screened for smoking status by their midwife at their

Notley C, *et al. BMJ Open* 2023; 13:e076458. doi: 10.1136/bmjopen-2023-076458

first antenatal booking appointment. If a woman reports that she is currently smoking, or she has a CO reading greater than 3ppm (i.e. a reading of 4ppm or more), she is automatically referred for stop smoking support via NHS commissioned stop smoking services (opt-out referral). Midwifery support focuses on abstinence in pregnancy and only includes brief advice about the opportunity to maintain abstinence postpartum and in the long-term. The usual care group will receive standard ante and postnatal care, as per mandated midwifery and health visitor contacts. These visits may be face-to-face appointments, or they may be remote video or telephone appointments due to the COVID-19 pandemic.

3.2 Allocation

Completion and submission of the baseline measures will trigger randomisation (see trial flow diagram, figure 1). Participants will be individually randomised 1:1 via a computerised randomisation system managed by Norwich Clinical Trials Unit, to ensure adequate sequence generation and allocation sequence concealment. The randomisation system will stratify by recruitment hub, partner smoking status (partner smoking and partner non-smoking/no partner) and time of quit (before or during pregnancy), as factors that are likely to predict relapse.

3.3 Sample Size

This is provided in section 5.8 of the protocol but is repeated below.

If the primary outcome (continued smoking abstinence) occurs in 25% of the control group (based on an estimated relapse rate of 75% (10)) compared with 35% of the intervention group, then we estimate that we will need 880 participants (440 per group, 220 per recruitment hub) to have 90% power to detect this 10% between group difference at the 5% level of significance. We estimate this difference between control and intervention groups is realistic based on recent trials (27). Loss to follow up or withdrawal is not considered within the sample size calculation, as all those lost to follow up will be counted as returned to smoking, as is the usual convention in smoking cessation trials (ref). From national data, we estimate that approximately 22% of women will be smoking in the 12 months prior to pregnancy.

3.4 Framework

This is a superiority framework comparing the intervention to the control.

Notley C, *et al. BMJ Open* 2023; 13:e076458. doi: 10.1136/bmjopen-2023-076458

3.5 Timing of outcome assessments

Table 1: BABYBREATHE PARTICIPANT TIMELINE

Schedule of enrolment, interventions, and assessments.

	**Antenatal**					**Postnatal**				
	**Screening (From 8 weeks to birth)***	**Confirm Eligibility (From 26 weeks)**	**Baseline (From confirmation of eligibility)**	**Health Visit (From randomisation up to birth)**	Birth	**Postnatal within 7 days**	**Health Visit (10-14 days Postpartum)**	**Health Visits (All subsequent routine)**	**6 month follow up**	**12 month follow up**
Eligibility	X									
Consent to be contacted	X									
Link to PIS and Consent	X									
iCO reading to confirm eligibility		X								X
Demographics			X							
Smoking Status (SR)			X						X	X
Breastfeeding intention (ref)			X						X	X
Relapse Predictors			X						X	X
Self-Efficacy (SR)			X						X	X
Edinburgh Depression Scale (22)			X						X	X
Behavioural Support (SR)			X						X	X
Nicotine Product Use (SR)			X						X	X
AUDIT-C (23)			X						X	X
EQ-5D-5L (45)			X						X	X
Cohen 4 item Perceived stress scale (24)			X						X	X
Randomisation			X							
BabyBreathe Intervention				X		X	X	X	X	X
Birth Notification						X				
Healthcare Resource Use										X
Infant Health Outcomes										X

Notley C, *et al. BMJ Open* 2023; 13:e076458. doi: 10.1136/bmjopen-2023-076458

NORWICH CTU

*Potential participants will be variously identified during pregnancy: identified at pregnancy booking visit, at dating scans at (8-12 weeks) and/or 20 week scan, by screening records, or at routine care pregnancy appointments occurring anytime between start of pregnancy and 26 weeks of pregnancy, and by self-referral through responding to adverts. These practices will vary and due to pandemic related restrictions of appointments that may happen remotely. Those missed can be eligible for recruitment following 26 weeks of pregnancy, until the end of pregnancy

3.6 Interim analyses and stopping guidance

There will be no formal interim analyses or stopping guidance. However, the trial does have progression criteria the end of the internal pilot stage after three months recruitment. These are listed below.

3.7 Timing of analyses

The internal pilot does not require the analysis of any outcomes or unblinded data so is not considered as 'analysis' for this SAP. The analysis will be done once the database is locked and the SAP approved once all of the outcome data has been collected.

4.0 Statistical Principles

4.1 Levels of statistical significance

A 5% level of significance and 95% confidence intervals will be used throughout.

4.2 Analysis populations

The ITT population is defined as the set of all randomized participants regardless of compliance. If participants are subsequently withdrawn from the study then there data will still be included and the missing data strategy detailed in the analysis section will be used. Individuals who are deemed to be post-randomisation exclusions will be excluded from the analysis.

A modified ITT population will exclude participants who had complications at birth. Analysis of this population will be made on the primary outcome only.

4.3 Treatment Adherence / received

Compliance of the intervention along with the treatment received will be reported as per the table below.

Norwich CTU

Table 4.3.1: Compliance / treatment received

	Intervention delivery
**Antenatal support period**	n (%)
Relapse prevention leaflet.	
Partner/Friend/Relative relapse prevention leaflet	
Brief advice from a health visitor	
Electronic carbon monoxide testing given	
BabyBreathe website and app provided / accessed	
**Immediate postnatal period**	
BabyBreathe box sent	
SMS or app notification sent	
SMS or app opt out received	
**Postnatal period and beyond**	
At home/virtual postnatal visit with a health visitor	
Reiteration of support from health visitors	
Number of postpartum visits	
None	
One	
Two	
Three	
Four	
…	

4.4 Protocol deviations

Protocol deviations will be discussed at the TMG and will be reported as a list.

5.0 Trial Population

5.1 Screening data

The following data and tables will be reported from the screening data.

Table 5.1.1: Screening data by month of approaching patient

Month of screening	Number of patients approached	Number interested in participating	Number eligible* (pre-screening)	Number giving consent	Number eligible **	Number randomised

Table 5.1.2: Reasons for declining

	Number (% Of those declining participation)	Percentage of those approached

5.2 Eligibility

5.2.1 Participant Inclusion Criteria

1. Those who are pregnant who have stopped smoking completely in the 12 months prior to pregnancy, or at any time during pregnancy.
2. At 26 weeks gestation or any time following this up until birth, woman confirms having not smoked a single puff of a cigarette for at least four weeks.
3. Able to read and understand English.
4. Willing and able to give informed consent for participation in the study.
5. Expired carbon monoxide (CO) reading less than four parts per million (ppm)

5.2.2 Participant Exclusion Criteria

6. Under the age of 16

This will be reported as below.

Table 5.2.1: Reasons for ineligibility

	Frequency (%) (N=)
Pre-screening eligibility criteria met	
**Reason for exclusion (n=)**<br>Absences of inclusion criteria	
Those who are pregnantwho have stopped smoking completely in the 12 months prior to pregnancy, or at any time during pregnancy.	
At 26 weeks gestation or any time following this up until birth, participant confirms having not smoked a single puff of a cigarette for at least four weeks.	
Able to read and understand English.	

Notley C, *et al. BMJ Open* 2023; 13:e076458. doi: 10.1136/bmjopen-2023-076458

Willing and able to give informed consent for participation in the study	
Expired carbon monoxide (CO) reading less than four parts per million (ppm)	
**Presence of exclusion criteria**	
Aged under 16	

5.3 Recruitment and participant flow

Table 5.3.1: Participant accrual (e.g. per time period, cumulative, if appropriate against predicted accrual in graphical form) for main participants (ITT population only)

Month of recruitment	Predicted	Actual	Cumulative Predicted	Cumulative Actual

Graph of predicted vs actual recruitment

A CONSORT diagram will also be produced.

5.4 Withdrawal information

Follow-up rates and reasons for withdrawal will be reported in the following tables.

Table 5.4.1: Follow-up

	ITT population	
	Control (n=)	Intervention (n=)
Lost to FU before birth		
Lost to FU month 0-6 post partum, n(%)		
Lost to FU month 7-12 post partum, n(%)		

Table 5.4.2: Reasons for loss to follow-up.

	ITT population		ITT+ population	
	Control (n=)	Intervention (n=)	Control (n=)	Intervention (n=)
Reason lost to follow (month 0-6)				
Reason 1, n(%)				
Reason 2, n(%)				
….				
Reason lost to follow (month 6-12)				
Reason 1, n(%)				
Reason 2, n(%)				
….				

5.5 Baseline participant characteristics

The baseline characteristics will be summarized according to the table below.

Table 8: Baseline characteristics of trial participants

	ITT population (n=)	
	Control (n=)	Intervention (n=)
Age, mean (SD)		
Number of days into pregnancy when recruited, mean (SD)		
Number of days until due date, mean (SD)		
Days since last puff, mean (SD)		
When did you quit smoking		
Before pregnancy, n(%)		
During pregnancy, n(%)		
Partner smoking status		
No partner, n(%)		
Smoker, n(%)		
Never smoker, n(%)		
Ex smoker, n(%)		
Highest qualification		
None, n(%)		
GCSE, n(%)		
A-level, n(%)		
Degree, n(%)		

Notley C, *et al. BMJ Open* 2023; 13:e076458. doi: 10.1136/bmjopen-2023-076458

Missing, n(%)		
Ethnicity		
White, n(%)		
Mixed, n(%)		
Asian / Asian British, n(%)		
Black/African/Caribbean/Black British, n(%)		
Arab, n(%)		
Any other ethnic group, n(%)		
Missing, n(%)		
Marital status		
Single, n(%)		
Co-habiting, n(%)		
Civil partnership, n(%)		
Married, n(%)		
Divorced, n(%)		
Widowed, n(%)		
Missing, n(%)		
Confidence not continue to smoke until baby's first birthday		
Not at all confident		
Slightly confident		
Moderately confident		
Very confident		
Extremely confident		
Use of Nicotine replacement therapy		
Have you used any Nicotine Replacement Therapy (NRT) in the last week?		
Did you use an e-cigarette to help you stop smoking?		
have you used an e-cigarette in the last week?		
Did you use a heat-not-burn product to help you stopsmoking?		
Have you used a heat-not burn product in the last week?		
Did you receive any professional help with stopping smoking?		
Do you still receive help from this organisation to stay smoke free?		
Are you currently using any apps which help with quittingsmoking or staying quit from smoking?		
Edinburgh post natal depression scale, mean (SD)		
PSS4 score		

6.0 Analysis

NORWICH CTU

6.1 Outcome definitions

6.1.1 Primary Outcome

The primary effectiveness outcome is self-reported continuous postpartum smoking abstinence, biochemically validated by CO monitoring at 12 months postpartum, with cut off of less than 8ppm (i.e. a reading of 7ppm or less) for women who are not pregnant, or with a cut off of less than 4ppm if they are pregnant at this time point, according to the Russell standard (25,26). Adapting the Russell standard, we will grant a period of 'grace', allowing up to 5 smoking lapses between the birth of the baby and the 12 month follow-up, before the outcome is counted as relapse. Participants will provide CO readings electronically, using the iCO monitor (Bedfont) and study app (iCOBabyBreathe) provided at baseline. Participants will provide the REDCap database with two CO readings. Where CO readings take place in person as part of standard care, or research visits, these readings may be used.

This will be constructed from

- the question "Are you currently smoke free?" taking smoke free to be either
  - "Abstinent" with the answers " Yes I am smoke free - I have not smoked a cigarette in the last 12 months (not even a puff of a cigarette)" or " Yes I am smoke free currently but I have had between one and five lapses in the last 12 months (a cigarette, or puff of a cigarette)"; and
  - "relapse" to be either "Yes I am currently smoke free but I have had six or more lapses in the last 12 months (including relapse but quit again) " or "No, I am currently smoking tobacco"; and
- The CO readings will be classified as 'verified' if a reading of 7ppm or less; and 'not verified' if 8 or more. Missing values will be classified as 'not verified'

The primary outcome with be classified as "confirmed abstinent" if the participant's response is both "Abstinent" and "verified"; otherwise it will be classified "Not conformed abstinent".

6.1.2 Secondary Outcomes

The secondary outcomes are measured at 6 and 12 months postpartum and are:

- Self-reported abstinence defined as reporting less than 5 lapses in the last 6 months at 6 months and at 12 months. [1 or 2 in fu6_smoking_status] [1 or 2 in fu12_smoking_status]
- Self-reported time to relapse defined as time from birth of child until individual self-reported date when started smoking again [fu_smoking_again_de] for individuals who report that they are currently smoking at either 6 or 12 months.
- Relapse predictors
  - Partner smoking status [fu_partner_smoke_yn]
  - Self-reported breast feeding at 6 and 12 months (yes/no)
  - Self-reported duration of breast feeding

NORWICH CTU

- Self-efficacy to remain smoke free measured using the question 'How confident are you that you will continue not to smoke at least until your baby's first birthday?' at 6 months and 'How confident are you that you will continue not to smoke?'
- Postnatal depression measured the Edinburgh postnatal depression scale. This has 10 items and is scored using a scale ranging from 0 to 30 with high value indicating greater chance of depression. The scoring guide used will be
- Behavioral support use measured using the question 'In the last 6 months have you received any professional help with stopping smoking, e.g. from NHS smokefree services?' This will be analysed separately for
    - Smoking-free services
    - GP advice
    - Digital smokefree services
- Nicotine product use measure as use of Nicotine Replacement Therapy in the last 6 months.
- Nicotine product use measure as use of Nicotine Replacement Therapy in the last 7 days
- Perceived stress will be measured using Cohen PSS4 scale. This consists 4 items each scored 0-4, with the total score ranging from 0 to 16 with higher values indicates more stress.
- AUDIT-C this is a questionnaire based on 3 questions [alcohol_frequency_fu], [alcohol_units_fu] and [alcohol_6ormore_units] each scored 0-4 and the total is scored 0-12 with higher values indicating higher chance of possible dependence.
- EQ-5D-5L
- Infant health outcomes measured by
    - Number of hospital admissions
    - Number of GP visits
    - Length of stay when giving birth
    - Neonatal unit admission or not.
- E-cig use
    - In last 6 months
    - Frequency of use in last 6 months
    - In last 7 days

6.1.3 Tertiary outcomes
None.

6.2 Analysis Methods

6.2.1 Primary outcome

The primary outcome will be compared between treatment groups using a log-binomial regression adjusting for the stratification variables in a 'minimally adjusted' model; if adjustment for additional variables is recommended from the TSC prior to analysis this will be detailed in this document. This will allow the estimation of the relative risk of abstinence between the two treatment groups. The risk difference will be estimated from this model using the predicted risk, those factors in the model which are categorical will be set at the value with the largest number of participants and the continuous

Notley C, *et al. BMJ Open* 2023; 13:e076458. doi: 10.1136/bmjopen-2023-076458

factors will be set at the mean value. Any individual with missing data will be assumed to have relapsed, in the event of the abstinence not being able to be confirmed biochemically it will also be assumed to have been in relapse.

Table 6.2.1: Summary for primary outcome (will be reported for the ITT and ITT+ populations)

	ITT population		Minimally adjusted (only for stratification variables)			Fully adjusted		
Outcome	Control (n=)	Intervention (n=)	Relative risk (95% CI)	p-value	Difference in risk (95%CI)	Relative risk (95% CI)	p-value	Difference in risk (95%CI)
12 Month abstinence	n(%)	n(%)						

6.2.2 Secondary outcomes

The following tables give the analysis for each outcome listed in section 6.1.2

Outcome	Self-reported continuous postpartum smoking abstinence. CO verified
Effect size	Relative risk
Primary Analysis model	Log-binomial regression adjusting for factors stratified in the randomisation.
Sensitivity analysis	Log-binomial regression adjusting for factors stratified in the randomisation and factors pre-specified by TMG before database lock.
Missing data	Missing data will be assumed to have relapsed into smoking.
Other comments	The effect size will also be estimated as the risk difference and 'number-need-to-treat' using an unadjusted model.<br>Biochemical validation of self-reported abstinence is the gold standard outcome assessment in smoking cessation/relapse prevention trials (27).

Outcome	6 month Self-reported continuous postpartum smoking abstinence
Effect size	Relative risk
Primary Analysis model	Log-binomial regression adjusting for factors stratified in the randomisation.

Notley C, *et al. BMJ Open* 2023; 13:e076458. doi: 10.1136/bmjopen-2023-076458

NORWICH CTU

Sensitivity analysis	Log-binomial regression adjusting for factors stratified in the randomisation and factors pre-specified by TMG before database lock.
Missing data	Missing data will be assumed to have relapsed into smoking.
Other comments	The effect size will also be estimated as the risk difference and 'number-need-to-treat' using an unadjusted model.

Outcome	Self-reported time-to-relapse, defined as the time from randomisation until the date first smoked
Effect size	Hazard ratio
Primary Analysis model	Cox regression adjusting for factors stratified in the randomisation.
Sensitivity analysis	Cox regression adjusting for factors stratified in the randomisation and factors pre-specified by TMG before database lock.
Missing data	Individuals who drop-out will be assumed to have relapsed on the date of drop-out.<br>Individuals who have not relapsed will be censored at the end of the study.
Other comments	Data will be presented graphically using a Kaplan-Meier Curve.<br>Cox regression assumptions will be assessed visually using a plot of Schoenfeld residuals against follow-up time.<br>If assumptions not met then other models adjustments to the model (treating variables as strata rather than covariates) will be attempted. If not possible then alternative modelling will be investigated.

Outcome	Participant reported partner smoking status at 6 and 12 months
Effect size	Relative risk
Primary Analysis model	Log-binomial regression adjusting for factors stratified in the randomisation.
Sensitivity analysis	Log-binomial regression adjusting for factors stratified in the randomisation and factors pre-specified by TMG before database lock.
Missing data	Missing data will be assumed to have relapsed into smoking.

Notley C, *et al. BMJ Open* 2023; 13:e076458. doi: 10.1136/bmjopen-2023-076458

Other comments	Only to be analysed for individuals with a) the same partner status as at baseline; and b) the partner had quit by the date of randomisation

Outcome	Self-reported breastfeeding status
Effect size	Relative risk
Primary Analysis model	Log-binomial regression adjusting for factors stratified in the randomisation.
Sensitivity analysis	Log-binomial regression adjusting for factors stratified in the randomisation and factors pre-specified by TMG before database lock.
Missing data	Missing data will be imputed in a sensitivity analysis.
Other comments	Binary yes/no at 12 months

Outcome	Self-reported duration of breastfeeding (duration)
Effect size	Mean difference
Primary Analysis model	General linear model adjusting for factors stratified in the randomisation.
Sensitivity analysis	General linear model adjusting for factors stratified in the randomisation and factors pre-specified by TMG before database lock.
Missing data	Missing data will be imputed in a sensitivity analysis.
Other comments	

Outcome	Self-efficacy to remain smokefree
Effect size	Relative risk
Primary Analysis model	Log-binomial regression adjusting for factors stratified in the randomisation.
Sensitivity analysis	Log-binomial regression adjusting for factors stratified in the randomisation and factors pre-specified by TMG before database lock.
Missing data	Missing data will be imputed in a sensitivity analysis.
Other comments	Binary yes/no at 12 months

Outcome	Postpartum depression – Edinburgh postnatal depression questionnaire (39)
Effect size	Mean difference

Notley C, *et al. BMJ Open* 2023; 13:e076458. doi: 10.1136/bmjopen-2023-076458

NORWICH CTU

Primary Analysis model	Linear regression adjusting for factors stratified in the randomisation.
Sensitivity analysis	Linear regression adjusting for factors stratified in the randomisation and factors pre-specified by TMG before database lock.
Missing data	Missing data will be assumed to have relapsed into smoking.
Other comments	Assumptions will be assessed via plots of residuals to check for normality. If not normally distributed a non-parametric bootstrap will be used or a Mann-Whitney test will be used.

Outcome	Access in-person smoke free services
Effect size	Relative risk
Primary Analysis model	Log-binomial regression adjusting for factors stratified in the randomisation.
Sensitivity analysis	Log-binomial regression adjusting for factors stratified in the randomisation and factors pre-specified by TMG before database lock.
Missing data	Missing data will be imputed in a sensitivity analysis.
Other comments	Binary yes/no at 12 months

Outcome	Access in-person GP advice for stop smoking
Effect size	Relative risk
Primary Analysis model	Log-binomial regression adjusting for factors stratified in the randomisation.
Sensitivity analysis	Log-binomial regression adjusting for factors stratified in the randomisation and factors pre-specified by TMG before database lock.
Missing data	Missing data will be imputed in a sensitivity analysis.
Other comments	Binary yes/no at 12 months

Outcome	Access digital smoke free services
Effect size	Relative risk
Primary Analysis model	Log-binomial regression adjusting for factors stratified in the randomisation.
Sensitivity analysis	Log-binomial regression adjusting for factors stratified in the randomisation and factors pre-specified by TMG before database lock.

Notley C, *et al. BMJ Open* 2023; 13:e076458. doi: 10.1136/bmjopen-2023-076458

NORWICH CTU

Missing data	Missing data will be imputed in a sensitivity analysis.
Other comments	Binary yes/no at 12 months

Outcome	Nicotine product use in last 6 months
Effect size	Relative risk
Primary Analysis model	Log-binomial regression adjusting for factors stratified in the randomisation.
Sensitivity analysis	Log-binomial regression adjusting for factors stratified in the randomisation and factors pre-specified by TMG before database lock.
Missing data	Missing data will be imputed in a sensitivity analysis.
Other comments	Binary yes/no at 12 months

Outcome	Nicotine product use in last 7 days
Effect size	Relative risk
Primary Analysis model	Log-binomial regression adjusting for factors stratified in the randomisation.
Sensitivity analysis	Log-binomial regression adjusting for factors stratified in the randomisation and factors pre-specified by TMG before database lock.
Missing data	Missing data will be imputed in a sensitivity analysis.
Other comments	Binary yes/no at 12 months

Outcome	Perceived Stress – Cohen perceived stress scale (41)
Effect size	Mean difference
Primary Analysis model	Linear regression adjusting for factors stratified in the randomisation.
Sensitivity analysis	Linear regression adjusting for factors stratified in the randomisation and factors pre-specified by TMG before database lock.
Missing data	Missing data will be assumed to have relapsed into smoking.
Other comments	Assumptions will be assessed via plots of residuals to check for normality. If not normally

NORWICH CTU

	distributed a non-parametric bootstrap will be used or a Mann-Whitney test will be used.

Outcome	Alcohol Use (AUDIT-C) (40)
Effect size	None.
Primary Analysis model	Mann-Whitney test.
Sensitivity analysis	None.
Missing data	Missing data will be imputed in a sensitivity analysis.
Other comments	This outcome is unlikely to be normally distributed so a non-parametric approach will be used.

Outcome	Quality of life EQ-5D-5L
Effect size	Mean difference
Primary Analysis model	Linear regression adjusting for factors stratified in the randomisation.
Sensitivity analysis	Linear regression adjusting for factors stratified in the randomisation and factors pre-specified by TMG before database lock.
Missing data	Missing data will be assumed to have relapsed into smoking.
Other comments	Assumptions will be assess via plots of residuals to check for normality. If not normally distributed a non-parametric bootstrap will be used

Outcome	Number of hospital admission for child in follow-up period
Effect size	Incident rate ratio
Primary Analysis model	Poisson regression adjusting for factors stratified in the randomisation.
Sensitivity analysis	Poisson regression adjusting for factors stratified in the randomisation and factors pre-specified by TMG before database lock.
Missing data	Missing data will be assumed to have relapsed into smoking.
Other comments	Assumptions will be assessed and if a negative binomial model fits the data better then it will be used.

Outcome	Number of GP visits of child in follow-up period
Effect size	Incident rate ratio
Primary Analysis model	Poisson regression adjusting for factors stratified in the randomisation.
Sensitivity analysis	Poisson regression adjusting for factors stratified in the randomisation and factors pre-specified by TMG before database lock.
Missing data	Missing data will be assumed to have relapsed into smoking.
Other comments	Assumptions will be assessed and if a negative binomial model fits the data better then it will be used.

Outcome	Length of stay on birth
Effect size	Mean difference
Primary Analysis model	Linear regression adjusting for factors stratified in the randomisation.
Sensitivity analysis	Linear regression adjusting for factors stratified in the randomisation and factors pre-specified by TMG before database lock.
Missing data	Missing data will be assumed to have relapsed into smoking.
Other comments	Assumptions will be assessed via plots of residuals to check for normality. If not normally distributed a non-parametric bootstrap will be used

Outcome	Neonatal unit admission of child
Effect size	Relative risk
Primary Analysis model	Log-binomial regression adjusting for factors stratified in the randomisation.
Sensitivity analysis	Log-binomial regression adjusting for factors stratified in the randomisation and factors pre-specified by TMG before database lock.
Missing data	Missing data will be imputed in a sensitivity analysis.
Other comments	

Outcome	E-cigarette use in 6 months
Effect size	Relative risk
Primary Analysis model	Log-binomial regression adjusting for factors stratified in the randomisation.

Notley C, *et al. BMJ Open* 2023; 13:e076458. doi: 10.1136/bmjopen-2023-076458

Sensitivity analysis	Log-binomial regression adjusting for factors stratified in the randomisation and factors pre-specified by TMG before database lock.
Missing data	Missing data will be imputed in a sensitivity analysis.
Other comments	

Outcome	Frequency of using an 3-cigarette in past 6 months
Effect size	None.
Primary Analysis model	Mann-Whitney test.
Sensitivity analysis	None.
Missing data	Missing data will be imputed in a sensitivity analysis.
Other comments	This outcome is ordinally distributed so a non-parametric approach will be used.

Outcome	E-cigarette use in last week
Effect size	Relative risk
Primary Analysis model	Log-binomial regression adjusting for factors stratified in the randomisation.
Sensitivity analysis	Log-binomial regression adjusting for factors stratified in the randomisation and factors pre-specified by TMG before database lock.
Missing data	Missing data will be imputed in a sensitivity analysis.
Other comments	

Table 17: Secondary efficacy outcomes

	ITT population		Minimally adjusted		Fully adjusted	
Outcome	Control (n=)	Intervention (n=)	Effect size (95%CI)	p-value	Effect size (95%CI)	p-value

Notley C, *et al. BMJ Open* 2023; 13:e076458. doi: 10.1136/bmjopen-2023-076458

NORWICH CTU

6.3 Missing Data

As mentioned in the above our primary analysis will replace missing abstinence values with relapse and the analysis of the other endpoints will be of available case. A sensitivity analysis will be conducted using multiple imputation assuming that the data are not missing at random. Alternative assumptions will be investigated but it will not be known which approaches/assumption are appropriate until we have more data about the missingness pattern. However, a reasonable NMAR choice would be to assume that those with missing data have worse outcome than those without missing data.

6.4 Additional analyses

6.5 Safety analyses

Only descriptive analysis of the SAE and SE will be reported. These will simply be listed as per the tables below.

Table 6.5.1: serious adverse events (incl. event description, duration, relationship to intervention)

Group	Date of onset	Description	Date of resolution	Related to trial treatment	Randomised group

Table 6.5.2: adverse events, by event, severity, or if appropriate, by relationship to intervention (including duration of treatment exposure), body compartment/system:

Group	Date of onset	Description	Date of resolution	Related to trial treatment	Randomised group

6.5 Software

Stata 17.1 or higher will be used for the majority of the analyses, however alternative software may be used if required.

Notley C, *et al. BMJ Open* 2023; 13:e076458. doi: 10.1136/bmjopen-2023-076458

NORWICH CTU

7.0 References

Cohen, S., Kamarck, T., & Mermelstein, R. (1983). A global measure of perceived stress. Journal of Health and Social Behavior, 24, 385-396.

Cox, J.L., Holden, J.M., and Sagovsky, R. 1987.Detection of postnatal depression: Development of the 10-item Edinburgh Postnatal Depression Scale. British Journal of Psychiatry 150:782-786.

Notley C, *et al. BMJ Open* 2023; 13:e076458. doi: 10.1136/bmjopen-2023-076458
