# Supplementary material for: BabyBreathe trial: protocol for a randomised controlled trial of a complex intervention to prevent postpartum return to smoking
Source: BMJ Open. 2023 Sep 4;13(9):e076458. doi: 10.1136/bmjopen-2023-076458 (PMC10481735; doi:10.1136/bmjopen-2023-076458)
Supplement: Author's manuscript [file bmjopen-2023-076458.draft_revisions.pdf]

BMJ Open

BMJ Open is committed to open peer review. As part of this commitment we make the peer review history of every article we publish publicly available.

When an article is published we post the peer reviewers' comments and the authors' responses online. We also post the versions of the paper that were used during peer review. These are the versions that the peer review comments apply to.

The versions of the paper that follow are the versions that were submitted during the peer review process. They are not the versions of record or the final published versions. They should not be cited or distributed as the published version of this manuscript.

BMJ Open is an open access journal and the full, final, typeset and author-corrected version of record of the manuscript is available on our site with no access controls, subscription charges or pay-per-view fees (http://bmjopen.bmj.com).

If you have any questions on BMJ Open's open peer review process please email info.bmjopen@bmj.com

THE BABYBREATHE TRIAL: PROTOCOL FOR A RANDOMISED CONTROLLED TRIAL OF A COMPLEX INTERVENTION TO PREVENT POSTPARTUM RETURN TO SMOKING

Journal:	*BMJ Open*
Manuscript ID	bmjopen-2023-076458
Article Type:	Protocol
Date Submitted by the Author:	11-Jun-2023

Complete List of Authors:	Notley, Caitlin; University of East Anglia, Norwich Medical School; University of East Anglia, University of East Anglia<br>Brown, Tracey; University of East Anglia, Norwich Medical School<br>Bauld, Linda; University of Edinburgh, Usher Institute of Population Health Sciences and Informatics<br>Clark, Allan; University of East Anglia, Norwich Medical School<br>Duneclift, Sharon; Norfolk Healthy Child Programme<br>Gilroy, Vicky; Institute of Health Visiting<br>Harris, Tess; St George’s University of London, Population Health Research Institute<br>Hardeman, Wendy; University of East Anglia, School of Health Sciences<br>Holland, Richard ; University of Leicester<br>Howard, Gregory; University of East Anglia<br>Man, Mei-See; University of East Anglia<br>Naughton, Felix; University of East Anglia Faculty of Medicine and Health Sciences, School of Health Sciences<br>Smith, Dan; University of East Anglia<br>Turner, David; University of East Anglia<br>Ussher, Michael; St George's University of London, Division of Population Health Sciences and Education; University of Stirling

Keywords:	Postpartum Period, PREVENTIVE MEDICINE, Primary Health Care

SCHOLARONE™
Manuscripts

THE BABYBREATHE™ TRIAL: PROTOCOL FOR A RANDOMISED CONTROLLED TRIAL OF A COMPLEX INTERVENTION TO PREVENT POSTPARTUM RETURN TO SMOKING

Caitlin Notley[1*], Tracey J. Brown[1], Linda Bauld[2], Allan Clark[3], Sharon Duneclift[4], Vicky Gilroy[5], Tess Harris[6], Wendy Hardeman[7], Richard Holland[8], Gregory Howard[3], Mei-See Man[3], Felix Naughton[7], Dan Smith[9], David Turner[3] Michael Ussher[6,10]

*Corresponding author. E mail: c.notley@uea.ac.uk; Twitter: @AddictionUEA

Author affiliations

[1] Faculty of Medicine and Health Sciences, Norwich Medical School, University of East Anglia, Norwich, UK

[2] The Usher Institute, College of Medicine and Veterinary Medicine, The University of Edinburgh, Edinburgh, UK

[3] Norwich Clinical Trials Unit, Norwich Medical School, Faculty of Medicine and Health Sciences, University of East Anglia, Norwich, UK

[4] Norfolk Healthy Child Programme, Whiting House, Whiting Road, Norwich. NR4 6DN, UK

[5] Institute of Health Visiting, UK

[6] Population Health Research Institute, St George's University of London, SW17 0RE, UK

[7] School of Health Sciences, University of East Anglia, Norwich, UK

[8] Leicester Medical School, University of Leicester, Leicester, UK

[9] School of Computing Sciences, University of East Anglia, Norwich, UK

[10] Institute for Social Marketing and Health, University of Stirling, Stirling FK9 4LA, UK

ABSTRACT

Introduction: Many people quit smoking during pregnancy, but postpartum smoking relapse is common. Maintaining smoking abstinence achieved during pregnancy is key to improving maternal and child health. There are no evidence-based interventions for preventing postpartum smoking relapse. This trial aims to determine whether an intervention to prevent post-partum relapse is effective and cost-effective.

Methods and analysis: A randomised controlled trial of a complex intervention to prevent postpartum smoking relapse (Babybreathe™), with internal pilot, economic and process evaluations. Participants are adults who are pregnant who report having quit smoking in the twelve months before, or during pregnancy. Participants are eligible if they read and understand English, and provide informed consent. Following consent and biochemical validation of smoking abstinence, participants are randomised to intervention or usual care/control (no specific relapse prevention support). The Babybreathe™ intervention consists of manualised advice from a trained member of the health visiting service, health information leaflets for participants and partners, access to the Babybreathe™ website and app. At the time of birth, participants are posted the Babybreathe box, and support is provided by text message for up to 12 months postpartum. Target sample size is 880, recruiting across midwifery services at four hubs in England and Scotland, and through remote advertising in England, Scotland, Wales and Northern Ireland. Outcomes are collected at 6 and 12

For peer review only - http://bmjopen.bmj.com/site/about/guidelines.xhtml

months. The primary outcome is self-reported sustained smoking abstinence at 12 months, carbon monoxide verified. Secondary outcomes include self-reported abstinence, time to relapse, partner smoking status and quality of life.

Ethics and dissemination: The trial was approved by the North West Preston Research Ethics committee (21/NW/0017). Dissemination will include publication in peer reviewed journals, presentation at academic and public conferences including PPI, and to policy makers and practitioners.

Trial registration: ISRCTN70307341, Trial Protocol v7, 04.05.2022

STRENGTHS AND LIMITATIONS OF THIS STUDY

- This is the largest international trial of a postpartum smoking relapse prevention intervention, specifically developed to support sustained postpartum smoking abstinence.
- The intervention (Babybreathe™) is theory based, drawing on behaviour change techniques, systematic reviews of existing evidence, and extensive patient and public involvement.
- An embedded mixed-methods process evaluation will assess implementation, mechanisms of impact and contextual influences, as well as acceptability and which elements of the intervention are perceived to be most effective, for which women, in which circumstances.
- The study is resource intensive and is limited by the capacity of clinical services. The trial protocol allows flexible options for recruitment and intervention delivery to support clinical teams in delivering the intervention.
- The trial is recruiting across the UK and includes a cost-effectiveness evaluation.

KEYWORDS

Postpartum, tobacco smoking, relapse prevention, behaviour change, health visitor

Word count: 3826

INTRODUCTION:

Around a quarter of UK women report smoking in the year before pregnancy (3). More women quit smoking during pregnancy than at any other time, with as many as 45% able to 'spontaneously quit' (4). However, there are marked health inequalities, as younger mothers and women with lower income are both less likely to quit and more likely to relapse (5,6). There is a unique opportunity to help women who cease smoking in pregnancy to quit permanently. Most women who quit smoking wish to remain abstinent after the birth; however, up to three-quarters of spontaneous quitters return to smoking within six months (7). Postpartum relapse is a major public health problem; yet there are no evidence-based interventions, and no routine support is offered to prevent relapse (8). The NHS Long Term Plan prioritises smoking cessation services in pregnancy (9), overlooking postpartum support. Supporting sustained abstinence may be critical to reaching the UK government 'smokefree 2030' target (8). This trial will build on the success of cessation interventions in pregnancy (11), by trialling a theory based relapse prevention intervention developed by our team (12).

For peer review only - http://bmjopen.bmj.com/site/about/guidelines.xhtml

Previous interventions to support sustained smoking abstinence postpartum consist of brief and skills-based education, but when pooled, studies overall did not demonstrate effectiveness (13). A recent Cochrane review of relapse prevention interventions included postpartum relapse prevention trials as a sub-group. Fifteen studies included postpartum follow-up but there was no significant benefit of interventions (8). New approaches are urgently needed to address this global public health issue. The recent Cochrane review concludes that: "Future studies may be better advised to focus on alternative approaches not studied extensively or at all so far, such as opportunistic use of nicotine replacement, contingency management, social support, cue exposure (only imaginary exposure has been studied so far), interventions aimed at maintaining abstainers' morale and awareness of the danger of slips, and so forth" (8). Sustained postpartum smoking abstinence has significant health benefits for the mother, as most new mothers will be young enough to minimise long-term harm, particularly from cancers and cardiovascular disease (14). Maternal smoking is the primary source of infant and child second-hand smoke exposure (15,16), a substantial cause of ill health and mortality (17). This has an intergenerational effect: children of smoking mothers are twice as likely to become smokers (18). The total NHS annual cost of smoking continuation, or returning to smoking following pregnancy, is estimated to range between £8.1 and £64 million annually for treating maternal health problems alone (19). Whilst, in 2015/16 the cost of admitted patient care in children attributable to passive smoking in England was an additional £5-12 million (20).

Following our comprehensive intervention development work and patient and public involvement (PPI), it is clear that postpartum smoking relapse is a complex problem requiring a multi-faceted solution. Our research team have developed a novel intervention combining behavioural, digital and relapse prevention support, 'BabyBreathe'. The intervention is theory based and uses behaviour change techniques, each supported by available evidence (21). The development process involved working with women, families and healthcare professionals to design an intervention that would fit in and work alongside usual care (universal health visiting service in the UK), be feasible to implement in practice, and be acceptable to women (12).

AIMS AND OBJECTIVES:

Aim:

To assess the effectiveness and cost-effectiveness of the BabyBreathe intervention in comparison to usual care, for supporting long-term smoking abstinence for mothers who have recently given birth and have stopped smoking during pregnancy or during the 12 months prior to pregnancy.

Objectives:

1. To run an internal pilot study, with clear stop/go trial embedded criteria, primarily to test recruitment systems.

2. To definitively test the effectiveness of BabyBreathe in comparison with usual care, by comparing smoking abstinence rates at 12 months postpartum between trial groups.

3. To undertake a cost-effectiveness analysis of BabyBreathe in comparison with usual care based on healthcare resource use of mother and infant and maternal health related quality of life (HRQoL).

4. To undertake an embedded mixed-methods process evaluation to assess delivery, implementation, fidelity and contamination, and to identify mechanisms of action by exploring which intervention components may be particularly effective, for which women, in which contexts.

For peer review only - http://bmjopen.bmj.com/site/about/guidelines.xhtml

METHODS AND ANALYSIS:

This protocol is reported in accordance with the SPIRIT recommendations (22) and the TiDIER guidelines for intervention description (23).

Trial design

BabyBreathe is a multi-centre, two-arm, superiority, parallel group, individually randomised, controlled trial of a complex intervention to prevent return to tobacco smoking postpartum, with internal pilot, including economic evaluation and process evaluation.

Study setting

The setting is 'real world' with the intervention integrated into, or offered as an adjunct to, standard antenatal and postnatal care. Trial recruitment hubs (Norfolk, London, North East of England, and Lothian, Scotland) have been selected to ensure a diverse sample, with an additional 'remote' recruitment hub to maximise recruitment rates (across the UK, including Wales and Northern Ireland).

Patient and public involvement

Two abstinent postpartum women were involved in development of intervention materials, and are included as members of our trial steering group, to advise on study progress and dissemination.

Population

We will seek pregnant women who have quit tobacco smoking in the 12 months before or during pregnancy, where smoking abstinence is defined as having stopped smoking for at least four weeks prior to recruitment.

Inclusion criteria:

1. Pregnant women who have stopped smoking completely in the 12 months prior to pregnancy, or at any time during pregnancy.
2. At 26 weeks gestation or any time following this up until birth, participant confirms having not smoked a single puff of a cigarette for at least four weeks.
3. Able to read and understand English.
4. Willing and able to give informed consent for participation in the study.
5. Expired carbon monoxide (CO) reading less than four parts per million (ppm) (24).

Exclusion criteria:

1. Under the age of 16

Recruitment and screening

Multiple recruitment strategies will be utilised to reach target sample size (n= 880). Potential participants will be identified by hospital and community midwives, research midwives (CRN) or sonographers, during routine antenatal appointments (e.g. booking appointment, routine scan appointment for dating or foetal anomaly scan), or by screening medical records. Participants may also be identified by smoke-free services, health visitors or by self-referring (e.g. via adverts in health or community settings, using targeted online recruitment, or media adverts). Women will be

For peer review only - http://bmjopen.bmj.com/site/about/guidelines.xhtml

screened for eligibility by the midwife (or by other healthcare professionals, in other health settings), or by a study researcher for direct referrals. The screening process can take place at any time during pregnancy, though the target is to identify participants ahead of 26 weeks pregnancy.

Eligible women will be provided a brief patient information leaflet, either directly or indirectly via an online link, explaining the study and permission will be requested to pass their contact details to the research team. A health professional or a research team member will enter their details into a study database (REDCap,(25)) that will automatically generate an SMS/email to an electronic Patient Information Sheet (PIS) and e-consent form containing full reassurance of confidentiality. If participants are unable or unwilling to consent electronically, study researchers will contact potential participants by telephone to complete consent. Once consent is completed, participants will provide further details so they can be contacted from 26 weeks pregnancy with the link to the eligibility confirmation questionnaire.

Participants will be asked to confirm eligibility by replying via a link sent by text or e mail (according to preference), and will provide their address to enable postage of a CO monitor (iCO monitor, Bedfont (25) in order to confirm eligibility using an expired CO reading of less than 4ppm (this is the standard cut off used in pregnancy (24). Participants will be asked to download the study specific CO monitor app (iCOBabyBreathe) which will provide the REDCap database with two CO readings. The highest of the two readings will be recorded. Where CO readings ≥26 weeks gestation are able to take place in person as part of standard care, CO readings may be obtained by a member of the clinical team or a researcher to confirm participant eligibility.

Once the participant has given informed consent and eligibility is confirmed through a CO reading, a link will be automatically generated through text/email to the participant to complete the baseline questionnaire.

Randomisation

Completion and submission of the baseline measures will trigger randomisation (see trial flow diagram, figure 1). Participants will be individually randomised in a 1:1 ratio to the control or intervention groups using a computerised web-based randomisation system managed and accessed only by Norwich Clinical Trials Unit, to ensure adequate sequence generation and allocation sequence concealment. The randomisation system will stratify by recruitment hub, partner smoking status (partner smoking and partner non-smoking/no partner) and time of quit (before or during pregnancy), as these factors are likely to predict relapse.

Insert figure 1 here

Blinding

Blinding is not possible due to the nature of the trial and intervention. The primary outcome is objectively assessed using biochemically validated CO verified smoking abstinence. Therefore, we consider that there is low risk of bias for the primary outcome.

Internal Pilot

The Independent Data Monitoring and Ethics Committee (DMEC) and Independent Trial Steering Committee (TSC) will scrutinise recruitment and protocol fidelity at six months into recruitment to establish continuation or stopping the trial at the pilot stage.

For peer review only - http://bmjopen.bmj.com/site/about/guidelines.xhtml

Trial Allocation groups:

Control

Control participants will receive usual antenatal and postnatal care as per the NHS maternity care pathway (i.e. no routine relapse prevention support). Usual care varies across the UK and sites have been purposively selected to reflect the variations in routine care. Commonalities in usual care across all the sites include pregnant women being routinely screened for smoking status by their midwife at their first antenatal booking appointment. If a participant reports that she is currently smoking, or she has a CO reading of 4ppm or more, she is automatically referred for stop smoking support via NHS commissioned stop smoking services (opt-out referral). Midwifery support focuses on abstinence in pregnancy and only includes brief advice about the opportunity to maintain abstinence postpartum and in the long-term. The usual care group will receive standard ante and postnatal care, as per mandated midwifery and health visitor contacts. These visits may be face-to-face appointments, or they may be remote video or telephone appointments due to the COVID-19 pandemic and local service provision protocols.

Intervention

Intervention participants will receive usual care plus the BabyBreathe™ package of support. The BabyBreathe ™ intervention is informed by the Capability Opportunity Motivation-Behaviour (COM-B) model and Behaviour Change Wheel (26), with full consideration of postpartum context-specific concerns. The intervention is a package of support designed to be delivered at low cost alongside usual care and existing healthcare services. All intervention components have been developed and initially tested in our preliminary work with women and partners (MRC MR/PO16944/1) (9). The intervention comprises three main stages:

1. **<u>Antenatal support up to birth:</u>**

A.) Usual care provision (as delivered in any given site area) will continue also to be provided to the intervention group.

B.) BabyBreathe™ relapse prevention leaflet.

C.) Partner/Friend/Relative relapse prevention leaflet – content has been designed to encourage partners/friends/relatives to support the participant to stay smoke free after delivery, and to promote active cessation for partners/friends/relatives themselves, where needed.

D.) Brief advice from a health visitor, heath visiting team member, or member of the research team trained to deliver the intervention (in-person, or remote). This advice is standardised and scripted following a protocol, with tailored options including positive praise for achieving smoking abstinence, and brief advice about the importance of staying smoke-free. Active signposting to the BabyBreathe digital /remote elements are included in this discussion.

E.) Electronic carbon monoxide testing – participants are given an iCO monitor (Bedfont) for individual use. Those in the intervention group will be encouraged to use the iCO monitor to self-monitor CO levels at any time during the study (control participants are only prompted to submit a research reading at baseline and study end).

F.) BabyBreathe website and app – these resources have been specifically developed and the app operates on both Android and iOS (iPhone) operating systems. The website and app can be accessed using a unique code. Users may input details such as the date they quit smoking, their estimated delivery date (EDD), and may access self-help support, health information and advice, including innovative motivational tools, such as a health calendar, and a closed online social support group, in preparation for entering the immediate postpartum period.

For peer review only - http://bmjopen.bmj.com/site/about/guidelines.xhtml

G.) At each subsequent health visitor contact (in-person, or remote), support for maintaining smoking abstinence will be briefly reiterated alongside usual care, where possible.

2. <u>Immediate postnatal period</u>

H.) BabyBreathe box – once the site team are alerted about the birth and input these data into the REDCap database, the central trial team will post out a BabyBreathe box. This is a physical box, designed to fit through a letter-box. The box includes self-incentive tools (e.g. reward chart, journal, photograph frame), free preventative NRT (Nicorette Icy White 2mg, 30 pieces), plus advice and support to use NRT or e-cigarettes for relapse prevention.

I.) SMS or app notification tailored support – This will be triggered by the birth notification, delivering a programme of tailored relapse prevention messages. Messages start daily, with a diminishing schedule over 12 months. At regular intervals participants are asked to confirm smoking status, and either then stay on the 'smokefree' or 'lapse' track of tailored messages. There is the option to opt out by texting 'stop' at any time.

3. <u>Postnatal period and beyond</u>

J.) At home/virtual postnatal visit with a health visitor, associated practitioner or BabyBreathe intervention trained researcher at around 10-14 days postpartum, when care is handed over from midwives to health visitors. At this visit, the health visitor will discuss smoking status, give positive praise, offer relapse prevention support, affirm that the BabyBreathe box has been received, and discuss contents of the BabyBreathe box, and text/app message use.

K.) Reiteration of support from health visitors or associated practitioners, up to 12 months postpartum – all subsequent postpartum routine health visitor appointments for the duration of the study will be undertaken by the same health visitor or health visiting team member where possible, , to assure continuity of care, which would be anticipated as part of usual practice. Positive praise will be offered for sustained smoking abstinence and the importance of relapse prevention will be emphasised. Particiapnts will be encouraged to continue to engage, or to re-engage, with the full suite of BabyBreathe resources. Cessation support (referral) will be offered to partners where necessary and appropriate. For those who relapse, referral for cessation support will also be offered.

See Figure 2 for examples of the components of the BabyBreathe intervention.

Insert Figure 2 here

Outcomes

See Table 1 for participant timeline of interventions and assessments.

Table 1: BABYBREATHE PARTICIPANT TIMELINE

Schedule of enrolment, interventions, and assessments.

	Antenatal				Birth	Postnatal				
	Screening	Confirm Eligibility (From	Baseline (From confirmatio	Health Visit (From randomisation up to birth)		Postnatal within 7 days	Health Visit (10-14 days Postpartum)	Health Visits (All subsequent routine)	6 month	12 month

For peer review only - http://bmjopen.bmj.com/site/about/guidelines.xhtml

	(From 8 weeks to birth)*	26 weeks)	n of eligibility)						follow up	follow up
Eligibility	X									
Consent to be contacted	X									
Link to PIS and Consent	X									
iCO reading to confirm eligibility		X								X
Demographics			X							
Smoking Status (SR)			X						X	X
Breastfeeding intention (27)			X						X	X
Relapse Predictors			X						X	X
Self-Efficacy (SR)			X						X	X
Edinburgh Depression Scale (28)			X						X	X
Behavioural Support (SR)			X						X	X
Nicotine Product Use (SR)			X						X	X
AUDIT-C (30)			X						X	X
EQ-5D-5L (45)			X						X	X
Cohen 4 item Perceived stress scale (31)			X						X	X
Randomisation			X							
BabyBreathe Intervention				X		X	X	X	X	X
Birth Notification						X				
Healthcare Resource Use(32)										X
Infant Health Outcomes										X

*Potential participants will be variously identified during pregnancy: identified at pregnancy booking visit, at dating scans at (8-12 weeks) and/or 20 week scan, by screening records, or at routine care pregnancy appointments occurring anytime between start of pregnancy and 26 weeks of pregnancy, and by self-referral through responding to adverts. These practices will vary and due to pandemic related restrictions of appointments that may happen remotely. Those missed can be eligible for recruitment following 26 weeks of pregnancy, until the end of pregnancy

Primary outcome:

The primary effectiveness outcome is self-reported continuous smoking abstinence, from birth, biochemically validated by CO monitoring at 12 months postpartum, with cut off of less than 8ppm (i.e. a reading of 7ppm or less) for those who are not pregnant, or with a cut off of less than 4ppm if they are pregnant at this time point, according to the Russell standard (33,34). Adapting the Russell standard for the postpartum population, we will grant a period of 'grace', allowing up to 5 smoking lapses (a one off instance of smoking) between the birth of the baby and the 12 month follow-up, before the outcome is counted as relapse. Participants will provide CO readings electronically, using the iCO monitor (Bedfont) and study app (iCOBabyBreathe) provided at baseline. Participants will

For peer review only

For peer review only - http://bmjopen.bmj.com/site/about/guidelines.xhtml

provide the REDCap database with two CO readings at entry and follow up. The highest of the readings will be recorded. Where CO readings take place in person as part of standard care, or research visits, or when participants request help with taking a CO reading, these readings may be used.

Secondary outcomes:
Secondary outcomes (Table 1) measured at 6 and 12 months postpartum by online self-report, or researcher follow-up, include self-reported point prevalence abstinence, self-reported time to relapse, participant-reported partner smoking status, self-efficacy (single item, self-report), Edinburgh postnatal depression scale(29), behavioural support use (e.g. support from a stop smoking service), nicotine product use, perceived stress (31), AUDIT-C, HRQoL using the EQ-5D-5L. Infant health outcomes (e.g. minor infections requiring GP visits and more serious ill health requiring hospitalisation), mother and infant health resource use and cost-effectiveness will be measured at 12 months postpartum using a combination of GP patient records and participant self-report.

Sample size

If the primary outcome (continued smoking abstinence) occurs in 25% of the control group (based on an estimated relapse rate of 75% (13)) compared with 35% of the intervention group, then we estimate that we will need 880 participants (440 per group, 220 per recruitment hub) to have 90% power to detect this 10% between group difference at the 5% level of significance. We estimate this difference between control and intervention groups is realistic based on recent trials (35). Loss to follow up or withdrawal is not considered within the sample size calculation, as all those lost to follow up will be counted as returned to smoking, as is the usual convention in smoking cessation trials (33).

Retention
To maximise retention and minimise loss to follow up, we will make the following efforts to retain contact with study participants. There will be one text/email reminder sent if links to questionnaires/forms are not followed by participants. If participants have not followed the initial links or reminders, then study researchers will contact up to five times to offer support, and collect self-report data where possible. Outcome data collection at 6 and 12 months flexibly includes electronic, phone, post and face-to-face options. Participants will also be offered reimbursement for their time (£15 shopping voucher) on completion of 12-month follow-up.

Data analysis
We will use descriptive statistics to present the baseline characteristics of the two study groups**.** We will use chi-squared tests to compare follow-up rates between the study groups, to establish whether there is differential drop out. Analysis of smoking status will be based on the intention-to-treat principle by analysing individuals according to the treatment they were allocated to regardless of compliance. Individuals for whom we do not have the primary outcome data will be assumed to have returned to smoking. Analysis of the primary outcome will be based on a logistic regression model, adjusting for the stratification variables used in the randomisation algorithm. Secondary analysis will adjust for factors known to be predictive of relapse which will be agreed with the TSC and added to the Statistical analysis plan (SAP) prior to analysis. Secondary outcomes will be analysed in a similar fashion using a general linear model. Missing data patterns will be examined, and if appropriate, multiple imputation will be undertaken. The SAP is pre-registered (On the open

For peer review only - http://bmjopen.bmj.com/site/about/guidelines.xhtml

science framework) – see appendix 1. The analysis plan may include analysis suggested by the qualitative analysis, such as subgroup analysis or mediation analysis. Any analysis will be pre-specified before data lock and published in the SAP prior to any data analysis.

Economic evaluation

An economic analysis will be conducted as an integral part of the randomised controlled trial. The primary perspective will be the NHS and social care: however, we will also look at broader relevant costs such as purchase of nicotine replacement therapies. All resources required to provide BabyBreathe will be recorded: these will include staff time; equipment; consumables; required staff training; and any other relevant costs. For staff time to carry out specific tasks to provide BabyBreathe a variety of methods to obtain these data will be explored: these would include trial records on relevant expenditure and expert opinion. Health care resource use will be obtained from two sources. Firstly, we will include a modified CSRI to obtain data by participant self-report at the 12 month follow-up. This will cover the following: maternal antenatal hospital admissions; details of delivery, including mode of delivery and length of stay; and infant NICU admissions. Contacts with GP and practice nurses, contact with other primary care practitioners and referral to secondary care will also be collected as well as smoking cessation related expenditure. Additionally, where feasible we will obtain data from patient notes and GP records All resources identified during the study will be valued using appropriate local and national unit cost data.

The main outcome measure used in the economic analysis will be the study's primary outcome measure, continuous post-partum smoking abstinence. This will form a cost-effectiveness study looking at cost per additional sustained abstainer. Additionally, we will use EQ-5D-5L (36) values obtained from participants to undertake a cost utility analysis (i.e. cost per QALY) estimating quality adjusted life years (QALYs), obtained at baseline, 6 and 12 months post-partum. EQ-5D-5L questionnaires will be valued using the most appropriate scoring algorithm at time of analysis. Currently, this would be the UK mapped scores (37). Cost and effectiveness data will be estimated using regression based methods to allow for differences in baseline characteristics between groups. Non parametric bootstrapping will be used to allow for uncertainty and this will also be used to construct a cost-effectiveness acceptability curve (CEAC), which shows how likely the intervention is to be cost-effective at different monetary values of the effectiveness measures. A health economics analysis plan will be agreed and published on the OSF before any analysis of health economics data.

Process evaluation

Both qualitative and quantitative data will be collected by the study research team to assess implementation of the intervention, mechanisms of impact and contextual influences, as per MRC guidance (38,39) (Table 2).

Table 2: Components of the BabyBreathe mixed methods process evaluation

Aims	Process evaluation component (Moore et al., BMJ 2015))	Method of data collection
Assess fidelity of BabyBreathe training	**Implementation**<br>Training	Questionnaires before and after training
Assess fidelity of intervention contacts	**Implementation** (intervention contacts)<br>Dose, reach, engagement	Log of visits by health visitor, health visiting practitioner, or researcher (participant level)

For peer review only - http://bmjopen.bmj.com/site/about/guidelines.xhtml

		Audio-recordings of 10% of contacts (antenatal and post-natal)<br>Qualitative interviews (health visitors, members of the health visiting team, or researcher – fidelity of delivery)<br>Qualitative interviews (participants and partners – engagement with visits and type of staff delivering the intervention)
Assess fidelity/engagement with the website and app	**Implementation** (website/app)<br>Dose, reach, engagement	Website and app data (number of log ins, total time in use)<br>Social support group threads<br>Number of texts received<br>Discontinuation of text/app notifications<br>Qualitative interviews (participants)
Assess contamination between trial arms	**Implementation** (intervention contacts)<br>Contamination	Recorded by trial research teams at each recruitment hub<br>Qualitative interviews (health visitors, members of the health visiting team, or researchers)<br>Health visitor feedback groups
Assess protocol modifications	**Implementation** (intervention contacts, website/app)<br>Fidelity, adaptations (intended and unintended/unforeseen; positive adaptations or drift)	Recorded by trial research teams at each recruitment hub<br>Qualitative interviews (health visitors, members of the health visiting team, or researchers)<br>Health visitor, member of the health visiting team, and researcher feedback groups
Assess how the intervention worked	**Mechanisms of impact**: hypothesised and unintended/unexpected pathways	Engagement data across recruitment hubs (visits)<br>Engagement with website and app<br>Engagement with text support<br>Use of Babybreathe box components (self-report, qualitative interviews and health visitor interviews)<br>Qualitative interviews (participants)
Assess contextual influences on implementation and mechanisms of impact	**Context:** contextual influences, e.g., participant/health visitor characteristics and geographical region, on implementation and mechanisms of impact	Qualitative interviews with health visitors, members of the health visiting team, or researchers, and participants

For peer review only

Assess the impact of the Covid-19 pandemic on intervention delivery and participant efforts to remain quit/stop smoking (partner)	**Implementation processes** (health visitor perspective)<br>Fidelity<br>Adaptions (by health visitors, members of the health visiting team, or researchers)<br>**Context**<br>Covid-19 pandemic response, e.g., restrictions, (partial) lockdowns.<br>**Mechanisms of impact**<br>Mediators	Qualitative interviews with health visitors, members of the health visiting team, or researchers, and participants

Fidelity of intervention delivery (*implementation*) and participant engagement with the health visitor visits and website/app will be assessed quantitatively through logs of visits, data analytics for website/app usage (the number of times that systems are logged on to, which resources are accessed, the time of engagement, the delivery of support messages via notifications and text messages, the time of any disengagement, discontinuation of SMS or app notifications, and self-reported engagement (as per (40)). Qualitative analysis will be undertaken of social support group threads, for which consent will have been sought on recruitment to the study; and audio-recordings (health visitors, practitioners or BabyBreathe researchers will be asked to record 10% of visits (≤10-mins intervention only), ante-natal as well as post-natal) and interviews with health visitors (n=12) and a qualitative interview subsample of participants and partners (n=40). Potential contamination between trial arms and protocol modifications will be assessed through qualitative interviews with health visitors and regular reporting by trial research teams. We will assess whether any identifiable modifications were planned adaptations to fit context, or unforeseen, and report our findings according to FRAME, an established framework (41). To illuminate possible mechanisms of action, a combined analysis of qualitative participant interview data, audio-recordings (e.g., intervention duration, delivery of behaviour change techniques) and quantitative engagement data across recruitment hubs will assess which components of the intervention were perceived to be particularly effective, for which people, in which contexts.

Data management

In view of the nature of the population (who are all expected to have one or more pregnancy related hospitalisation and primary care attendances which will be recorded in medical records); the intervention (which is not a medicinal product with the exception of Nicotine Replacement Therapy (gum) included in the BabyBreathe Box; and the trial primary and secondary outcomes, we do not intend to collect any additional safety endpoints.

BabyBreathe Trial Team members review the trial database to generate reports and review data entry. The essential trial issues, events and outputs, including defined key data points, are discussed by the trial team on a weekly basis and with relevant committees when necessary.

Ethics and dissemination:

Full REC and HRA approval has been granted (REC reference: 21/NW/0017, IRAS Project ID: 291746, protocol version 7 dated 04.05.2022. Participants provide electronic consent to take part, and rights of refusal to participate, or requests of withdrawal will be respected.

The results of the trial will be disseminated in open access journals, regardless of the direction of effect. The full protocol, statistical analysis plan, qualitative and health economics analysis plans, and anonymised datasets will be published in an online open access repository.

For peer review only - http://bmjopen.bmj.com/site/about/guidelines.xhtml

CONCLUSION

This trial will establish the effectiveness and cost effectiveness of a complex intervention to support sustained postpartum smoking abstinence. The process evaluation will establish which elements of the intervention are perceived to be most effective, for which people, in which circumstances. This trial is unique in testing a newly developed theory-based intervention, potentially delivering benefit for supporting sustained smoking abstinence, thus improving health outcomes for mothers, babies and wider family members.

Current study status: The first participant was randomised in September 2021. Recruitment is expected to take 24 months, with results expected to be published following final follow up in late 2024 or early 2025.

Acknowledgements:
We appreciate the support of the study sponsor, Norfolk & Waveney CCG. The study is led by researchers at the University of East Anglia (UEA) and managed by the Norwich Clinical Trials Unit (NCTU) at UEA. Our thanks go to all organisations involved in recruitment and in supporting delivery of the intervention, particularly health visiting services and midwifery services. We would also like to thank PPI contributors. We acknowledge the contribution of the researchers at sites who underwent training to deliver the intervention. Oversight provided by the independent Trial Steering Committee, comprising: Lion Shahab (Chair), Jo Leonardi-Bee, Jinshou Li, Siobhan Paul (PPI rep), Michael Twigg (Host rep) and Hilary Wareing. Sue Cooper, Graham Horne (sponsor rep), John Waldron, Libby White (PPI rep) and Julie Wright (funder rep); and Independent Data Monitoring committee: Paul Aveyard (Chair), Emma Beard and Karen Whittaker (terms of reference available upon request).

Author contributions: CN & TJB conceived the study idea, and drafted the manuscript. CN, TJB, WH, DS, MU & FN developed the intervention. GH is the trial manager and MM in the senior trial manager. AC is the trial statistician, DT is the health economist. LB, SD, VG, MU & TH are site PIs. RH provides public health academic trials expertise and DS provides computer science oversight.

Declaration of interests: All authors confirm that they have no competing financial or other potential conflicts of interest to declare.

Funding statement: This study is supported by the National Institute for Health and Care Research (NIHR) Public Health Research programme (project reference NIHR129074). The views expressed are those of the author(s) and not necessarily those of the NIHR or the Department of Health and Social Care.

REFERENCES

1. Patnode CD, Henderson JT, Coppola EL, Melnikow J, Durbin S, Thomas RG. Interventions for Tobacco Cessation in Adults, Including Pregnant Persons: Updated Evidence Report and Systematic Review for the US Preventive Services Task Force. JAMA. 2021 Jan 19;325(3):280–98.

2. Claire R, Chamberlain C, Davey M, Cooper SE, Berlin I, Leonardi-Bee J, et al. Pharmacological interventions for promoting smoking cessation during pregnancy. Cochrane Database Syst Rev. 2020 Mar 4;2020(3):CD010078.

For peer review only - http://bmjopen.bmj.com/site/about/guidelines.xhtml

3. Adult smoking habits in Great Britain - Office for National Statistics [Internet]. [cited 2019 Aug 7]. Available from: https://www.ons.gov.uk/peoplepopulationandcommunity/healthandsocialcare/drugusealcoholandsmoking/datasets/adultsmokinghabitsingreatbritain

4. Lumley J, Chamberlain C, Dowswell T, Oliver S, Oakley L, Watson L. Interventions for promoting smoking cessation during pregnancy. Cochrane Database Syst Rev. 2009 Jul 8;(3):CD001055.

5. Letourneau AR, Sonja B, Mazure CM, O'Malley SS, James D, Colson ER. Timing and predictors of postpartum return to smoking in a group of inner-city women: an exploratory pilot study. Birth. 2007 Sep;34(3):245–52.

6. Flemming K, Graham H, Heirs M, Fox D, Sowden A. Smoking in pregnancy: a systematic review of qualitative research of women who commence pregnancy as smokers. J Adv Nurs. 2013 May;69(5):1023–36.

7. Jones M, Lewis S, Parrott S, Wormall S, Coleman T. Re-starting smoking in the postpartum period after receiving a smoking cessation intervention: a systematic review. Addiction. 2016;111(6):981–90.

8. Livingstone-Banks J, Norris E, Hartmann-Boyce J, West R, Jarvis M, Hajek P. Relapse prevention interventions for smoking cessation. Cochrane Database Syst Rev. 2019 13;2:CD003999.

9. NHS England » NHS Long Term Plan [Internet]. [cited 2019 Mar 6]. Available from: https://www.england.nhs.uk/long-term-plan/

10. Smokefree 2030 – Smokefree Action Coalition [Internet]. [cited 2022 Jan 19]. Available from: https://smokefreeaction.org.uk/smokefree2030/

11. Chamberlain C, O'Mara-Eves A, Porter J, Coleman T, Perlen SM, Thomas J, McKenzie JE. Psychosocial interventions for supporting women to stop smoking in pregnancy. 2017, Issue 2. Art. No.: CD001055. [Internet]. Cochrane Database of Systematic Reviews; 2017. Available from: 10.1002/14651858.CD001055.pub5.

12. Notley C, Brown TJ, Bauld L, Hardeman W, Holland R, Naughton F, et al. Development of a Complex Intervention for the Maintenance of Postpartum Smoking Abstinence: Process for Defining Evidence-Based Intervention. International Journal of Environmental Research and Public Health. 2019 Jan;16(11):1968.

13. Hajek P, Stead LF, West R, Jarvis M, Hartmann-Boyce J, Lancaster T. Relapse prevention interventions for smoking cessation. Cochrane Database Syst Rev. 2013 Aug 20;(8):CD003999.

14. Doll R, Peto R, Boreham J, Sutherland I. Mortality in relation to smoking: 50 years' observations on male British doctors. BMJ. 2004 Jun 24;328(7455):1519.

For peer review only

For peer review only - http://bmjopen.bmj.com/site/about/guidelines.xhtml

15. Sims M, Tomkins S, Judge K, Taylor G, Jarvis MJ, Gilmore A. Trends in and predictors of second-hand smoke exposure indexed by cotinine in children in England from 1996 to 2006. Addiction. 2010 Mar;105(3):543–53.

16. DiFranza JR, Aligne CA, Weitzman M. Prenatal and postnatal environmental tobacco smoke exposure and children's health. Pediatrics. 2004 Apr;113(4 Suppl):1007–15.

17. Passive smoking and children [Internet]. RCP London. [cited 2018 Mar 20]. Available from: https://shop.rcplondon.ac.uk/products/passive-smoking-and-children

18. Leonardi-Bee J, Jere ML, Britton J. Exposure to parental and sibling smoking and the risk of smoking uptake in childhood and adolescence: a systematic review and meta-analysis. Thorax. 2011 Oct;66(10):847–55.

19. Godfrey C, Pickett KE, Parrot S et al. Estimating the costs to the NHS of smoking in pregnancy for pregnant women and infants. Department of Health Sciences, The University of York; 2010.

20. Royal College of Physicians. Hiding in plain sight. Treating tobacco dependency in the NHS. A report by the Tobacco Advisory Group of the Royal College of Physicians. 2018.

21. Brown TJ, Hardeman W, Bauld L, Holland R, Maskrey V, Naughton F, et al. A systematic review of behaviour change techniques within interventions to prevent return to smoking postpartum. Addict Behav. 2019 May;92:236–43.

22. Chan AW, Tetzlaff JM, Altman DG, Laupacis A, Gøtzsche PC, Krleža-Jerić K, et al. SPIRIT 2013 statement: defining standard protocol items for clinical trials. Ann Intern Med. 2013 Feb 5;158(3):200–7.

23. Hoffmann TC, Glasziou PP, Boutron I, Milne R, Perera R, Moher D, et al. Better reporting of interventions: template for intervention description and replication (TIDieR) checklist and guide. BMJ. 2014 Mar 7;348:g1687.

24. Bailey BA. Using expired air carbon monoxide to determine smoking status during pregnancy: preliminary identification of an appropriately sensitive and specific cut-point. Addict Behav. 2013 Oct;38(10):2547–50.

25. Harris PA, Taylor R, Thielke R, Payne J, Gonzalez N, Conde JG. Research electronic data capture (REDCap)—A metadata-driven methodology and workflow process for providing translational research informatics support. Journal of Biomedical Informatics. 2009 Apr 1;42(2):377–81.

26. Michie S, van Stralen MM, West R. The behaviour change wheel: A new method for characterising and designing behaviour change interventions. Implement Sci. 2011 Apr 23;6:42.

27. Bick D, Taylor C, Avery A, Bhavnani V, Craig V, Healey A, et al. Protocol for a two-arm feasibility RCT to support postnatal maternal weight management and positive lifestyle

For peer review only - http://bmjopen.bmj.com/site/about/guidelines.xhtml

behaviour in women from an ethnically diverse inner city population: the SWAN feasibility trial. Pilot Feasibility Stud. 2019 Oct 23;5:117.

28. Cox JL, Chapman G, Murray D, Jones P. Validation of the Edinburgh postnatal depression scale (EPDS) in non-postnatal women. Journal of Affective Disorders. 1996 Jul 29;39(3):185–9.

29. Bunevicius A, Kusminskas L, Pop VJ, Pedersen CA, Bunevicius R. Screening for antenatal depression with the Edinburgh Depression Scale. J Psychosom Obstet Gynaecol. 2009 Dec;30(4):238–43.

30. Bush K, Kivlahan DR, McDonell MB, Fihn SD, Bradley KA. The AUDIT alcohol consumption questions (AUDIT-C): an effective brief screening test for problem drinking. Ambulatory Care Quality Improvement Project (ACQUIP). Alcohol Use Disorders Identification Test. Arch Intern Med. 1998 Sep 14;158(16):1789–95.

31. Cohen S, Kamarck T, Mermelstein R. A Global Measure of Perceived Stress. Journal of Health and Social Behavior. 1983;24(4):385–96.

32. Beecham J, Knapp MRJ. Costing psychiatric interventions. In: Thornicroft G, editor. London: Royal College of Psychiatrists; 2001 [cited 2023 Mar 23]. p. 200–24. Available from: https://kar.kent.ac.uk/26426/

33. West R, Hajek P, Stead L, Stapleton J. Outcome criteria in smoking cessation trials: proposal for a common standard. Addiction. 2005 Mar;100(3):299–303.

34. Raiff BR, Faix C, Turturici M, Dallery J. Breath carbon monoxide output is affected by speed of emptying the lungs: Implications for laboratory and smoking cessation research. Nicotine Tob Res. 2010 Aug;12(8):834–8.

35. Pollak KI, Fish LJ, Lyna P, Peterson BL, Myers ER, Gao X, et al. Efficacy of a Nurse-Delivered Intervention to Prevent and Delay Postpartum Return to Smoking: The Quit for Two Trial. Nicotine Tob Res. 2016 Oct 1;18(10):1960–6.

36. Herdman M, Gudex C, Lloyd A, Janssen M, Kind P, Parkin D, et al. Development and preliminary testing of the new five-level version of EQ-5D (EQ-5D-5L). Qual Life Res. 2011 Dec;20(10):1727–36.

37. van Hout B, Janssen MF, Feng YS, Kohlmann T, Busschbach J, Golicki D, et al. Interim scoring for the EQ-5D-5L: mapping the EQ-5D-5L to EQ-5D-3L value sets. Value Health. 2012 Aug;15(5):708–15.

38. Process evaluation in randomised controlled trials of complex interventions | The BMJ [Internet]. [cited 2018 Nov 12]. Available from: https://www.bmj.com/content/332/7538/413

39. Craig P, Dieppe P, Macintyre S, Michie S, Nazareth I, Petticrew M. Developing and evaluating complex interventions: the new Medical Research Council guidance. BMJ

For peer review only - http://bmjopen.bmj.com/site/about/guidelines.xhtml

[Internet]. 2008 Sep 29;337. Available from: https://www.ncbi.nlm.nih.gov/pmc/articles/PMC2769032/

40. Groner J, French G, Ahijevych K, Wewers ME. Process Evaluation of a Nurse-Delivered Smoking Relapse Prevention Program for New Mothers. Journal of Community Health Nursing. 2005 Sep 1;22(3):157–67.

41. Wiltsey Stirman S, Baumann AA, Miller CJ. The FRAME: an expanded framework for reporting adaptations and modifications to evidence-based interventions. Implementation Science. 2019 Jun 6;14(1):58.

For peer review only

For peer review only - http://bmjopen.bmj.com/site/about/guidelines.xhtml

Figure 1: Babybreathe Trial flow diagram

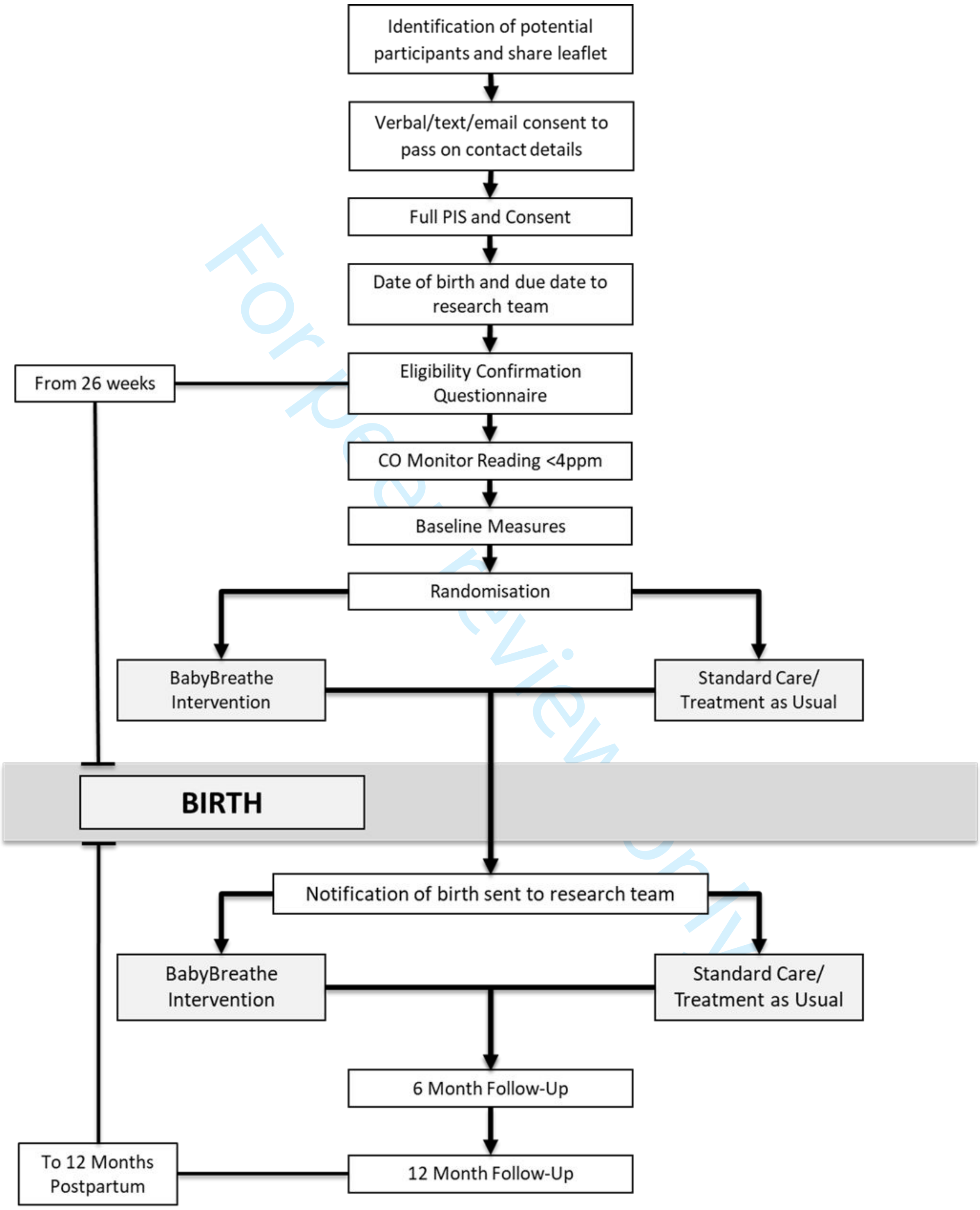

Figure 2: Examples of Babybreathe™ intervention components

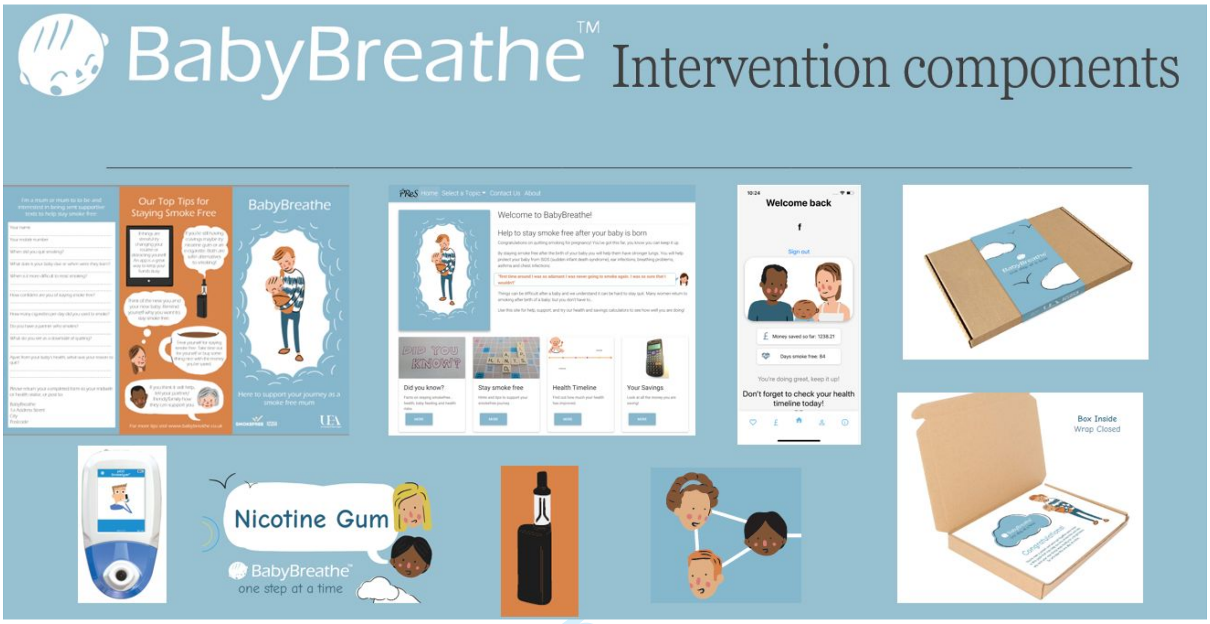

For peer review only

SPIRIT | CONSORT OUTCOMES Reporting Guidelines for Trial Outcomes

SPIRIT-Outcomes 2022 Checklist (for combined completion of SPIRIT 2013 and SPIRIT-Outcomes 2022 items)[a]

Section	Item No.	SPIRIT 2013 Item	SPIRIT-Outcomes 2022 item	Location Reported[b]
**Administrative information**				
Title	1	Descriptive title identifying the study design, population, interventions, and, if applicable, trial acronym	-	Pg 1
Trial registration	2a	Trial identifier and registry name. If not yet registered, name of intended registry	-	Pg 2
	2b	All items from the World Health Organization Trial Registration Data Set	-	N/A
Protocol version	3	Date and version identifier	-	Pg 2
Funding	4	Sources and types of financial, material, and other support	-	Pg 13
Roles and responsibilities	5a	Names, affiliations, and roles of protocol contributors	-	Pg 1, 13
	5b	Name and contact information for the trial sponsor	-	Pg 13
	5c	Role of study sponsor and funders, if any, in study design; collection, management, analysis, and interpretation of data; writing of the report; and the decision to submit the report for publication, including whether they will have ultimate authority over any of these activities	-	Pg 13
	5d	Composition, roles, and responsibilities of the coordinating centre, steering committee, endpoint adjudication committee, data management team, and other individuals or groups overseeing the trial, if applicable (see Item 21a for data monitoring committee)	-	Pg 13
**Introduction**				
Background and rationale	6a	Description of research question and justification for undertaking the trial, including summary of relevant studies (published and unpublished) examining benefits and harms for each intervention	-	Pg 2, 3
	6b	Explanation for choice of comparators	-	Pg 6
Objectives	7	Specific objectives or hypotheses	-	Pg 3,4

For peer review only

SPIRIT | CONSORT OUTCOMES
Reporting Guidelines for Trial Outcomes

Section	Item No.	SPIRIT 2013 Item	SPIRIT-Outcomes 2022 item	Location Reported[b]
Trial design	8	Description of trial design including type of trial (eg, parallel group, crossover, factorial, single group), allocation ratio, and framework (eg, superiority, equivalence, noninferiority, exploratory)	-	Pg 4
**Methods: Participants, interventions, and outcomes**				
Study setting	9	Description of study settings (eg, community clinic, academic hospital) and list of countries where data will be collected. Reference to where list of study sites can be obtained	-	Pg 4
Eligibility criteria	10	Inclusion and exclusion criteria for participants. If applicable, eligibility criteria for study centres and individuals who will perform the interventions (eg, surgeons, psychotherapists)	-	Pg 4
Interventions	11a	Interventions for each group with sufficient detail to allow replication, including how and when they will be administered (for specific guidance see TIDieR checklist and guide)	-	Pg 6,7
	11b	Criteria for discontinuing or modifying allocated interventions for a given trial participant (eg, drug dose change in response to harms, participant request, or improving/worsening disease)	-	Pg 12
	11c	Strategies to improve adherence to intervention protocols, and any procedures for monitoring adherence (eg, drug tablet return, laboratory tests)	-	Pg 11 (fidelity)
	11d	Relevant concomitant care and interventions that are permitted or prohibited during the trial	-	N/A
Outcomes	12	Primary, secondary, and other outcomes, including the specific measurement variable (eg, systolic blood pressure), analysis metric (eg, change from baseline, final value, time to event), method of aggregation (eg, median, proportion), and time point for each outcome. Explanation of the clinical relevance of chosen efficacy and harm outcomes is strongly recommended	-	Pg 8,9

For peer review only - http://bmjopen.bmj.com/site/about/guidelines.xhtml

Section	Item No.	SPIRIT 2013 Item	SPIRIT-Outcomes 2022 item	Location Reported[b]
	12.1		Provide a rationale for the selection of the domain for the trial's primary outcome	Pg 8
	12.2		If the analysis metric for the primary outcome represents within-participant change, define and justify the minimal important change in individuals	Pg 8
	12.3		If the outcome data collected are continuous but will be analyzed as categorical (method of aggregation), specify the cutoff values to be used	Pg 8
	12.4		If outcome assessments will be performed at several time points after randomization, state the time points that will be used for analysis	Pg 8
	12.5		If a composite outcome is used, define all individual components of the composite outcome	N/A
Participant timeline	13	Time schedule of enrolment, interventions (including any run-ins and washouts), assessments, and visits for participants. A schematic diagram is highly recommended (see Figure)	-	Pg 8
Sample size	14	Estimated number of participants needed to achieve study objectives and how it was determined, including clinical and statistical assumptions supporting any sample size calculations	-	Pg 9
	14.1		Define and justify the target difference between treatment groups (eg, the minimal important difference)	Pg 9
Recruitment	15	Strategies for achieving adequate participant enrolment to reach target sample size	-	Pg 5
**Methods: Assignment of interventions (for controlled trials)**				
Allocation:				
Sequence generation	16a	Method of generating the allocation sequence (eg, computer-generated random numbers), and list of any factors for stratification. To reduce predictability of a random sequence, details of any planned restriction (eg, blocking) should be provided in a separate document that is unavailable to those who enrol participants or assign interventions	-	Pg 5

For peer review only - http://bmjopen.bmj.com/site/about/guidelines.xhtml

Section	Item No.	SPIRIT 2013 Item	SPIRIT-Outcomes 2022 item	Location Reported[b]
Allocation concealment mechanism	16b	Mechanism of implementing the allocation sequence (eg, central telephone; sequentially numbered, opaque, sealed envelopes), describing any steps to conceal the sequence until interventions are assigned	-	Pg 5
Implementation	16c	Who will generate the allocation sequence, who will enrol participants, and who will assign participants to interventions	-	Pg 5
Blinding (masking)	17a	Who will be blinded after assignment to interventions (eg, trial participants, care providers, outcome assessors, data analysts), and how	-	Pg 5
	17b	If blinded, circumstances under which unblinding is permissible, and procedure for revealing a participant's allocated intervention during the trial	-	N/A
**Methods: Data collection, management, and analysis**				
Data collection methods	18a	Plans for assessment and collection of outcome, baseline, and other trial data, including any related processes to promote data quality (eg, duplicate measurements, training of assessors) and a description of study instruments (eg, questionnaires, laboratory tests) along with their reliability and validity, if known. Reference to where data collection forms can be found, if not in the protocol	-	Pg 8,9
	18a.1		Describe what is known about the responsiveness of the study instruments in a population similar to the study sample	References given for validated instruments used pg 8,9
	18a.2		Describe who will assess the outcome (eg, nurse, parent)	Pg 8,9
	18b	Plans to promote participant retention and complete follow-up, including list of any outcome data to be collected for participants who discontinue or deviate from intervention protocols	-	Pg 9, 12

For peer review only - http://bmjopen.bmj.com/site/about/guidelines.xhtml

Section	Item No.	SPIRIT 2013 Item	SPIRIT-Outcomes 2022 item	Location Reported[b]
Data management	19	Plans for data entry, coding, security, and storage, including any related processes to promote data quality (eg, double data entry; range checks for data values). Reference to where details of data management procedures can be found, if not in the protocol	-	Pg 12
Statistical methods	20a	Statistical methods for analysing primary and secondary outcomes. Reference to where other details of the statistical analysis plan can be found, if not in the protocol	-	Pg 9 and SAP
	20a.1		Describe any planned methods to account for multiplicity in the analysis or interpretation of the primary and secondary outcomes (eg, coprimary outcomes, same outcome assessed at multiple time points, or subgroup analyses of an outcome)	SAP
	20b	Methods for any additional analyses (eg, subgroup and adjusted analyses)	-	SAP
	20c	Definition of analysis population relating to protocol non-adherence (eg, as randomised analysis), and any statistical methods to handle missing data (eg, multiple imputation)	-	Pg 9 and SAP
**Methods: Monitoring**				
Data monitoring	21a	Composition of data monitoring committee (DMC); summary of its role and reporting structure; statement of whether it is independent from the sponsor and competing interests; and reference to where further details about its charter can be found, if not in the protocol. Alternatively, an explanation of why a DMC is not needed	-	Pg 13
	21b	Description of any interim analyses and stopping guidelines, including who will have access to these interim results and make the final decision to terminate the trial	-	pg 5, 6 (pilot)
Harms	22	Plans for collecting, assessing, reporting, and managing solicited and spontaneously reported adverse events and other unintended effects of trial interventions or trial conduct	-	Pg 12 and data management plan

For peer review only

SPIRIT | CONSORT OUTCOMES Reporting Guidelines for Trial Outcomes

Section	Item No.	SPIRIT 2013 Item	SPIRIT-Outcomes 2022 item	Location Reported[b]
Auditing	23	Frequency and procedures for auditing trial conduct, if any, and whether the process will be independent from investigators and the sponsor	-	N/A - defined by sponsor / funder
**Ethics and dissemination**				
Research ethics approval	24	Plans for seeking research ethics committee/institutional review board (REC/IRB) approval	-	Pg 12
Protocol amendments	25	Plans for communicating important protocol modifications (eg, changes to eligibility criteria, outcomes, analyses) to relevant parties (eg, investigators, REC/IRBs, trial participants, trial registries, journals, regulators)	-	Pg 12
Consent or assent	26a	Who will obtain informed consent or assent from potential trial participants or authorised surrogates, and how (see Item 32)	-	Pg 5
	26b	Additional consent provisions for collection and use of participant data and biological specimens in ancillary studies, if applicable	-	N/A
Confidentiality	27	How personal information about potential and enrolled participants will be collected, shared, and maintained in order to protect confidentiality before, during, and after the trial	-	Pg 5, PIS and data management plan
Declaration of interests	28	Financial and other competing interests for principal investigators for the overall trial and each study site	-	Pg 13
Access to data	29	Statement of who will have access to the final trial dataset, and disclosure of contractual agreements that limit such access for investigators	-	Data management plan
Ancillary and post-trial care	30	Provisions, if any, for ancillary and post-trial care, and for compensation to those who suffer harm from trial participation	-	N/A
Dissemination policy	31a	Plans for investigators and sponsor to communicate trial results to participants, healthcare professionals, the public, and other relevant groups (eg, via publication, reporting in results databases, or other data sharing arrangements), including any publication restrictions	-	Pg 13
	31b	Authorship eligibility guidelines and any intended use of professional writers	-	Pg 13

For peer review only - http://bmjopen.bmj.com/site/about/guidelines.xhtml

Section	Item No.	SPIRIT 2013 Item	SPIRIT-Outcomes 2022 item	Location Reported[b]
	31c	Plans, if any, for granting public access to the full protocol, participant-level dataset, and statistical code	-	Pg 13
**Appendices**				
Informed consent materials	32	Model consent form and other related documentation given to participants and authorised surrogates	-	Appendix
Biological specimens	33	Plans for collection, laboratory evaluation, and storage of biological specimens for genetic or molecular analysis in the current trial and for future use in ancillary studies, if applicable	-	N/A

[a]It is strongly recommended that this checklist be read in conjunction with the SPIRIT (Standard Protocol Items: Recommendations for Interventional Trials) Statement paper for important clarification on the items. Amendments to the protocol should be tracked and dated. The SPIRIT checklist is copyrighted by the SPIRIT Group under the Creative Commons "Attribution-NonCommercial-NoDerivs 3.0 Unported" license and is reproduced with permission.
[b]Indicates page numbers and/or manuscript location: to be completed by authors.

For peer review only

TIDieR Template for Intervention Description and Replication

The TIDieR (Template for Intervention Description and Replication) Checklist*:

Information to include when describing an intervention and the location of the information

Item number	Item	Where located ** Primary paper (page or appendix number)	Other † (details)
	**BRIEF NAME**		
**1.**	Provide the name or a phrase that describes the intervention.	__Title page__	'Babybreathe'
	**WHY**		
**2.**	Describe any rationale, theory, or goal of the elements essential to the intervention.	___page 3___	______________
	**WHAT**		
**3.**	Materials: Describe any physical or informational materials used in the intervention, including those provided to participants or used in intervention delivery or in training of intervention providers. Provide information on where the materials can be accessed (e.g. online appendix, URL).	Page 6, 7	______________
**4.**	Procedures: Describe each of the procedures, activities, and/or processes used in the intervention, including any enabling or support activities.	Page 6,7	______________
	**WHO PROVIDED**		
**5.**	For each category of intervention provider (e.g. psychologist, nursing assistant), describe their expertise, background and any specific training given.	__page 6, 7__	______________
	**HOW**		
**6.**	Describe the modes of delivery (e.g. face-to-face or by some other mechanism, such as internet or telephone) of the intervention and whether it was provided individually or in a group.	__page 6, 7__	______________
	**WHERE**		
**7.**	Describe the type(s) of location(s) where the intervention occurred, including any necessary infrastructure or relevant features.	___page 6, 7_	______________

For peer review only

For peer review only - http://bmjopen.bmj.com/site/about/guidelines.xhtml

	**WHEN and HOW MUCH**		
**8.**	Describe the number of times the intervention was delivered and over what period of time including the number of sessions, their schedule, and their duration, intensity or dose.	__page 7____	______________
	**TAILORING**		
**9.**	If the intervention was planned to be personalised, titrated or adapted, then describe what, why, when, and how.	___page 6, 7__	______________
	**MODIFICATIONS**		
**10.‡**	If the intervention was modified during the course of the study, describe the changes (what, why, when, and how).	____N/A______ ___	______________
	**HOW WELL**		
**11.**	Planned: If intervention adherence or fidelity was assessed, describe how and by whom, and if any strategies were used to maintain or improve fidelity, describe them.	__ page 10,11	______________
**12.‡**	Actual: If intervention adherence or fidelity was assessed, describe the extent to which the intervention was delivered as planned.	___N/A_______ ___	NA at protocol stage

** **Authors** - use N/A if an item is not applicable for the intervention being described. **Reviewers** – use '?' if information about the element is not reported/not sufficiently reported.

† If the information is not provided in the primary paper, give details of where this information is available. This may include locations such as a published protocol or other published papers (provide citation details) or a website (provide the URL).

‡ If completing the TIDieR checklist for a protocol, these items are not relevant to the protocol and cannot be described until the study is complete.

* We strongly recommend using this checklist in conjunction with the TIDieR guide (see *BMJ* 2014;348:g1687) which contains an explanation and elaboration for each item.

* The focus of TIDieR is on reporting details of the intervention elements (and where relevant, comparison elements) of a study. Other elements and methodological features of studies are covered by other reporting statements and checklists and have not been duplicated as part of the TIDieR checklist. When a **randomised trial** is being reported, the TIDieR checklist should be used in conjunction with the CONSORT statement (see www.consort-statement.org) as an extension of **Item 5 of the CONSORT 2010 Statement.** When a **clinical trial protocol** is being reported, the TIDieR checklist should be used in conjunction with the SPIRIT statement as an extension of **Item 11 of the SPIRIT 2013 Statement** (see www.spirit-statement.org). For alternate study designs, TIDieR can be used in conjunction with the appropriate checklist for that study design (see www.equator-network.org).

For peer review only - http://bmjopen.bmj.com/site/about/guidelines.xhtml

THE BABYBREATHETM TRIAL: PROTOCOL FOR A RANDOMISED CONTROLLED TRIAL OF A COMPLEX INTERVENTION TO PREVENT POSTPARTUM RETURN TO SMOKING

Journal:	*BMJ Open*
Manuscript ID	bmjopen-2023-076458.R1
Article Type:	Protocol
Date Submitted by the Author:	03-Aug-2023
Complete List of Authors:	Notley, Caitlin; University of East Anglia Norwich Medical School, Norwich Medical School; University of East Anglia<br>Brown, Tracey; University of East Anglia, Norwich Medical School<br>Bauld, Linda; University of Edinburgh, Usher Institute of Population Health Sciences and Informatics<br>Clark, Allan; University of East Anglia, Norwich Medical School<br>Duneclift, Sharon; Norfolk Healthy Child Programme<br>Gilroy, Vicky; Institute of Health Visiting<br>Harris, Tess; St George’s University of London, Population Health Research Institute<br>Hardeman, Wendy; University of East Anglia, School of Health Sciences<br>Holland, Richard ; University of Leicester<br>Howard, Gregory; University of East Anglia<br>Man, Mei-See; University of East Anglia<br>Naughton, Felix; University of East Anglia Faculty of Medicine and Health Sciences, School of Health Sciences<br>Smith, Dan; University of East Anglia<br>Turner, David; University of East Anglia<br>Ussher, Michael; St George's University of London, Division of Population Health Sciences and Education; University of Stirling
**Primary Subject Heading**:	Smoking and tobacco
Secondary Subject Heading:	Public health, Obstetrics and gynaecology
Keywords:	Postpartum Period, PREVENTIVE MEDICINE, Primary Health Care

THE BABYBREATHE™ TRIAL: PROTOCOL FOR A RANDOMISED CONTROLLED TRIAL OF A COMPLEX INTERVENTION TO PREVENT POSTPARTUM RETURN TO SMOKING

Caitlin Notley[1*], Tracey J. Brown[1], Linda Bauld[2], Allan Clark[3], Sharon Duneclift[4], Vicky Gilroy[5], Tess Harris[6], Wendy Hardeman[7], Richard Holland[8], Gregory Howard[3], Mei-See Man[3], Felix Naughton[7], Dan Smith[9], David Turner[3] Michael Ussher[6,10]

*Corresponding author. E mail: c.notley@uea.ac.uk; Twitter: @AddictionUEA

Author affiliations

[1] Faculty of Medicine and Health Sciences, Norwich Medical School, University of East Anglia, Norwich, UK

[2] The Usher Institute, College of Medicine and Veterinary Medicine, The University of Edinburgh, Edinburgh, UK

[3] Norwich Clinical Trials Unit, Norwich Medical School, Faculty of Medicine and Health Sciences, University of East Anglia, Norwich, UK

[4] Norfolk Healthy Child Programme, Whiting House, Whiting Road, Norwich. NR4 6DN, UK

[5] Institute of Health Visiting, UK

[6] Population Health Research Institute, St George's University of London, SW17 0RE, UK

[7] School of Health Sciences, University of East Anglia, Norwich, UK

[8] Leicester Medical School, University of Leicester, Leicester, UK

[9] School of Computing Sciences, University of East Anglia, Norwich, UK

[10] Institute for Social Marketing and Health, University of Stirling, Stirling FK9 4LA, UK

ABSTRACT

Introduction: Many people quit smoking during pregnancy, but postpartum smoking relapse is common. Maintaining smoking abstinence achieved during pregnancy is key to improving maternal and child health. There are no evidence-based interventions for preventing postpartum smoking relapse. This trial aims to determine whether an intervention to prevent post-partum relapse is effective and cost-effective.

Methods and analysis: A randomised controlled trial of a complex intervention to prevent postpartum smoking relapse (Babybreathe™), with internal pilot, economic and process evaluations. Participants are adults who are pregnant who report having quit smoking in the twelve months before, or during pregnancy. Participants are eligible if they read and understand English, and provide informed consent. Following consent and biochemical validation of smoking abstinence, participants are randomised to intervention or usual care/control (no specific relapse prevention support). The Babybreathe™ intervention consists of manualised advice from a trained member of the health visiting service, health information leaflets for participants and partners, access to the Babybreathe™ website and app. At the time of birth, participants are posted the Babybreathe box, and support is provided by text message for up to 12 months postpartum. Target sample size is 880, recruiting across midwifery services at four hubs in England and Scotland, and through remote advertising in England, Scotland, Wales and Northern Ireland. Outcomes are collected at 6 and 12

For peer review only - http://bmjopen.bmj.com/site/about/guidelines.xhtml

months. The primary outcome is self-reported sustained smoking abstinence at 12 months, carbon monoxide verified. Secondary outcomes include self-reported abstinence, time to relapse, partner smoking status and quality of life.

Ethics and dissemination: The trial was approved by the North West Preston Research Ethics committee (21/NW/0017). Dissemination will include publication in peer reviewed journals, presentation at academic and public conferences including PPI, and to policy makers and practitioners.

Trial registration: ISRCTN70307341, Trial Protocol v7, 04.05.2022

STRENGTHS AND LIMITATIONS OF THIS STUDY

- This is the largest international trial of a postpartum smoking relapse prevention intervention, specifically developed to support sustained postpartum smoking abstinence.
- The intervention (Babybreathe™) is theory based, drawing on behaviour change techniques, systematic reviews of existing evidence, and extensive patient and public involvement.
- An embedded mixed-methods process evaluation will assess implementation, mechanisms of impact and contextual influences, as well as acceptability and which elements of the intervention are perceived to be most effective, for which women, in which circumstances.
- The study is resource intensive and is limited by the capacity of clinical services. The trial protocol allows flexible options for recruitment and intervention delivery to support clinical teams in delivering the intervention.
- The trial is recruiting across the UK and includes a cost-effectiveness evaluation.

KEYWORDS

Postpartum, tobacco smoking, relapse prevention, behaviour change, health visitor

Word count: 3826

INTRODUCTION:

Around a quarter of UK women report smoking in the year before pregnancy (1–3). More women quit smoking during pregnancy than at any other time, with as many as 45% able to 'spontaneously quit' (4). However, there are marked health inequalities, as younger mothers and women with lower income are both less likely to quit and more likely to relapse(5,6). There is a unique opportunity to help women who cease smoking in pregnancy to quit permanently. Most women who quit smoking wish to remain abstinent after the birth; however, up to three-quarters of spontaneous quitters return to smoking within six months(7). Postpartum relapse is a major public health problem; yet there are no evidence-based interventions, and no routine support is offered to prevent relapse(8). The NHS Long Term Plan prioritises smoking cessation services in pregnancy(9), overlooking postpartum support. Supporting sustained abstinence may be critical to reaching the UK government 'smokefree 2030' target(10). This trial will build on the success of cessation interventions in pregnancy(11), by trialling a theory based relapse prevention intervention developed by our team(12).

For peer review only - http://bmjopen.bmj.com/site/about/guidelines.xhtml

Previous interventions to support sustained smoking abstinence postpartum consist of brief and skills-based education, but when pooled, studies overall did not demonstrate effectiveness(13). A recent Cochrane review of relapse prevention interventions included postpartum relapse prevention trials as a sub-group. Fifteen studies included postpartum follow-up but there was no significant benefit of interventions(8). New approaches are urgently needed to address this global public health issue. The recent Cochrane review concludes that: "Future studies may be better advised to focus on alternative approaches not studied extensively or at all so far, such as opportunistic use of nicotine replacement, contingency management, social support, cue exposure (only imaginary exposure has been studied so far), interventions aimed at maintaining abstainers' morale and awareness of the danger of slips, and so forth"(8). Sustained postpartum smoking abstinence has significant health benefits for the mother, as most new mothers will be young enough to minimise long-term harm, particularly from cancers and cardiovascular disease(14). Maternal smoking is the primary source of infant and child second-hand smoke exposure(15,16), a substantial cause of ill health and mortality(17). This has an intergenerational effect: children of smoking mothers are twice as likely to become smokers(18). The total NHS annual cost of smoking continuation, or returning to smoking following pregnancy, is estimated to range between £8.1 and £64 million annually for treating maternal health problems alone(19). Whilst, in 2015/16 the cost of admitted patient care in children attributable to passive smoking in England was an additional £5-12 million(20).

Following our comprehensive intervention development work and patient and public involvement (PPI), it is clear that postpartum smoking relapse is a complex problem requiring a multi-faceted solution. Our research team have developed a novel intervention combining behavioural, digital and relapse prevention support, 'BabyBreathe'. The intervention is theory based and uses behaviour change techniques, each supported by available evidence(21). The development process involved working with women, families and healthcare professionals to design an intervention that would fit in and work alongside usual care (universal health visiting service in the UK), be feasible to implement in practice, and be acceptable to women(12).

AIMS AND OBJECTIVES:

Aim*:*

To assess the effectiveness and cost-effectiveness of the BabyBreathe intervention in comparison to usual care, for supporting long-term smoking abstinence for mothers who have recently given birth and have stopped smoking during pregnancy or during the 12 months prior to pregnancy.

Objectives:

1. To run an internal pilot study, with clear stop/go trial embedded criteria, primarily to test recruitment systems.

2. To definitively test the effectiveness of BabyBreathe in comparison with usual care, by comparing smoking abstinence rates at 12 months postpartum between trial groups.

3. To undertake a cost-effectiveness analysis of BabyBreathe in comparison with usual care based on healthcare resource use of mother and infant and maternal health related quality of life (HRQoL).

4. To undertake an embedded mixed-methods process evaluation to assess delivery, implementation, fidelity and contamination, and to identify mechanisms of action by exploring which intervention components may be particularly effective, for which women, in which contexts.

For peer review only - http://bmjopen.bmj.com/site/about/guidelines.xhtml

METHODS AND ANALYSIS:

This protocol is reported in accordance with the SPIRIT recommendations(22) and the TiDIER guidelines for intervention description(23) (see supplementary file).

Trial design

BabyBreathe is a multi-centre, two-arm, superiority, parallel group, individually randomised, controlled trial of a complex intervention to prevent return to tobacco smoking postpartum, with internal pilot, including economic evaluation and process evaluation.

Study setting

The setting is 'real world' with the intervention integrated into, or offered as an adjunct to, standard antenatal and postnatal care. Trial recruitment hubs (Norfolk, London, North East of England, and Lothian, Scotland) have been selected to ensure a diverse sample, with an additional 'remote' recruitment hub to maximise recruitment rates (across the UK, including Wales and Northern Ireland).

Patient and public involvement

Two abstinent postpartum women were involved in development of intervention materials, and are included as members of our trial steering group, to advise on study progress and dissemination.

Population

We will seek pregnant women who have quit tobacco smoking in the 12 months before or during pregnancy, where smoking abstinence is defined as having stopped smoking for at least four weeks prior to recruitment.

Inclusion criteria:

1. Pregnant women who have stopped smoking completely in the 12 months prior to pregnancy, or at any time during pregnancy.
2. At 26 weeks gestation or any time following this up until birth, participant confirms having not smoked a single puff of a cigarette for at least four weeks.
3. Able to read and understand English.
4. Willing and able to give informed consent for participation in the study.
5. Expired carbon monoxide (CO) reading less than four parts per million (ppm)(24).

Exclusion criteria:

1. Under the age of 16

Recruitment and screening

Multiple recruitment strategies will be utilised to reach target sample size (n= 880). Potential participants will be identified by hospital and community midwives, research midwives (CRN) or sonographers, during routine antenatal appointments (e.g. booking appointment, routine scan appointment for dating or foetal anomaly scan), or by screening medical records. Participants may also be identified by smoke-free services, health visitors or by self-referring (e.g. via adverts in health or community settings, using targeted online recruitment, or media adverts). Women will be screened for eligibility by the midwife (or by other healthcare professionals, in other health settings),

For peer review only - http://bmjopen.bmj.com/site/about/guidelines.xhtml

or by a study researcher for direct referrals. The screening process can take place at any time during pregnancy, though the target is to identify participants ahead of 26 weeks pregnancy.

Eligible women will be provided a brief patient information leaflet, either directly or indirectly via an online link, explaining the study and permission will be requested to pass their contact details to the research team. A health professional or a research team member will enter their details into a study database (REDCap,(25)) that will automatically generate an SMS/email to an electronic Patient Information Sheet (PIS) and e-consent form containing full reassurance of confidentiality. If participants are unable or unwilling to consent electronically, study researchers will contact potential participants by telephone to complete consent. Once consent is completed, participants will provide further details so they can be contacted from 26 weeks pregnancy with the link to the eligibility confirmation questionnaire.

Participants will be asked to confirm eligibility by replying via a link sent by text or e mail (according to preference), and will provide their address to enable postage of a CO monitor (iCO monitor, Bedfont in order to confirm eligibility using an expired CO reading of less than 4ppm (this is the standard cut off used in pregnancy(24). Participants will be asked to download the study specific CO monitor app (iCOBabyBreathe) which will provide the REDCap database with two CO readings. The highest of the two readings will be recorded. Where CO readings ≥26 weeks gestation are able to take place in person as part of standard care, CO readings may be obtained by a member of the clinical team or a researcher to confirm participant eligibility.

Once the participant has given informed consent and eligibility is confirmed through a CO reading, a link will be automatically generated through text/email to the participant to complete the baseline questionnaire.

Randomisation

Completion and submission of the baseline measures will trigger randomisation (see trial flow diagram, figure 1). Participants will be individually randomised in a 1:1 ratio to the control or intervention groups using a computerised web-based randomisation system managed and accessed only by Norwich Clinical Trials Unit, to ensure adequate sequence generation and allocation sequence concealment. The randomisation system will stratify by recruitment hub, partner smoking status (partner smoking and partner non-smoking/no partner) and time of quit (before or during pregnancy), as these factors are likely to predict relapse.

Insert figure 1 here

Blinding

Blinding is not possible due to the nature of the trial and intervention. The primary outcome is objectively assessed using biochemically validated CO verified smoking abstinence. Therefore, we consider that there is low risk of bias for the primary outcome.

Internal Pilot

The Independent Data Monitoring and Ethics Committee (DMEC) and Independent Trial Steering Committee (TSC) will scrutinise recruitment and protocol fidelity at six months into recruitment to establish continuation or stopping the trial at the pilot stage.

Trial Allocation groups:

For peer review only - http://bmjopen.bmj.com/site/about/guidelines.xhtml

Control

Control participants will receive usual antenatal and postnatal care as per the NHS maternity care pathway (i.e. no routine relapse prevention support). Usual care varies across the UK and sites have been purposively selected to reflect the variations in routine care. Commonalities in usual care across all the sites include pregnant women being routinely screened for smoking status by their midwife at their first antenatal booking appointment. If a participant reports that she is currently smoking, or she has a CO reading of 4ppm or more, she is automatically referred for stop smoking support via NHS commissioned stop smoking services (opt-out referral). Midwifery support focuses on abstinence in pregnancy and only includes brief advice about the opportunity to maintain abstinence postpartum and in the long-term. The usual care group will receive standard ante and postnatal care, as per mandated midwifery and health visitor contacts. These visits may be face-to-face appointments, or they may be remote video or telephone appointments due to the COVID-19 pandemic and local service provision protocols.

Intervention

Intervention participants will receive usual care plus the BabyBreathe™ package of support. The BabyBreathe ™ intervention is informed by the Capability Opportunity Motivation-Behaviour (COM-B) model and Behaviour Change Wheel(26), with full consideration of postpartum context-specific concerns. The intervention is a package of support designed to be delivered at low cost alongside usual care and existing healthcare services. All intervention components have been developed and initially tested in our preliminary work with women and partners (MRC MR/PO16944/1) (12). The intervention comprises three main stages:

1. **<u>Antenatal support up to birth:</u>**

A.) Usual care provision (as delivered in any given site area) will continue also to be provided to the intervention group.

B.) BabyBreathe™ relapse prevention leaflet.

C.) Partner/Friend/Relative relapse prevention leaflet – content has been designed to encourage partners/friends/relatives to support the participant to stay smoke free after delivery, and to promote active cessation for partners/friends/relatives themselves, where needed.

D.) Brief advice from a health visitor, heath visiting team member, or member of the research team trained to deliver the intervention (in-person, or remote). This advice is standardised and scripted following a protocol, with tailored options including positive praise for achieving smoking abstinence, and brief advice about the importance of staying smoke-free. Active signposting to the BabyBreathe digital /remote elements are included in this discussion.

E.) Electronic carbon monoxide testing – participants are given an iCO monitor (Bedfont) for individual use. Those in the intervention group will be encouraged to use the iCO monitor to self-monitor CO levels at any time during the study (control participants are only prompted to submit a research reading at baseline and study end).

F.) BabyBreathe website and app – these resources have been specifically developed and the app operates on both Android and iOS (iPhone) operating systems. The website and app can be accessed using a unique code. Users may input details such as the date they quit smoking, their estimated delivery date (EDD), and may access self-help support, health information and advice, including innovative motivational tools, such as a health calendar, and a closed online social support group, in preparation for entering the immediate postpartum period.

G.) At each subsequent health visitor contact (in-person, or remote), support for maintaining smoking abstinence will be briefly reiterated alongside usual care, where possible.

For peer review only - http://bmjopen.bmj.com/site/about/guidelines.xhtml

2. **Immediate postnatal period**

H.) BabyBreathe box – once the site team are alerted about the birth and input these data into the REDCap database, the central trial team will post out a BabyBreathe box. This is a physical box, designed to fit through a letter-box. The box includes self-incentive tools (e.g. reward chart, journal, photograph frame), free preventative NRT (Nicorette Icy White 2mg, 30 pieces), plus advice and support to use NRT or e-cigarettes for relapse prevention.

I.) SMS or app notification tailored support – This will be triggered by the birth notification, delivering a programme of tailored relapse prevention messages. Messages start daily, with a diminishing schedule over 12 months. At regular intervals participants are asked to confirm smoking status, and either then stay on the 'smokefree' or 'lapse' track of tailored messages. There is the option to opt out by texting 'stop' at any time.

3. **Postnatal period and beyond**

J.) At home/virtual postnatal visit with a health visitor, associated practitioner or BabyBreathe intervention trained researcher at around 10-14 days postpartum, when care is handed over from midwives to health visitors. At this visit, the health visitor will discuss smoking status, give positive praise, offer relapse prevention support, affirm that the BabyBreathe box has been received, and discuss contents of the BabyBreathe box, and text/app message use.

K.) Reiteration of support from health visitors or associated practitioners, up to 12 months postpartum – all subsequent postpartum routine health visitor appointments for the duration of the study will be undertaken by the same health visitor or health visiting team member where possible, , to assure continuity of care, which would be anticipated as part of usual practice. Positive praise will be offered for sustained smoking abstinence and the importance of relapse prevention will be emphasised. Participants will be encouraged to continue to engage, or to re-engage, with the full suite of BabyBreathe resources. Cessation support (referral) will be offered to partners where necessary and appropriate. For those who relapse, referral for cessation support will also be offered.

See Figure 2 for examples of the components of the BabyBreathe intervention.

Insert Figure 2 here

Outcomes

See Table 1 for participant timeline of interventions and assessments.

Table 1: BABYBREATHE PARTICIPANT TIMELINE

Schedule of enrolment, interventions, and assessments.

Antenatal				Postnatal				
**Screening (From 8 weeks to birth)***	**Confirm Eligibility (From 26 weeks)**	**Baseline (From confirmation of eligibility)**	**Health Visit (From randomisation up to birth)**	**Postnatal within 7 days**	**Health Visit (10-14 days Postpartum)**	**Health Visits (All subsequent routine)**	**6 month follow up**	**12 month follow up**
X								

For peer review only - http://bmjopen.bmj.com/site/about/guidelines.xhtml

X								
X								
	X							X
		X						
		X					X	X
		X					X	X
		X					X	X
		X					X	X
		X					X	X
		X					X	X
		X					X	X
		X					X	X
		X					X	X
		X					X	X
		X						
			X	X	X	X	X	X
				X				
								X
								X

*Potential participants will be variously identified during pregnancy: identified at pregnancy booking visit, at dating scans at (8-12 weeks) and/or 20 week scan, by screening records, or at routine care pregnancy appointments occurring anytime between start of pregnancy and 26 weeks of pregnancy, and by self-referral through responding to adverts. These practices will vary and due to pandemic related restrictions of appointments that may happen remotely. Those missed can be eligible for recruitment following 26 weeks of pregnancy, until the end of pregnancy

Primary outcome:

The primary effectiveness outcome is self-reported continuous smoking abstinence, from birth, biochemically validated by CO monitoring at 12 months postpartum, with cut off of less than 8ppm (i.e. a reading of 7ppm or less) for those who are not pregnant, or with a cut off of less than 4ppm if they are pregnant at this time point, according to the Russell standard (27). Adapting the Russell standard for the postpartum population, we will grant a period of 'grace', allowing up to 5 smoking lapses (a one off instance of smoking) between the birth of the baby and the 12 month follow-up, before the outcome is counted as relapse. Participants will provide CO readings electronically, using the iCO monitor (Bedfont) and study app (iCOBabyBreathe) provided at baseline. Participants will provide the REDCap database with two CO readings at entry and follow up. The highest of the readings will be recorded. Where CO readings take place in person as part of standard care, or research visits, or when participants request help with taking a CO reading, these readings may be used.

Secondary outcomes:

Secondary outcomes (Table 1) measured at 6 and 12 months postpartum by online self-report, or researcher follow-up, include self-reported point prevalence abstinence, self-reported time to

For peer review only - http://bmjopen.bmj.com/site/about/guidelines.xhtml

For peer review only

relapse, participant-reported partner smoking status, self-efficacy (single item, self-report), Edinburgh postnatal depression scale(27), behavioural support use (e.g. support from a stop smoking service), nicotine product use, perceived stress(28), AUDIT-C(29), HRQoL using the EQ-5D-5L(30). Infant health outcomes (e.g. minor infections requiring GP visits and more serious ill health requiring hospitalisation), mother and infant health resource use and cost-effectiveness will be measured at 12 months postpartum using a combination of GP patient records and participant self-report.

Sample size

If the primary outcome (continued smoking abstinence) occurs in 25% of the control group (based on an estimated relapse rate of 75%(13)) compared with 35% of the intervention group, then we estimate that we will need 880 participants (440 per group, 220 per recruitment hub) to have 90% power to detect this 10% between group difference at the 5% level of significance. We estimate this difference between control and intervention groups is realistic based on recent trials(31). Loss to follow up or withdrawal is not considered within the sample size calculation, as all those lost to follow up will be counted as returned to smoking, as is the usual convention in smoking cessation trials(32).

Retention

To maximise retention and minimise loss to follow up, we will make the following efforts to retain contact with study participants. There will be one text/email reminder sent if links to questionnaires/forms are not followed by participants. If participants have not followed the initial links or reminders, then study researchers will contact up to five times to offer support, and collect self-report data where possible. Outcome data collection at 6 and 12 months flexibly includes electronic, phone, post and face-to-face options. Participants will also be offered reimbursement for their time (£15 shopping voucher) on completion of 12-month follow-up.

Data analysis

We will use descriptive statistics to present the baseline characteristics of the two study groups**.** We will use chi-squared tests to compare follow-up rates between the study groups, to establish whether there is differential drop out. Analysis of smoking status will be based on the intention-to-treat principle by analysing individuals according to the treatment they were allocated to regardless of compliance. Individuals for whom we do not have the primary outcome data will be assumed to have returned to smoking. Analysis of the primary outcome will be based on a logistic regression model, adjusting for the stratification variables used in the randomisation algorithm. Secondary analysis will adjust for factors known to be predictive of relapse which will be agreed with the TSC and added to the Statistical analysis plan (SAP) prior to analysis. Secondary outcomes will be analysed in a similar fashion using a general linear model. Missing data patterns will be examined, and if appropriate, multiple imputation will be undertaken. The SAP is pre-registered (On the open science framework) – see appendix 1. The analysis plan may include analysis suggested by the qualitative analysis, such as subgroup analysis or mediation analysis. Any analysis will be pre-specified before data lock and published in the SAP prior to any data analysis.

Economic evaluation

An economic analysis will be conducted as an integral part of the randomised controlled trial. The primary perspective will be the NHS and social care: however, we will also look at broader relevant costs such as purchase of nicotine replacement therapies. All resources required to provide

For peer review only - http://bmjopen.bmj.com/site/about/guidelines.xhtml

BabyBreathe will be recorded: these will include staff time; equipment; consumables; required staff training; and any other relevant costs. For staff time to carry out specific tasks to provide BabyBreathe a variety of methods to obtain these data will be explored: these would include trial records on relevant expenditure and expert opinion. Health care resource use will be obtained from two sources. Firstly, we will include a modified CSRI to obtain data by participant self-report at the 12 month follow-up. This will cover the following: maternal antenatal hospital admissions; details of delivery, including mode of delivery and length of stay; and infant NICU admissions. Contacts with GP and practice nurses, contact with other primary care practitioners and referral to secondary care will also be collected as well as smoking cessation related expenditure. Additionally, where feasible we will obtain data from patient notes and GP records All resources identified during the study will be valued using appropriate local and national unit cost data.

The main outcome measure used in the economic analysis will be the study's primary outcome measure, continuous post-partum smoking abstinence. This will form a cost-effectiveness study looking at cost per additional sustained abstainer. Additionally, we will use EQ-5D-5L(30) values obtained from participants to undertake a cost utility analysis (i.e. cost per QALY) estimating quality adjusted life years (QALYs), obtained at baseline, 6 and 12 months post-partum. EQ-5D-5L questionnaires will be valued using the most appropriate scoring algorithm at time of analysis. Currently, this would be the UK mapped scores(33). Cost and effectiveness data will be estimated using regression based methods to allow for differences in baseline characteristics between groups. Non parametric bootstrapping will be used to allow for uncertainty and this will also be used to construct a cost-effectiveness acceptability curve (CEAC), which shows how likely the intervention is to be cost-effective at different monetary values of the effectiveness measures. A health economics analysis plan will be agreed and published on the OSF before any analysis of health economics data.

Process evaluation

Both qualitative and quantitative data will be collected by the study research team to assess implementation of the intervention, mechanisms of impact and contextual influences, as per MRC guidance(34,35) (Table 2).

Table 2: Components of the BabyBreathe mixed methods process evaluation

Aims	Process evaluation component (Moore et al., BMJ 2015))	Method of data collection
Assess fidelity of BabyBreathe training	**Implementation**<br>Training	Questionnaires before and after training
Assess fidelity of intervention contacts	**Implementation** (intervention contacts)<br>Dose, reach, engagement	Log of visits by health visitor, health visiting practitioner, or researcher (participant level)<br>Audio-recordings of 10% of contacts (antenatal and post-natal)<br>Qualitative interviews (health visitors, members of the health visiting team, or researcher – fidelity of delivery)<br>Qualitative interviews (participants and partners – engagement with visits and type

For peer review only - http://bmjopen.bmj.com/site/about/guidelines.xhtml

		of staff delivering the intervention)
Assess fidelity/engagement with the website and app	**Implementation** (website/app)<br>Dose, reach, engagement	Website and app data (number of log ins, total time in use)<br>Social support group threads<br>Number of texts received<br>Discontinuation of text/app notifications<br>Qualitative interviews (participants)
Assess contamination between trial arms	**Implementation** (intervention contacts)<br>Contamination	Recorded by trial research teams at each recruitment hub<br>Qualitative interviews (health visitors, members of the health visiting team, or researchers)<br>Health visitor feedback groups
Assess protocol modifications	**Implementation** (intervention contacts, website/app)<br>Fidelity, adaptations (intended and unintended/unforeseen; positive adaptations or drift)	Recorded by trial research teams at each recruitment hub<br>Qualitative interviews (health visitors, members of the health visiting team, or researchers)<br>Health visitor, member of the health visiting team, and researcher feedback groups
Assess how the intervention worked	**Mechanisms of impact:** hypothesised and unintended/unexpected pathways	Engagement data across recruitment hubs (visits)<br>Engagement with website and app<br>Engagement with text support<br>Use of Babybreathe box components (self-report, qualitative interviews and health visitor interviews)<br>Qualitative interviews (participants)
Assess contextual influences on implementation and mechanisms of impact	**Context:** contextual influences, e.g., participant/health visitor characteristics and geographical region, on implementation and mechanisms of impact	Qualitative interviews with health visitors, members of the health visiting team, or researchers, and participants
Assess the impact of the Covid-19 pandemic on intervention delivery and participant efforts to remain quit/stop smoking (partner)	**Implementation processes** (health visitor perspective)<br>Fidelity<br>Adaptions (by health visitors, members of the health visiting team, or researchers)<br>**Context**<br>Covid-19 pandemic response, e.g., restrictions, (partial) lockdowns.<br>**Mechanisms of impact**<br>Mediators	Qualitative interviews with health visitors, members of the health visiting team, or researchers, and participants

For peer review only - http://bmjopen.bmj.com/site/about/guidelines.xhtml

Fidelity of intervention delivery (*implementation*) and participant engagement with the health visitor visits and website/app will be assessed quantitatively through logs of visits, data analytics for website/app usage (the number of times that systems are logged on to, which resources are accessed, the time of engagement, the delivery of support messages via notifications and text messages, the time of any disengagement, discontinuation of SMS or app notifications, and self-reported engagement (as per (36)). Qualitative analysis will be undertaken of social support group threads, for which consent will have been sought on recruitment to the study; and audio-recordings (health visitors, practitioners or BabyBreathe researchers will be asked to record 10% of visits (≤10-mins intervention only), ante-natal as well as post-natal) and interviews with health visitors (n=12) and a qualitative interview subsample of participants and partners (n=40). Potential contamination between trial arms and protocol modifications will be assessed through qualitative interviews with health visitors and regular reporting by trial research teams. We will assess whether any identifiable modifications were planned adaptations to fit context, or unforeseen, and report our findings according to FRAME, an established framework(37). To illuminate possible mechanisms of action, a combined analysis of qualitative participant interview data, audio-recordings (e.g., intervention duration, delivery of behaviour change techniques) and quantitative engagement data across recruitment hubs will assess which components of the intervention were perceived to be particularly effective, for which people, in which contexts.

Data management

In view of the nature of the population (who are all expected to have one or more pregnancy related hospitalisation and primary care attendances which will be recorded in medical records); the intervention (which is not a medicinal product with the exception of Nicotine Replacement Therapy (gum) included in the BabyBreathe Box; and the trial primary and secondary outcomes, we do not intend to collect any additional safety endpoints.

BabyBreathe Trial Team members review the trial database to generate reports and review data entry. The essential trial issues, events and outputs, including defined key data points, are discussed by the trial team on a weekly basis and with relevant committees when necessary. A data sharing statement is included in the trial registry entry.

Ethics and dissemination:

Full REC and HRA approval has been granted (REC reference: 21/NW/0017, IRAS Project ID: 291746, protocol version 7 dated 04.05.2022. Participants provide electronic consent to take part, and rights of refusal to participate, or requests of withdrawal will be respected.

The results of the trial will be disseminated in open access journals, regardless of the direction of effect. The full protocol, statistical analysis plan, qualitative and health economics analysis plans, and anonymised datasets will be published in an online open access repository.

Current study status: Recruitment opened in April 2021 and the first participant was randomised in September 2021. Recruitment is expected to take 24 months, with results expected to be published following final follow up in late 2024 or early 2025.

Acknowledgements:

We appreciate the support of the study sponsor, Norfolk & Waveney CCG. The study is led by researchers at the University of East Anglia (UEA) and managed by the Norwich Clinical Trials Unit (NCTU) at UEA. Our thanks go to all organisations involved in recruitment and in supporting delivery of the intervention, particularly health visiting services and midwifery services. We would also like to

For peer review only - http://bmjopen.bmj.com/site/about/guidelines.xhtml

thank PPI contributors. We acknowledge the contribution of the researchers at sites who underwent training to deliver the intervention. Oversight provided by the independent Trial Steering Committee, comprising: Lion Shahab (Chair), Jo Leonardi-Bee, Jinshou Li, Siobhan Paul (PPI rep), Michael Twigg (Host rep) and Hilary Wareing. Sue Cooper, Graham Horne (sponsor rep), John Waldron, Libby White (PPI rep) and Julie Wright (funder rep); and Independent Data Monitoring committee: Paul Aveyard (Chair), Emma Beard and Karen Whittaker (terms of reference available upon request).

Author contributions: CN & TJB conceived the study idea, and drafted the manuscript. CN, TJB, WH, DS, MU & FN developed the intervention. GH is the trial manager and MM in the senior trial manager. AC is the trial statistician, DT is the health economist. LB, SD, VG, MU & TH are site PIs. RH provides public health academic trials expertise and DS provides computer science oversight.

Declaration of interests: All authors confirm that they have no competing financial or other potential conflicts of interest to declare.

Funding statement: This study is supported by the National Institute for Health and Care Research (NIHR) Public Health Research programme (project reference NIHR129074). The views expressed are those of the author(s) and not necessarily those of the NIHR or the Department of Health and Social Care.

REFERENCES

1. Patnode CD, Henderson JT, Coppola EL, Melnikow J, Durbin S, Thomas RG. Interventions for Tobacco Cessation in Adults, Including Pregnant Persons: Updated Evidence Report and Systematic Review for the US Preventive Services Task Force. JAMA. 2021 Jan 19;325(3):280–98.

2. Claire R, Chamberlain C, Davey M, Cooper SE, Berlin I, Leonardi-Bee J, et al. Pharmacological interventions for promoting smoking cessation during pregnancy. Cochrane Database Syst Rev. 2020 Mar 4;2020(3):CD010078.

3. Adult smoking habits in Great Britain - Office for National Statistics [Internet]. [cited 2019 Aug 7]. Available from: https://www.ons.gov.uk/peoplepopulationandcommunity/healthandsocialcare/drugusealcoholandsmoking/datasets/adultsmokinghabitsingreatbritain

4. Lumley J, Chamberlain C, Dowswell T, Oliver S, Oakley L, Watson L. Interventions for promoting smoking cessation during pregnancy. Cochrane Database Syst Rev. 2009 Jul 8;(3):CD001055.

5. Letourneau AR, Sonja B, Mazure CM, O'Malley SS, James D, Colson ER. Timing and predictors of postpartum return to smoking in a group of inner-city women: an exploratory pilot study. Birth. 2007 Sep;34(3):245–52.

6. Flemming K, Graham H, Heirs M, Fox D, Sowden A. Smoking in pregnancy: a systematic review of qualitative research of women who commence pregnancy as smokers. J Adv Nurs. 2013 May;69(5):1023–36.

For peer review only - http://bmjopen.bmj.com/site/about/guidelines.xhtml

7. Jones M, Lewis S, Parrott S, Wormall S, Coleman T. Re-starting smoking in the postpartum period after receiving a smoking cessation intervention: a systematic review. Addiction. 2016;111(6):981–90.

8. Livingstone-Banks J, Norris E, Hartmann-Boyce J, West R, Jarvis M, Hajek P. Relapse prevention interventions for smoking cessation. Cochrane Database Syst Rev. 2019 13;2:CD003999.

9. NHS England » NHS Long Term Plan [Internet]. [cited 2019 Mar 6]. Available from: https://www.england.nhs.uk/long-term-plan/

10. Smokefree 2030 – Smokefree Action Coalition [Internet]. [cited 2022 Jan 19]. Available from: https://smokefreeaction.org.uk/smokefree2030/

11. Chamberlain C, O'Mara-Eves A, Porter J, Coleman T, Perlen SM, Thomas J, McKenzie JE. Psychosocial interventions for supporting women to stop smoking in pregnancy. 2017, Issue 2. Art. No.: CD001055. [Internet]. Cochrane Database of Systematic Reviews; 2017. Available from: 10.1002/14651858.CD001055.pub5.

12. Notley C, Brown TJ, Bauld L, Hardeman W, Holland R, Naughton F, et al. Development of a Complex Intervention for the Maintenance of Postpartum Smoking Abstinence: Process for Defining Evidence-Based Intervention. International Journal of Environmental Research and Public Health. 2019 Jan;16(11):1968.

13. Hajek P, Stead LF, West R, Jarvis M, Hartmann-Boyce J, Lancaster T. Relapse prevention interventions for smoking cessation. Cochrane Database Syst Rev. 2013 Aug 20;(8):CD003999.

14. Doll R, Peto R, Boreham J, Sutherland I. Mortality in relation to smoking: 50 years' observations on male British doctors. BMJ. 2004 Jun 24;328(7455):1519.

15. Sims M, Tomkins S, Judge K, Taylor G, Jarvis MJ, Gilmore A. Trends in and predictors of second-hand smoke exposure indexed by cotinine in children in England from 1996 to 2006. Addiction. 2010 Mar;105(3):543–53.

16. DiFranza JR, Aligne CA, Weitzman M. Prenatal and postnatal environmental tobacco smoke exposure and children's health. Pediatrics. 2004 Apr;113(4 Suppl):1007–15.

17. RCP London [Internet]. [cited 2018 Mar 20]. Passive smoking and children. Available from: https://shop.rcplondon.ac.uk/products/passive-smoking-and-children

18. Leonardi-Bee J, Jere ML, Britton J. Exposure to parental and sibling smoking and the risk of smoking uptake in childhood and adolescence: a systematic review and meta-analysis. Thorax. 2011 Oct;66(10):847–55.

19. Godfrey C, Pickett KE, Parrot S et al. Estimating the costs to the NHS of smoking in pregnancy for pregnant women and infants. Department of Health Sciences, The University of York; 2010.

For peer review only - http://bmjopen.bmj.com/site/about/guidelines.xhtml

20. Royal College of Physicians. Hiding in plain sight. Treating tobacco dependency in the NHS. A report by the Tobacco Advisory Group of the Royal College of Physicians. 2018.

21. Brown TJ, Hardeman W, Bauld L, Holland R, Maskrey V, Naughton F, et al. A systematic review of behaviour change techniques within interventions to prevent return to smoking postpartum. Addict Behav. 2019 May;92:236–43.

22. Chan AW, Tetzlaff JM, Altman DG, Laupacis A, Gøtzsche PC, Krleža-Jerić K, et al. SPIRIT 2013 statement: defining standard protocol items for clinical trials. Ann Intern Med. 2013 Feb 5;158(3):200–7.

23. Hoffmann TC, Glasziou PP, Boutron I, Milne R, Perera R, Moher D, et al. Better reporting of interventions: template for intervention description and replication (TIDieR) checklist and guide. BMJ. 2014 Mar 7;348:g1687.

24. Bailey BA. Using expired air carbon monoxide to determine smoking status during pregnancy: preliminary identification of an appropriately sensitive and specific cut-point. Addict Behav. 2013 Oct;38(10):2547–50.

25. Harris PA, Taylor R, Thielke R, Payne J, Gonzalez N, Conde JG. Research electronic data capture (REDCap)—A metadata-driven methodology and workflow process for providing translational research informatics support. Journal of Biomedical Informatics. 2009 Apr 1;42(2):377–81.

26. Michie S, van Stralen MM, West R. The behaviour change wheel: A new method for characterising and designing behaviour change interventions. Implementation Science. 2011 Apr 23;6(1):42.

27. Bunevicius A, Kusminskas L, Pop VJ, Pedersen CA, Bunevicius R. Screening for antenatal depression with the Edinburgh Depression Scale. J Psychosom Obstet Gynaecol. 2009 Dec;30(4):238–43.

28. Cohen S, Kamarck T, Mermelstein R. A Global Measure of Perceived Stress. Journal of Health and Social Behavior. 1983;24(4):385–96.

29. Bush K, Kivlahan DR, McDonell MB, Fihn SD, Bradley KA. The AUDIT alcohol consumption questions (AUDIT-C): an effective brief screening test for problem drinking. Ambulatory Care Quality Improvement Project (ACQUIP). Alcohol Use Disorders Identification Test. Arch Intern Med. 1998 Sep 14;158(16):1789–95.

30. EQ-5D-5L – EQ-5D [Internet]. [cited 2020 Nov 8]. Available from: https://euroqol.org/eq-5d-instruments/eq-5d-5l-about/

31. Pollak KI, Fish LJ, Lyna P, Peterson BL, Myers ER, Gao X, et al. Efficacy of a Nurse-Delivered Intervention to Prevent and Delay Postpartum Return to Smoking: The Quit for Two Trial. Nicotine Tob Res. 2016 Oct 1;18(10):1960–6.

32. West R, Hajek P, Stead L, Stapleton J. Outcome criteria in smoking cessation trials: proposal for a common standard. Addiction. 2005 Mar;100(3):299–303.

For peer review only - http://bmjopen.bmj.com/site/about/guidelines.xhtml

33. van Hout B, Janssen MF, Feng YS, Kohlmann T, Busschbach J, Golicki D, et al. Interim scoring for the EQ-5D-5L: mapping the EQ-5D-5L to EQ-5D-3L value sets. Value Health. 2012 Aug;15(5):708–15.

34. Process evaluation in randomised controlled trials of complex interventions | The BMJ [Internet]. [cited 2018 Nov 12]. Available from: https://www.bmj.com/content/332/7538/413

35. Craig P, Dieppe P, Macintyre S, Michie S, Nazareth I, Petticrew M. Developing and evaluating complex interventions: the new Medical Research Council guidance. BMJ [Internet]. 2008 Sep 29;337. Available from: https://www.ncbi.nlm.nih.gov/pmc/articles/PMC2769032/

36. Groner J, French G, Ahijevych K, Wewers ME. Process Evaluation of a Nurse-Delivered Smoking Relapse Prevention Program for New Mothers. Journal of Community Health Nursing. 2005 Sep 1;22(3):157–67.

37. Wiltsey Stirman S, Baumann AA, Miller CJ. The FRAME: an expanded framework for reporting adaptations and modifications to evidence-based interventions. Implementation Science. 2019 Jun 6;14(1):58.

Figure legends:

Figure 1: Trial flow diagram

Figure 2: Examples of Babybreathe intervention components

For peer review only

For peer review only - http://bmjopen.bmj.com/site/about/guidelines.xhtml

Figure 1: Babybreathe Trial flow diagram

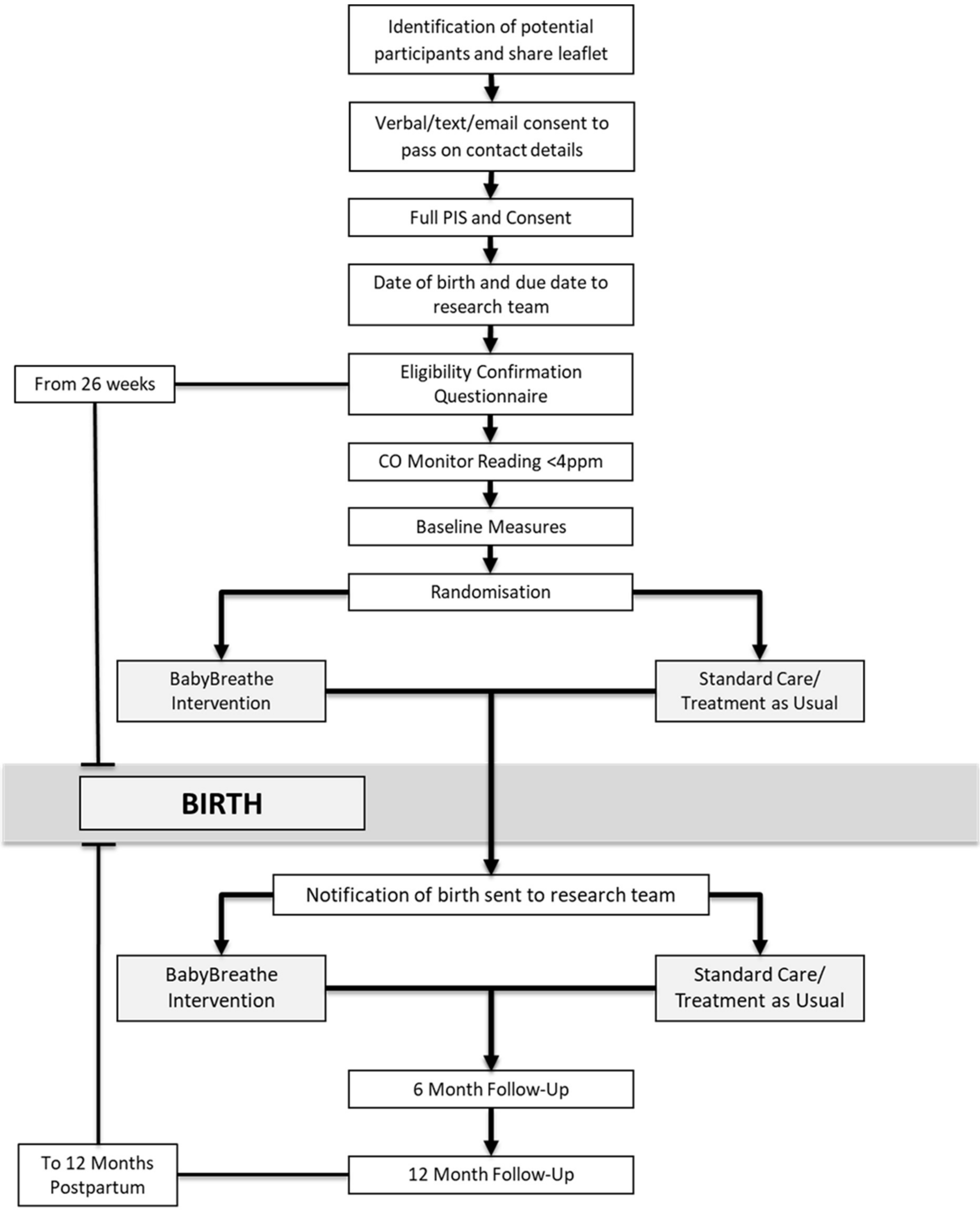

Figure 2: Examples of Babybreathe™ intervention components

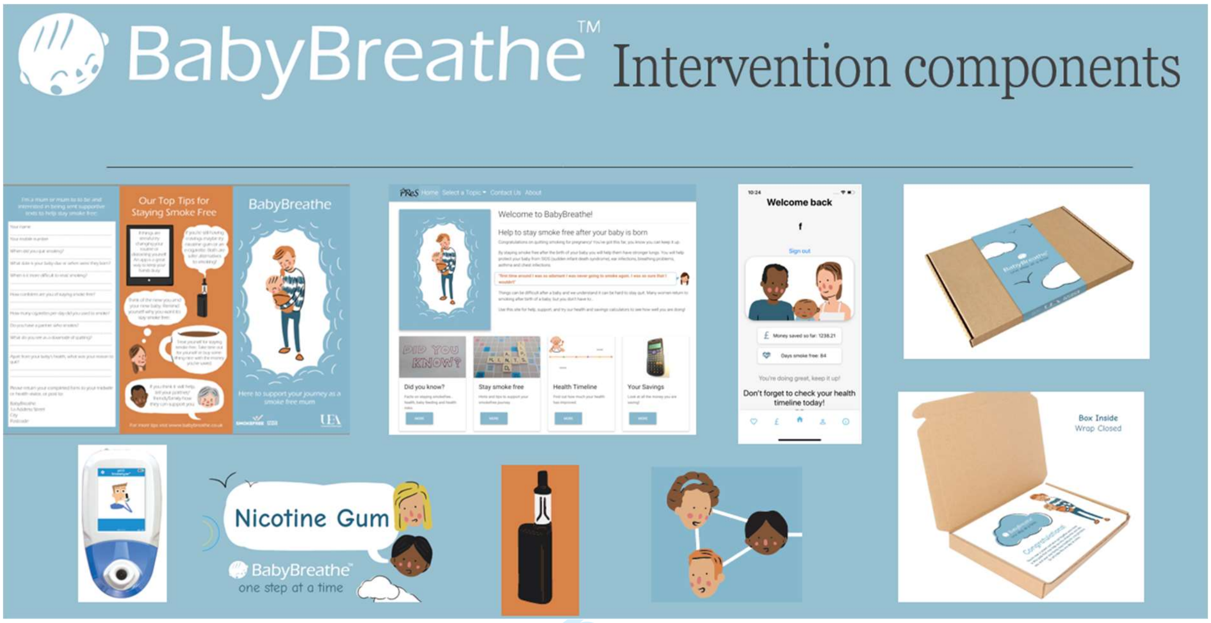

review only

TIDieR
Template for Intervention Description and Replication

The TIDieR (Template for Intervention Description and Replication) Checklist*:

Information to include when describing an intervention and the location of the information

Item number	Item	Where located ** Primary paper (page or appendix number)	Other † (details)
	**BRIEF NAME**		
**1.**	Provide the name or a phrase that describes the intervention.	__Title page__	'Babybreathe'
	**WHY**		
**2.**	Describe any rationale, theory, or goal of the elements essential to the intervention.	___page 3___	______________
	**WHAT**		
**3.**	Materials: Describe any physical or informational materials used in the intervention, including those provided to participants or used in intervention delivery or in training of intervention providers. Provide information on where the materials can be accessed (e.g. online appendix, URL).	Page 6, 7	______________
**4.**	Procedures: Describe each of the procedures, activities, and/or processes used in the intervention, including any enabling or support activities.	Page 6,7	______________
	**WHO PROVIDED**		
**5.**	For each category of intervention provider (e.g. psychologist, nursing assistant), describe their expertise, background and any specific training given.	__page 6, 7__	______________
	**HOW**		
**6.**	Describe the modes of delivery (e.g. face-to-face or by some other mechanism, such as internet or telephone) of the intervention and whether it was provided individually or in a group.	__page 6, 7__	______________
	**WHERE**		
**7.**	Describe the type(s) of location(s) where the intervention occurred, including any necessary infrastructure or relevant features.	___page 6, 7_	______________

For peer review only

	**WHEN and HOW MUCH**		
**8.**	Describe the number of times the intervention was delivered and over what period of time including the number of sessions, their schedule, and their duration, intensity or dose.	__page 7____	_____________
	**TAILORING**		
**9.**	If the intervention was planned to be personalised, titrated or adapted, then describe what, why, when, and how.	___page 6, 7__	_____________
	**MODIFICATIONS**		
**10.‡**	If the intervention was modified during the course of the study, describe the changes (what, why, when, and how).	_____N/A_______ ___	_____________
	**HOW WELL**		
**11.**	Planned: If intervention adherence or fidelity was assessed, describe how and by whom, and if any strategies were used to maintain or improve fidelity, describe them.	__ page 10,11	_____________
**12.‡**	Actual: If intervention adherence or fidelity was assessed, describe the extent to which the intervention was delivered as planned.	___N/A_______ ___	NA at protocol stage

** **Authors** - use N/A if an item is not applicable for the intervention being described. **Reviewers** – use '?' if information about the element is not reported/not sufficiently reported.

† If the information is not provided in the primary paper, give details of where this information is available. This may include locations such as a published protocol or other published papers (provide citation details) or a website (provide the URL).

‡ If completing the TIDieR checklist for a protocol, these items are not relevant to the protocol and cannot be described until the study is complete.

* We strongly recommend using this checklist in conjunction with the TIDieR guide (see *BMJ* 2014;348:g1687) which contains an explanation and elaboration for each item.

* The focus of TIDieR is on reporting details of the intervention elements (and where relevant, comparison elements) of a study. Other elements and methodological features of studies are covered by other reporting statements and checklists and have not been duplicated as part of the TIDieR checklist. When a **randomised trial** is being reported, the TIDieR checklist should be used in conjunction with the CONSORT statement (see www.consort-statement.org) as an extension of **Item 5 of the CONSORT 2010 Statement.** When a **clinical trial protocol** is being reported, the TIDieR checklist should be used in conjunction with the SPIRIT statement as an extension of **Item 11 of the SPIRIT 2013 Statement** (see www.spirit-statement.org). For alternate study designs, TIDieR can be used in conjunction with the appropriate checklist for that study design (see www.equator-network.org).

For peer review only - http://bmjopen.bmj.com/site/about/guidelines.xhtml

Babybreathe

A RANDOMISED CONTROLLED TRIAL OF A COMPLEX INTERVENTION TO PREVENT RETURN TO SMOKING IN WOMEN POSTPARTUM

For peer review only

Statistical Analysis Plan (SAP)

Version 1.0

04.06.2023

Name	Title	Signature	Date
Caitlin Notley	Chief Investigator	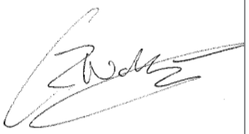	04.06.2023
Allan Clark	Statistician	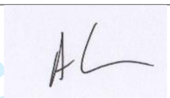	07.6.2023
Lucy Clark	Trial Manager	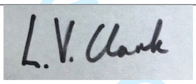	07.06.2023

SAP REVISION HISTORY

Document Name	Version No.	Reason for Revision	Effective Date

For peer review only - http://bmjopen.bmj.com/site/about/guidelines.xhtml

NORWICH CTU

1.0 Administrative Information

Sponsor : Norfolk and Norwich University Hospitals NHS Foundation Trust (NNUH)

Sponsor Reference : R207276

Funder : National Institute for Health Research – Health Technology Assessment

Funder Reference : NIHR129074

Trial Registration : ISRCTN70307341

IRAS: 291746

Chief Investigator : Caitlin Notley

Trial Statistician : Allan Clark

UKCRC Trials Unit : NCTU

Latest Protocol : Version 7.0

For peer review only

For peer review only - http://bmjopen.bmj.com/site/about/guidelines.xhtml

NORWICH CTU

2.0 Introduction

2.1 Background and Rationale

This is provided in section 4.1 of the protocol.

2.2 Objectives

The overall trial objectives are provided in section 4.2 of the protocol, however this SAP covers the following

1. To run an internal pilot study, with clear stop/go trial embedded criteria, primarily to test recruitment systems.

2. To definitively test the real-world effectiveness of BabyBreathe in comparison with usual care, by comparing smoking abstinence rates at 12 months postpartum between trial groups.

3.0 Study Methods

3.1 Trial Design

A two-group, multi-centre, pragmatic, individually randomised, controlled trial with an internal pilot, and including economic evaluation and process evaluation.

Intervention: Intervention participants will receive usual care plus the BabyBreathe package of support. The BabyBreathe intervention is informed by the Capability Opportunity Motivation-Behaviour (COM-B) model and Behaviour Change Wheel (21), with full consideration of postpartum women's context-specific concerns. The intervention is a package of support designed to be delivered at low cost alongside usual care and existing healthcare services. All intervention components have been developed and initially tested in our preliminary work with women and partners (MRC MR/PO16944/1) (9). The intervention comprises three main stages:

1. Antenatal support up to birth:

A.) BabyBreathe™ relapse prevention leaflet.

B.) Partner/Friend/Relative relapse prevention leaflet – content has been designed to encourage partners/friends/relatives to support women to stay smoke free after delivery, and to promote active cessation for partners/friends/relatives themselves, where needed.

C.) Brief advice from a health visitor, heath visiting team member practitioner or member of the research team trained to deliver the intervention (in-person, or remote). This advice is standardised and scripted following a protocol, with tailored options including positive praise for achieving smoking abstinence, and brief advice about the importance of staying smoke-free. Active signposting to the BabyBreathe digital /remote elements are included in this discussion.

D.) Electronic carbon monoxide testing – women are given an iCO monitor (Bedfont) for individual use. Intervention women will be prompted to use the iCO monitor to self-monitor CO levels at any time during the study (control participants will be restricted to use at baseline and study end).

For peer review only - http://bmjopen.bmj.com/site/about/guidelines.xhtml

NORWICH CTU

E.) BabyBreathe website and app – these resources have been specifically developed and operate on both android and iOS (iPhone) systems. The website and app can be accessed using a unique code by women for free. Women may input details such as the date they quit smoking, their estimated delivery date (EDD), and may access self-help support, health information and advice, including innovative motivational tools, such as a health calendar, and a closed online social support group, in preparation for entering the immediate postpartum period.

F.) At each subsequent health visitor contact (in-person, or remote), support for maintaining smoking abstinence will be briefly reiterated alongside usual care, where possible.

G.) Usual care provision (as delivered in any given site area) will continue also to be provided to the intervention group.

2. <u>Immediate postnatal period</u>

H.) BabyBreathe box – once the site team are alerted about the birth and input these data into the REDCap database, the central trial team will post out a BabyBreathe box. This is a physical box, designed to fit through a letter-box. The box includes self-incentives (e.g. reward chart, journal, photograph frame), free preventative NRT (Nicorette Icy White 2mg), plus advice and support to use NRT or e-cigarettes for relapse prevention.

I.) SMS or app notification tailored support – This will be triggered by the birth notification, delivering a programme of tailored relapse prevention messages that draw on data initially inputted by the user.

3. <u>Postnatal period and beyond</u>

J.) At home/virtual postnatal visit with a health visitor, associated practitioner or BabyBreathe intervention trained researcher at around 10-14 days postpartum, when full? care of women and babies is handed over from midwives to health visitors. At this visit, the health visitor will discuss smoking status, give positive praise, offer relapse prevention support, affirm that the BabyBreathe box has been received, and discuss contents of the BabyBreathe box, and text/app message use.

K.) Reiteration of support from health visitors or associated practitioners, up to 12 months postpartum – all subsequent postpartum routine health visitor appointments for the duration of the study will be undertaken by the same health visitor, health visiting team member, to assure continuity of care, which would be anticipated as part of usual practice. Positive praise will be offered for sustained smoking abstinence and the importance of relapse prevention will be emphasised. Women will be encouraged to continue to engage, or to re-engage, with the full suite of BabyBreathe resources. Cessation support (referral) will be offered to partners where necessary and appropriate. For women who relapse, referral for cessation support will also be offered.

Control:

Control participants will receive usual antenatal and postnatal care as per the NHS maternity care pathway (i.e. no routine relapse prevention support). Usual care varies across the UK and sites have been purposively selected to reflect the variations in routine care. Commonalities in usual care across all the sites include pregnant women being routinely screened for smoking status by their midwife at their

For peer review only - http://bmjopen.bmj.com/site/about/guidelines.xhtml

NORWICH CTU

first antenatal booking appointment. If a woman reports that she is currently smoking, or she has a CO reading greater than 3ppm (i.e. a reading of 4ppm or more), she is automatically referred for stop smoking support via NHS commissioned stop smoking services (opt-out referral). Midwifery support focuses on abstinence in pregnancy and only includes brief advice about the opportunity to maintain abstinence postpartum and in the long-term. The usual care group will receive standard ante and postnatal care, as per mandated midwifery and health visitor contacts. These visits may be face-to-face appointments, or they may be remote video or telephone appointments due to the COVID-19 pandemic.

3.2 Allocation

Completion and submission of the baseline measures will trigger randomisation (see trial flow diagram, figure 1). Participants will be individually randomised 1:1 via a computerised randomisation system managed by Norwich Clinical Trials Unit, to ensure adequate sequence generation and allocation sequence concealment. The randomisation system will stratify by recruitment hub, partner smoking status (partner smoking and partner non-smoking/no partner) and time of quit (before or during pregnancy), as factors that are likely to predict relapse.

3.3 Sample Size

This is provided in section 5.8 of the protocol but is repeated below.

If the primary outcome (continued smoking abstinence) occurs in 25% of the control group (based on an estimated relapse rate of 75% (10)) compared with 35% of the intervention group, then we estimate that we will need 880 participants (440 per group, 220 per recruitment hub) to have 90% power to detect this 10% between group difference at the 5% level of significance. We estimate this difference between control and intervention groups is realistic based on recent trials (27). Loss to follow up or withdrawal is not considered within the sample size calculation, as all those lost to follow up will be counted as returned to smoking, as is the usual convention in smoking cessation trials (ref). From national data, we estimate that approximately 22% of women will be smoking in the 12 months prior to pregnancy.

3.4 Framework

This is a superiority framework comparing the intervention to the control.

For peer review only - http://bmjopen.bmj.com/site/about/guidelines.xhtml

3.5 Timing of outcome assessments

Table 1: BABYBREATHE PARTICIPANT TIMELINE

Schedule of enrolment, interventions, and assessments.

	**Antenatal**					**Postnatal**				
	**Screening (From 8 weeks to birth)***	**Confirm Eligibility (From 26 weeks)**	**Baseline (From confirmation of eligibility)**	**Health Visit (From randomisation up to birth)**		**Postnatal within 7 days**	**Health Visit (10-14 days Postpartum)**	**Health Visits (All subsequent routine)**	**6 month follow up**	**12 month follow up**
Eligibility	X				Birth					
Consent to be contacted	X									
Link to PIS and Consent	X									
iCO reading to confirm eligibility		X								X
Demographics			X							
Smoking Status (SR)			X						X	X
Breastfeeding intention (ref)			X						X	X
Relapse Predictors			X						X	X
Self-Efficacy (SR)			X						X	X
Edinburgh Depression Scale (22)			X						X	X
Behavioural Support (SR)			X						X	X
Nicotine Product Use (SR)			X						X	X
AUDIT-C (23)			X						X	X
EQ-5D-5L (45)			X						X	X
Cohen 4 item Perceived stress scale (24)			X						X	X
Randomisation			X							
BabyBreathe Intervention				X		X	X	X	X	X
Birth Notification						X				
Healthcare Resource Use										X
Infant Health Outcomes										X

For peer review only - http://bmjopen.bmj.com/site/about/guidelines.xhtml

*Potential participants will be variously identified during pregnancy: identified at pregnancy booking visit, at dating scans at (8-12 weeks) and/or 20 week scan, by screening records, or at routine care pregnancy appointments occurring anytime between start of pregnancy and 26 weeks of pregnancy, and by self-referral through responding to adverts. These practices will vary and due to pandemic related restrictions of appointments that may happen remotely. Those missed can be eligible for recruitment following 26 weeks of pregnancy, until the end of pregnancy

3.6 Interim analyses and stopping guidance

There will be no formal interim analyses or stopping guidance. However, the trial does have progression criteria the end of the internal pilot stage after three months recruitment. These are listed below.

3.7 Timing of analyses

The internal pilot does not require the analysis of any outcomes or unblinded data so is not considered as 'analysis' for this SAP. The analysis will be done once the database is locked and the SAP approved once all of the outcome data has been collected.

4.0 Statistical Principles

4.1 Levels of statistical significance

A 5% level of significance and 95% confidence intervals will be used throughout.

4.2 Analysis populations

The ITT population is defined as the set of all randomized participants regardless of compliance. If participants are subsequently withdrawn from the study then there data will still be included and the missing data strategy detailed in the analysis section will be used. Individuals who are deemed to be post-randomisation exclusions will be excluded from the analysis.

A modified ITT population will exclude participants who had complications at birth. Analysis of this population will be made on the primary outcome only.

4.3 Treatment Adherence / received

Compliance of the intervention along with the treatment received will be reported as per the table below.

For peer review only - http://bmjopen.bmj.com/site/about/guidelines.xhtml

NORWICH CTU

Table 4.3.1: Compliance / treatment received

	Intervention delivery
**Antenatal support period**	n (%)
Relapse prevention leaflet.	
Partner/Friend/Relative relapse prevention leaflet	
Brief advice from a health visitor	
Electronic carbon monoxide testing given	
BabyBreathe website and app provided / accessed	
**Immediate postnatal period**	
BabyBreathe box sent	
SMS or app notification sent	
SMS or app opt out received	
**Postnatal period and beyond**	
At home/virtual postnatal visit with a health visitor	
Reiteration of support from health visitors	
Number of postpartum visits	
None	
One	
Two	
Three	
Four	
…	

4.4 Protocol deviations

Protocol deviations will be discussed at the TMG and will be reported as a list.

5.0 Trial Population

5.1 Screening data

The following data and tables will be reported from the screening data.

Table 5.1.1: Screening data by month of approaching patient

Month of screening	Number of patients approached	Number interested in participating	Number eligible* (pre-screening)	Number giving consent	Number eligible **	Number randomised

For peer review only - http://bmjopen.bmj.com/site/about/guidelines.xhtml

Table 5.1.2: Reasons for declining

	Number (% Of those declining participation)	Percentage of those approached

5.2 Eligibility

5.2.1 Participant Inclusion Criteria

1. Those who are pregnant who have stopped smoking completely in the 12 months prior to pregnancy, or at any time during pregnancy.
2. At 26 weeks gestation or any time following this up until birth, woman confirms having not smoked a single puff of a cigarette for at least four weeks.
3. Able to read and understand English.
4. Willing and able to give informed consent for participation in the study.
5. Expired carbon monoxide (CO) reading less than four parts per million (ppm)

5.2.2 Participant Exclusion Criteria

6. Under the age of 16

This will be reported as below.

Table 5.2.1: Reasons for ineligibility

	Frequency (%) (N=)
Pre-screening eligibility criteria met	
**Reason for exclusion (n=)**<br>Absences of inclusion criteria	
Those who are pregnantwho have stopped smoking completely in the 12 months prior to pregnancy, or at any time during pregnancy.	
At 26 weeks gestation or any time following this up until birth, participant confirms having not smoked a single puff of a cigarette for at least four weeks.	
Able to read and understand English.	

For peer review only - http://bmjopen.bmj.com/site/about/guidelines.xhtml

NORWICH CTU

Willing and able to give informed consent for participation in the study	
Expired carbon monoxide (CO) reading less than four parts per million (ppm)	
**Presence of exclusion criteria**	
Aged under 16	

5.3 Recruitment and participant flow

Table 5.3.1: Participant accrual (e.g. per time period, cumulative, if appropriate against predicted accrual in graphical form) for main participants (ITT population only)

Month of recruitment	Predicted	Actual	Cumulative Predicted	Cumulative Actual

Graph of predicted vs actual recruitment

A CONSORT diagram will also be produced.

5.4 Withdrawal information

Follow-up rates and reasons for withdrawal will be reported in the following tables.

Table 5.4.1: Follow-up

	ITT population	
	Control (n=)	Intervention (n=)
Lost to FU before birth		
Lost to FU month 0-6 post partum, n(%)		
Lost to FU month 7-12 post partum, n(%)		

For peer review only - http://bmjopen.bmj.com/site/about/guidelines.xhtml

NORWICH CTU

Table 5.4.2: Reasons for loss to follow-up.

	ITT population		ITT+ population	
	Control (n=)	Intervention (n=)	Control (n=)	Intervention (n=)
Reason lost to follow (month 0-6)				
Reason 1, n(%)				
Reason 2, n(%)				
….				
Reason lost to follow (month 6-12)				
Reason 1, n(%)				
Reason 2, n(%)				
….				

5.5 Baseline participant characteristics

The baseline characteristics will be summarized according to the table below.

Table 8: Baseline characteristics of trial participants

	ITT population (n=)	
	Control (n=)	Intervention (n=)
Age, mean (SD)		
Number of days into pregnancy when recruited, mean (SD)		
Number of days until due date, mean (SD)		
Days since last puff, mean (SD)		
When did you quit smoking		
Before pregnancy, n(%)		
During pregnancy, n(%)		
Partner smoking status		
No partner, n(%)		
Smoker, n(%)		
Never smoker, n(%)		
Ex smoker, n(%)		
Highest qualification		
None, n(%)		
GCSE, n(%)		
A-level, n(%)		
Degree, n(%)		

For peer review only - http://bmjopen.bmj.com/site/about/guidelines.xhtml

Norwich CTU

Missing, n(%)		
Ethnicity		
White, n(%)		
Mixed, n(%)		
Asian / Asian British, n(%)		
Black/African/Caribbean/Black British, n(%)		
Arab, n(%)		
Any other ethnic group, n(%)		
Missing, n(%)		
Marital status		
Single, n(%)		
Co-habiting, n(%)		
Civil partnership, n(%)		
Married, n(%)		
Divorced, n(%)		
Widowed, n(%)		
Missing, n(%)		
Confidence not continue to smoke until baby's first birthday		
Not at all confident		
Slightly confident		
Moderately confident		
Very confident		
Extremely confident		
Use of Nicotine replacement therapy		
Have you used any Nicotine Replacement Therapy (NRT) in the last week?		
Did you use an e-cigarette to help you stop smoking?		
have you used an e-cigarette in the last week?		
Did you use a heat-not-burn product to help you stopsmoking?		
Have you used a heat-not burn product in the last week?		
Did you receive any professional help with stopping smoking?		
Do you still receive help from this organisation to stay smoke free?		
Are you currently using any apps which help with quittingsmoking or staying quit from smoking?		
Edinburgh post natal depression scale, mean (SD)		
PSS4 score		

6.0 Analysis

For peer review only - http://bmjopen.bmj.com/site/about/guidelines.xhtml

NORWICH CTU

6.1 Outcome definitions

6.1.1 Primary Outcome

The primary effectiveness outcome is self-reported continuous postpartum smoking abstinence, biochemically validated by CO monitoring at 12 months postpartum, with cut off of less than 8ppm (i.e. a reading of 7ppm or less) for women who are not pregnant, or with a cut off of less than 4ppm if they are pregnant at this time point, according to the Russell standard (25,26). Adapting the Russell standard, we will grant a period of 'grace', allowing up to 5 smoking lapses between the birth of the baby and the 12 month follow-up, before the outcome is counted as relapse. Participants will provide CO readings electronically, using the iCO monitor (Bedfont) and study app (iCOBabyBreathe) provided at baseline. Participants will provide the REDCap database with two CO readings. Where CO readings take place in person as part of standard care, or research visits, these readings may be used.

This will be constructed from

- the question "Are you currently smoke free?" taking smoke free to be either
  - "Abstinent" with the answers " Yes I am smoke free - I have not smoked a cigarette in the last 12 months (not even a puff of a cigarette)" or " Yes I am smoke free currently but I have had between one and five lapses in the last 12 months (a cigarette, or puff of a cigarette)"; and
  - "relapse" to be either "Yes I am currently smoke free but I have had six or more lapses in the last 12 months (including relapse but quit again) " or "No, I am currently smoking tobacco"; and
- The CO readings will be classified as 'verified' if a reading of 7ppm or less; and 'not verified' if 8 or more. Missing values will be classified as 'not verified'

The primary outcome with be classified as "confirmed abstinent" if the participant's response is both "Abstinent" and "verified"; otherwise it will be classified "Not conformed abstinent".

6.1.2 Secondary Outcomes

The secondary outcomes are measured at 6 and 12 months postpartum and are:

- Self-reported abstinence defined as reporting less than 5 lapses in the last 6 months at 6 months and at 12 months. [1 or 2 in fu6_smoking_status] [1 or 2 in fu12_smoking_status]
- Self-reported time to relapse defined as time from birth of child until individual self-reported date when started smoking again [fu_smoking_again_de] for individuals who report that they are currently smoking at either 6 or 12 months.
- Relapse predictors
  - Partner smoking status [fu_partner_smoke_yn]
  - Self-reported breast feeding at 6 and 12 months (yes/no)
  - Self-reported duration of breast feeding

For peer review only - http://bmjopen.bmj.com/site/about/guidelines.xhtml

- Self-efficacy to remain smoke free measured using the question 'How confident are you that you will continue not to smoke at least until your baby's first birthday?' at 6 months and 'How confident are you that you will continue not to smoke?'
- Postnatal depression measured the Edinburgh postnatal depression scale. This has 10 items and is scored using a scale ranging from 0 to 30 with high value indicating greater chance of depression. The scoring guide used will be
- Behavioral support use measured using the question 'In the last 6 months have you received any professional help with stopping smoking, e.g. from NHS smokefree services?' This will be analysed separately for
  - Smoking-free services
  - GP advice
  - Digital smokefree services
- Nicotine product use measure as use of Nicotine Replacement Therapy in the last 6 months.
- Nicotine product use measure as use of Nicotine Replacement Therapy in the last 7 days
- Perceived stress will be measured using Cohen PSS4 scale. This consists 4 items each scored 0-4, with the total score ranging from 0 to 16 with higher values indicates more stress.
- AUDIT-C this is a questionnaire based on 3 questions [alcohol_frequency_fu], [alcohol_units_fu] and [alcohol_6ormore_units] each scored 0-4 and the total is scored 0-12 with higher values indicating higher chance of possible dependence.
- EQ-5D-5L
- Infant health outcomes measured by
  - Number of hospital admissions
  - Number of GP visits
  - Length of stay when giving birth
  - Neonatal unit admission or not.
- E-cig use
  - In last 6 months
  - Frequency of use in last 6 months
  - In last 7 days

6.1.3 Tertiary outcomes

None.

6.2 Analysis Methods

6.2.1 Primary outcome

The primary outcome will be compared between treatment groups using a log-binomial regression adjusting for the stratification variables in a 'minimally adjusted' model; if adjustment for additional variables is recommended from the TSC prior to analysis this will be detailed in this document. This will allow the estimation of the relative risk of abstinence between the two treatment groups. The risk difference will be estimated from this model using the predicted risk, those factors in the model which are categorical will be set at the value with the largest number of participants and the continuous

For peer review only - http://bmjopen.bmj.com/site/about/guidelines.xhtml

factors will be set at the mean value. Any individual with missing data will be assumed to have relapsed, in the event of the abstinence not being able to be confirmed biochemically it will also be assumed to have been in relapse.

Table 6.2.1: Summary for primary outcome (will be reported for the ITT and ITT+ populations)

	ITT population		Minimally adjusted (only for stratification variables)			Fully adjusted		
Outcome	Control (n=)	Intervention (n=)	Relative risk (95% CI)	p-value	Difference in risk (95%CI)	Relative risk (95% CI)	p-value	Difference in risk (95%CI)
12 Month abstinence	n(%)	n(%)						

6.2.2 Secondary outcomes

The following tables give the analysis for each outcome listed in section 6.1.2

Outcome	Self-reported continuous postpartum smoking abstinence. CO verified
Effect size	Relative risk
Primary Analysis model	Log-binomial regression adjusting for factors stratified in the randomisation.
Sensitivity analysis	Log-binomial regression adjusting for factors stratified in the randomisation and factors pre-specified by TMG before database lock.
Missing data	Missing data will be assumed to have relapsed into smoking.
Other comments	The effect size will also be estimated as the risk difference and 'number-need-to-treat' using an unadjusted model.<br>Biochemical validation of self-reported abstinence is the gold standard outcome assessment in smoking cessation/relapse prevention trials (27).

Outcome	6 month Self-reported continuous postpartum smoking abstinence
Effect size	Relative risk
Primary Analysis model	Log-binomial regression adjusting for factors stratified in the randomisation.

For peer review only - http://bmjopen.bmj.com/site/about/guidelines.xhtml

Norwich CTU

Sensitivity analysis	Log-binomial regression adjusting for factors stratified in the randomisation and factors pre-specified by TMG before database lock.
Missing data	Missing data will be assumed to have relapsed into smoking.
Other comments	The effect size will also be estimated as the risk difference and 'number-need-to-treat' using an unadjusted model.

Outcome	Self-reported time-to-relapse, defined as the time from randomisation until the date first smoked
Effect size	Hazard ratio
Primary Analysis model	Cox regression adjusting for factors stratified in the randomisation.
Sensitivity analysis	Cox regression adjusting for factors stratified in the randomisation and factors pre-specified by TMG before database lock.
Missing data	Individuals who drop-out will be assumed to have relapsed on the date of drop-out. Individuals who have not relapsed will be censored at the end of the study.
Other comments	Data will be presented graphically using a Kaplan-Meier Curve. Cox regression assumptions will be assessed visually using a plot of Schoenfeld residuals against follow-up time. If assumptions not met then other models adjustments to the model (treating variables as strata rather than covariates) will be attempted. If not possible then alternative modelling will be investigated.

Outcome	Participant reported partner smoking status at 6 and 12 months
Effect size	Relative risk
Primary Analysis model	Log-binomial regression adjusting for factors stratified in the randomisation.
Sensitivity analysis	Log-binomial regression adjusting for factors stratified in the randomisation and factors pre-specified by TMG before database lock.
Missing data	Missing data will be assumed to have relapsed into smoking.

For peer review only - http://bmjopen.bmj.com/site/about/guidelines.xhtml

NORWICH CTU

Other comments	Only to be analysed for individuals with a) the same partner status as at baseline; and b) the partner had quit by the date of randomisation

Outcome	Self-reported breastfeeding status
Effect size	Relative risk
Primary Analysis model	Log-binomial regression adjusting for factors stratified in the randomisation.
Sensitivity analysis	Log-binomial regression adjusting for factors stratified in the randomisation and factors pre-specified by TMG before database lock.
Missing data	Missing data will be imputed in a sensitivity analysis.
Other comments	Binary yes/no at 12 months

Outcome	Self-reported duration of breastfeeding (duration)
Effect size	Mean difference
Primary Analysis model	General linear model adjusting for factors stratified in the randomisation.
Sensitivity analysis	General linear model adjusting for factors stratified in the randomisation and factors pre-specified by TMG before database lock.
Missing data	Missing data will be imputed in a sensitivity analysis.
Other comments	

Outcome	Self-efficacy to remain smokefree
Effect size	Relative risk
Primary Analysis model	Log-binomial regression adjusting for factors stratified in the randomisation.
Sensitivity analysis	Log-binomial regression adjusting for factors stratified in the randomisation and factors pre-specified by TMG before database lock.
Missing data	Missing data will be imputed in a sensitivity analysis.
Other comments	Binary yes/no at 12 months

Outcome	Postpartum depression – Edinburgh postnatal depression questionnaire (39)
Effect size	Mean difference

For peer review only - http://bmjopen.bmj.com/site/about/guidelines.xhtml

Primary Analysis model	Linear regression adjusting for factors stratified in the randomisation.
Sensitivity analysis	Linear regression adjusting for factors stratified in the randomisation and factors pre-specified by TMG before database lock.
Missing data	Missing data will be assumed to have relapsed into smoking.
Other comments	Assumptions will be assessed via plots of residuals to check for normality. If not normally distributed a non-parametric bootstrap will be used or a Mann-Whitney test will be used.

Outcome	Access in-person smoke free services
Effect size	Relative risk
Primary Analysis model	Log-binomial regression adjusting for factors stratified in the randomisation.
Sensitivity analysis	Log-binomial regression adjusting for factors stratified in the randomisation and factors pre-specified by TMG before database lock.
Missing data	Missing data will be imputed in a sensitivity analysis.
Other comments	Binary yes/no at 12 months

Outcome	Access in-person GP advice for stop smoking
Effect size	Relative risk
Primary Analysis model	Log-binomial regression adjusting for factors stratified in the randomisation.
Sensitivity analysis	Log-binomial regression adjusting for factors stratified in the randomisation and factors pre-specified by TMG before database lock.
Missing data	Missing data will be imputed in a sensitivity analysis.
Other comments	Binary yes/no at 12 months

Outcome	Access digital smoke free services
Effect size	Relative risk
Primary Analysis model	Log-binomial regression adjusting for factors stratified in the randomisation.
Sensitivity analysis	Log-binomial regression adjusting for factors stratified in the randomisation and factors pre-specified by TMG before database lock.

For peer review only - http://bmjopen.bmj.com/site/about/guidelines.xhtml

NORWICH CTU

Missing data	Missing data will be imputed in a sensitivity analysis.
Other comments	Binary yes/no at 12 months

Outcome	Nicotine product use in last 6 months
Effect size	Relative risk
Primary Analysis model	Log-binomial regression adjusting for factors stratified in the randomisation.
Sensitivity analysis	Log-binomial regression adjusting for factors stratified in the randomisation and factors pre-specified by TMG before database lock.
Missing data	Missing data will be imputed in a sensitivity analysis.
Other comments	Binary yes/no at 12 months

Outcome	Nicotine product use in last 7 days
Effect size	Relative risk
Primary Analysis model	Log-binomial regression adjusting for factors stratified in the randomisation.
Sensitivity analysis	Log-binomial regression adjusting for factors stratified in the randomisation and factors pre-specified by TMG before database lock.
Missing data	Missing data will be imputed in a sensitivity analysis.
Other comments	Binary yes/no at 12 months

Outcome	Perceived Stress – Cohen perceived stress scale (41)
Effect size	Mean difference
Primary Analysis model	Linear regression adjusting for factors stratified in the randomisation.
Sensitivity analysis	Linear regression adjusting for factors stratified in the randomisation and factors pre-specified by TMG before database lock.
Missing data	Missing data will be assumed to have relapsed into smoking.
Other comments	Assumptions will be assessed via plots of residuals to check for normality. If not normally

For peer review only - http://bmjopen.bmj.com/site/about/guidelines.xhtml

NORWICH CTU

	distributed a non-parametric bootstrap will be used or a Mann-Whitney test will be used.

Outcome	Alcohol Use (AUDIT-C) (40)
Effect size	None.
Primary Analysis model	Mann-Whitney test.
Sensitivity analysis	None.
Missing data	Missing data will be imputed in a sensitivity analysis.
Other comments	This outcome is unlikely to be normally distributed so a non-parametric approach will be used.

Outcome	Quality of life EQ-5D-5L
Effect size	Mean difference
Primary Analysis model	Linear regression adjusting for factors stratified in the randomisation.
Sensitivity analysis	Linear regression adjusting for factors stratified in the randomisation and factors pre-specified by TMG before database lock.
Missing data	Missing data will be assumed to have relapsed into smoking.
Other comments	Assumptions will be assess via plots of residuals to check for normality. If not normally distributed a non-parametric bootstrap will be used

Outcome	Number of hospital admission for child in follow-up period
Effect size	Incident rate ratio
Primary Analysis model	Poisson regression adjusting for factors stratified in the randomisation.
Sensitivity analysis	Poisson regression adjusting for factors stratified in the randomisation and factors pre-specified by TMG before database lock.
Missing data	Missing data will be assumed to have relapsed into smoking.
Other comments	Assumptions will be assessed and if a negative binomial model fits the data better then it will be used.

For peer review only - http://bmjopen.bmj.com/site/about/guidelines.xhtml

NORWICH CTU

Outcome	Number of GP visits of child in follow-up period
Effect size	Incident rate ratio
Primary Analysis model	Poisson regression adjusting for factors stratified in the randomisation.
Sensitivity analysis	Poisson regression adjusting for factors stratified in the randomisation and factors pre-specified by TMG before database lock.
Missing data	Missing data will be assumed to have relapsed into smoking.
Other comments	Assumptions will be assessed and if a negative binomial model fits the data better then it will be used.

Outcome	Length of stay on birth
Effect size	Mean difference
Primary Analysis model	Linear regression adjusting for factors stratified in the randomisation.
Sensitivity analysis	Linear regression adjusting for factors stratified in the randomisation and factors pre-specified by TMG before database lock.
Missing data	Missing data will be assumed to have relapsed into smoking.
Other comments	Assumptions will be assessed via plots of residuals to check for normality. If not normally distributed a non-parametric bootstrap will be used

Outcome	Neonatal unit admission of child
Effect size	Relative risk
Primary Analysis model	Log-binomial regression adjusting for factors stratified in the randomisation.
Sensitivity analysis	Log-binomial regression adjusting for factors stratified in the randomisation and factors pre-specified by TMG before database lock.
Missing data	Missing data will be imputed in a sensitivity analysis.
Other comments	

Outcome	E-cigarette use in 6 months
Effect size	Relative risk
Primary Analysis model	Log-binomial regression adjusting for factors stratified in the randomisation.

For peer review only - http://bmjopen.bmj.com/site/about/guidelines.xhtml

Sensitivity analysis	Log-binomial regression adjusting for factors stratified in the randomisation and factors pre-specified by TMG before database lock.
Missing data	Missing data will be imputed in a sensitivity analysis.
Other comments	

Outcome	Frequency of using an 3-cigarette in past 6 months
Effect size	None.
Primary Analysis model	Mann-Whitney test.
Sensitivity analysis	None.
Missing data	Missing data will be imputed in a sensitivity analysis.
Other comments	This outcome is ordinally distributed so a non-parametric approach will be used.

For peer review only

Outcome	E-cigarette use in last week
Effect size	Relative risk
Primary Analysis model	Log-binomial regression adjusting for factors stratified in the randomisation.
Sensitivity analysis	Log-binomial regression adjusting for factors stratified in the randomisation and factors pre-specified by TMG before database lock.
Missing data	Missing data will be imputed in a sensitivity analysis.
Other comments	

Table 17: Secondary efficacy outcomes

	ITT population		Minimally adjusted		Fully adjusted	
Outcome	Control (n=)	Intervention (n=)	Effect size (95%CI)	p-value	Effect size (95%CI)	p-value

For peer review only - http://bmjopen.bmj.com/site/about/guidelines.xhtml

NORWICH CTU

6.3 Missing Data

As mentioned in the above our primary analysis will replace missing abstinence values with relapse and the analysis of the other endpoints will be of available case. A sensitivity analysis will be conducted using multiple imputation assuming that the data are not missing at random. Alternative assumptions will be investigated but it will not be known which approaches/assumption are appropriate until we have more data about the missingness pattern. However, a reasonable NMAR choice would be to assume that those with missing data have worse outcome than those without missing data.

6.4 Additional analyses

6.5 Safety analyses

Only descriptive analysis of the SAE and SE will be reported. These will simply be listed as per the tables below.

Table 6.5.1: serious adverse events (incl. event description, duration, relationship to intervention)

Group	Date of onset	Description	Date of resolution	Related to trial treatment	Randomised group

Table 6.5.2: adverse events, by event, severity, or if appropriate, by relationship to intervention (including duration of treatment exposure), body compartment/system:

Group	Date of onset	Description	Date of resolution	Related to trial treatment	Randomised group

6.5 Software

Stata 17.1 or higher will be used for the majority of the analyses, however alternative software may be used if required.

For peer review only - http://bmjopen.bmj.com/site/about/guidelines.xhtml

NORWICH CTU

7.0 References

Cohen, S., Kamarck, T., & Mermelstein, R. (1983). A global measure of perceived stress. Journal of Health and Social Behavior, 24, 385-396.

Cox, J.L., Holden, J.M., and Sagovsky, R. 1987.Detection of postnatal depression: Development of the 10-item Edinburgh Postnatal Depression Scale. British Journal of Psychiatry 150:782-786.

For peer review only

For peer review only - http://bmjopen.bmj.com/site/about/guidelines.xhtml

SPIRIT | CONSORT OUTCOMES Reporting Guidelines for Trial Outcomes

SPIRIT-Outcomes 2022 Checklist (for combined completion of SPIRIT 2013 and SPIRIT-Outcomes 2022 items)[a]

Section	Item No.	SPIRIT 2013 Item	SPIRIT-Outcomes 2022 item	Location Reported[b]
**Administrative information**				
Title	1	Descriptive title identifying the study design, population, interventions, and, if applicable, trial acronym	-	Pg 1
Trial registration	2a	Trial identifier and registry name. If not yet registered, name of intended registry	-	Pg 2
	2b	All items from the World Health Organization Trial Registration Data Set	-	N/A
Protocol version	3	Date and version identifier	-	Pg 2
Funding	4	Sources and types of financial, material, and other support	-	Pg 13
Roles and responsibilities	5a	Names, affiliations, and roles of protocol contributors	-	Pg 1, 13
	5b	Name and contact information for the trial sponsor	-	Pg 13
	5c	Role of study sponsor and funders, if any, in study design; collection, management, analysis, and interpretation of data; writing of the report; and the decision to submit the report for publication, including whether they will have ultimate authority over any of these activities	-	Pg 13
	5d	Composition, roles, and responsibilities of the coordinating centre, steering committee, endpoint adjudication committee, data management team, and other individuals or groups overseeing the trial, if applicable (see Item 21a for data monitoring committee)	-	Pg 13
**Introduction**				
Background and rationale	6a	Description of research question and justification for undertaking the trial, including summary of relevant studies (published and unpublished) examining benefits and harms for each intervention	-	Pg 2, 3
	6b	Explanation for choice of comparators	-	Pg 6
Objectives	7	Specific objectives or hypotheses	-	Pg 3,4

For peer review only

Section	Item No.	SPIRIT 2013 Item	SPIRIT-Outcomes 2022 item	Location Reported[b]
Trial design	8	Description of trial design including type of trial (eg, parallel group, crossover, factorial, single group), allocation ratio, and framework (eg, superiority, equivalence, noninferiority, exploratory)	-	Pg 4
**Methods: Participants, interventions, and outcomes**				
Study setting	9	Description of study settings (eg, community clinic, academic hospital) and list of countries where data will be collected. Reference to where list of study sites can be obtained	-	Pg 4
Eligibility criteria	10	Inclusion and exclusion criteria for participants. If applicable, eligibility criteria for study centres and individuals who will perform the interventions (eg, surgeons, psychotherapists)	-	Pg 4
Interventions	11a	Interventions for each group with sufficient detail to allow replication, including how and when they will be administered (for specific guidance see TIDieR checklist and guide)	-	Pg 6,7
	11b	Criteria for discontinuing or modifying allocated interventions for a given trial participant (eg, drug dose change in response to harms, participant request, or improving/worsening disease)	-	Pg 12
	11c	Strategies to improve adherence to intervention protocols, and any procedures for monitoring adherence (eg, drug tablet return, laboratory tests)	-	Pg 11 (fidelity)
	11d	Relevant concomitant care and interventions that are permitted or prohibited during the trial	-	N/A
Outcomes	12	Primary, secondary, and other outcomes, including the specific measurement variable (eg, systolic blood pressure), analysis metric (eg, change from baseline, final value, time to event), method of aggregation (eg, median, proportion), and time point for each outcome. Explanation of the clinical relevance of chosen efficacy and harm outcomes is strongly recommended	-	Pg 8,9

For peer review only - http://bmjopen.bmj.com/site/about/guidelines.xhtml

SPIRIT | CONSORT OUTCOMES Reporting Guidelines for Trial Outcomes

Section	Item No.	SPIRIT 2013 Item	SPIRIT-Outcomes 2022 item	Location Reported[b]
	12.1		Provide a rationale for the selection of the domain for the trial's primary outcome	Pg 8
	12.2		If the analysis metric for the primary outcome represents within-participant change, define and justify the minimal important change in individuals	Pg 8
	12.3		If the outcome data collected are continuous but will be analyzed as categorical (method of aggregation), specify the cutoff values to be used	Pg 8
	12.4		If outcome assessments will be performed at several time points after randomization, state the time points that will be used for analysis	Pg 8
	12.5		If a composite outcome is used, define all individual components of the composite outcome	N/A
Participant timeline	13	Time schedule of enrolment, interventions (including any run-ins and washouts), assessments, and visits for participants. A schematic diagram is highly recommended (see Figure)	-	Pg 8
Sample size	14	Estimated number of participants needed to achieve study objectives and how it was determined, including clinical and statistical assumptions supporting any sample size calculations	-	Pg 9
	14.1		Define and justify the target difference between treatment groups (eg, the minimal important difference)	Pg 9
Recruitment	15	Strategies for achieving adequate participant enrolment to reach target sample size	-	Pg 5
**Methods: Assignment of interventions (for controlled trials)**				
Allocation:				
Sequence generation	16a	Method of generating the allocation sequence (eg, computer-generated random numbers), and list of any factors for stratification. To reduce predictability of a random sequence, details of any planned restriction (eg, blocking) should be provided in a separate document that is unavailable to those who enrol participants or assign interventions	-	Pg 5

For peer review only

Section	Item No.	SPIRIT 2013 Item	SPIRIT-Outcomes 2022 item	Location Reported[b]
Allocation concealment mechanism	16b	Mechanism of implementing the allocation sequence (eg, central telephone; sequentially numbered, opaque, sealed envelopes), describing any steps to conceal the sequence until interventions are assigned	-	Pg 5
Implementation	16c	Who will generate the allocation sequence, who will enrol participants, and who will assign participants to interventions	-	Pg 5
Blinding (masking)	17a	Who will be blinded after assignment to interventions (eg, trial participants, care providers, outcome assessors, data analysts), and how	-	Pg 5
	17b	If blinded, circumstances under which unblinding is permissible, and procedure for revealing a participant's allocated intervention during the trial	-	N/A
**Methods: Data collection, management, and analysis**				
Data collection methods	18a	Plans for assessment and collection of outcome, baseline, and other trial data, including any related processes to promote data quality (eg, duplicate measurements, training of assessors) and a description of study instruments (eg, questionnaires, laboratory tests) along with their reliability and validity, if known. Reference to where data collection forms can be found, if not in the protocol	-	Pg 8,9
	18a.1		Describe what is known about the responsiveness of the study instruments in a population similar to the study sample	References given for validated instruments used pg 8,9
	18a.2		Describe who will assess the outcome (eg, nurse, parent)	Pg 8,9
	18b	Plans to promote participant retention and complete follow-up, including list of any outcome data to be collected for participants who discontinue or deviate from intervention protocols	-	Pg 9, 12

For peer review only - http://bmjopen.bmj.com/site/about/guidelines.xhtml

SPIRIT | CONSORT OUTCOMES
Reporting Guidelines for Trial Outcomes

Section	Item No.	SPIRIT 2013 Item	SPIRIT-Outcomes 2022 item	Location Reported[b]
Data management	19	Plans for data entry, coding, security, and storage, including any related processes to promote data quality (eg, double data entry; range checks for data values). Reference to where details of data management procedures can be found, if not in the protocol	-	Pg 12
Statistical methods	20a	Statistical methods for analysing primary and secondary outcomes. Reference to where other details of the statistical analysis plan can be found, if not in the protocol	-	Pg 9 and SAP
	20a.1		Describe any planned methods to account for multiplicity in the analysis or interpretation of the primary and secondary outcomes (eg, coprimary outcomes, same outcome assessed at multiple time points, or subgroup analyses of an outcome)	SAP
	20b	Methods for any additional analyses (eg, subgroup and adjusted analyses)	-	SAP
	20c	Definition of analysis population relating to protocol non-adherence (eg, as randomised analysis), and any statistical methods to handle missing data (eg, multiple imputation)	-	Pg 9 and SAP
**Methods: Monitoring**				
Data monitoring	21a	Composition of data monitoring committee (DMC); summary of its role and reporting structure; statement of whether it is independent from the sponsor and competing interests; and reference to where further details about its charter can be found, if not in the protocol. Alternatively, an explanation of why a DMC is not needed	-	Pg 13
	21b	Description of any interim analyses and stopping guidelines, including who will have access to these interim results and make the final decision to terminate the trial	-	pg 5, 6 (pilot)
Harms	22	Plans for collecting, assessing, reporting, and managing solicited and spontaneously reported adverse events and other unintended effects of trial interventions or trial conduct	-	Pg 12 and data management plan

For peer review only - http://bmjopen.bmj.com/site/about/guidelines.xhtml

SPIRIT | CONSORT OUTCOMES
Reporting Guidelines for Trial Outcomes

Section	Item No.	SPIRIT 2013 Item	SPIRIT-Outcomes 2022 item	Location Reported[b]
Auditing	23	Frequency and procedures for auditing trial conduct, if any, and whether the process will be independent from investigators and the sponsor	-	N/A - defined by sponsor / funder
**Ethics and dissemination**				
Research ethics approval	24	Plans for seeking research ethics committee/institutional review board (REC/IRB) approval	-	Pg 12
Protocol amendments	25	Plans for communicating important protocol modifications (eg, changes to eligibility criteria, outcomes, analyses) to relevant parties (eg, investigators, REC/IRBs, trial participants, trial registries, journals, regulators)	-	Pg 12
Consent or assent	26a	Who will obtain informed consent or assent from potential trial participants or authorised surrogates, and how (see Item 32)	-	Pg 5
	26b	Additional consent provisions for collection and use of participant data and biological specimens in ancillary studies, if applicable	-	N/A
Confidentiality	27	How personal information about potential and enrolled participants will be collected, shared, and maintained in order to protect confidentiality before, during, and after the trial	-	Pg 5, PIS and data management plan
Declaration of interests	28	Financial and other competing interests for principal investigators for the overall trial and each study site	-	Pg 13
Access to data	29	Statement of who will have access to the final trial dataset, and disclosure of contractual agreements that limit such access for investigators	-	Data management plan
Ancillary and post-trial care	30	Provisions, if any, for ancillary and post-trial care, and for compensation to those who suffer harm from trial participation	-	N/A
Dissemination policy	31a	Plans for investigators and sponsor to communicate trial results to participants, healthcare professionals, the public, and other relevant groups (eg, via publication, reporting in results databases, or other data sharing arrangements), including any publication restrictions	-	Pg 13
	31b	Authorship eligibility guidelines and any intended use of professional writers	-	Pg 13

For peer review only - http://bmjopen.bmj.com/site/about/guidelines.xhtml

Section	Item No.	SPIRIT 2013 Item	SPIRIT-Outcomes 2022 item	Location Reported[b]
	31c	Plans, if any, for granting public access to the full protocol, participant-level dataset, and statistical code	-	Pg 13
**Appendices**				
Informed consent materials	32	Model consent form and other related documentation given to participants and authorised surrogates	-	Appendix
Biological specimens	33	Plans for collection, laboratory evaluation, and storage of biological specimens for genetic or molecular analysis in the current trial and for future use in ancillary studies, if applicable	-	N/A

[a]It is strongly recommended that this checklist be read in conjunction with the SPIRIT (Standard Protocol Items: Recommendations for Interventional Trials) Statement paper for important clarification on the items. Amendments to the protocol should be tracked and dated. The SPIRIT checklist is copyrighted by the SPIRIT Group under the Creative Commons "Attribution-NonCommercial-NoDerivs 3.0 Unported" license and is reproduced with permission.
[b]Indicates page numbers and/or manuscript location: to be completed by authors.

For peer review only

For peer review only - http://bmjopen.bmj.com/site/about/guidelines.xhtml
