# Supplementary material for: BabyBreathe trial: protocol for a randomised controlled trial of a complex intervention to prevent postpartum return to smoking
Source: BMJ Open. 2023 Sep 4;13(9):e076458. doi: 10.1136/bmjopen-2023-076458 (PMC10481735; doi:10.1136/bmjopen-2023-076458)
Supplement: Reviewer comments [file bmjopen-2023-076458.reviewer_comments.pdf]

PEER REVIEW HISTORY

BMJ Open publishes all reviews undertaken for accepted manuscripts. Reviewers are asked to complete a checklist review form (**http://bmjopen.bmj.com/site/about/resources/checklist.pdf**) and are provided with free text boxes to elaborate on their assessment. These free text comments are reproduced below.

ARTICLE DETAILS

**TITLE (PROVISIONAL)**	THE BABYBREATHETM TRIAL: PROTOCOL FOR A RANDOMISED CONTROLLED TRIAL OF A COMPLEX INTERVENTION TO PREVENT POSTPARTUM RETURN TO SMOKING
**AUTHORS**	Notley, Caitlin; Brown, Tracey; Bauld, Linda; Clark, Allan; Duneclift, Sharon; Gilroy, Vicky; Harris, Tess; Hardeman, Wendy; Holland, Richard; Howard, Gregory; Man, Mei-See; Naughton, Felix; Smith, Dan; Turner, David; Ussher, Michael

This article was not externally reviewed at BMJ Open. Protocols that have been independently assessed prior to submission to BMJ Open are usually fast-tracked to publication on the grounds that further substantial changes will not be possible. This independent assessment will usually be external, independent review for both a substantial grant award from a non-commercial or government funder and ethics approval.
